# Health care providers’ decision-making and early adoption of tenofovir alafenamide for HIV preexposure prophylaxis: An inductive qualitative study

**DOI:** 10.1371/journal.pone.0311591

**Published:** 2024-12-05

**Authors:** Elisabeth Merchant, Patricia Solleveld, Kevin Gibas, Douglas Krakower

**Affiliations:** 1 Department of Medicine, Tufts Medical Center, Boston, MA, United States of America; 2 Tufts University School of Medicine, Boston, MA, United States of America; 3 T.H. Chan School of Medicine, University of Massachusetts, Worcester, MA, United States of America; 4 Department of Medicine, Rhode Island Hospital, Providence, RI, United States of America; 5 Warren Alpert Brown University School of Medicine, Providence, RI, United States of America; 6 Department of Medicine, Beth Israel Deaconess Medical Center, Boston, MA, United States of America; 7 Fenway Health, Boston, MA, United States of America; 8 Harvard Medical School, Boston, MA, United States of America; University of Malaga: Universidad de Malaga, SPAIN

## Abstract

**Background:**

Over the past several years, there have been several changes affecting the available options for oral HIV preexposure prophylaxis, including approvals for tenofovir alafenamide with emtricitabine in 2019 and a generic formulation of tenofovir disoproxil fumarate with emtricitabine in 2020.

**Methods:**

In order to better understand providers’ decision-making processes when deciding between these two drugs for pre-exposure prophylaxis, we conducted semi-structured in-depth interviews with resident, fellow and attending physicians in internal medicine and infectious diseases between May 2020 and March 2021. These interviews were analyzed to identify emergent codes, which were utilized in an inductive thematic analysis to identify major themes pertinent to pre-exposure prophylaxis decision-making.

**Results:**

Of 21 participants, 18 expressed a general preference for prescribing tenofovir disoproxil fumarate with emtricitabine, 2 preferred tenofovir alafenamide with emtricitabine and 1 had no specific preference. Providers perceived similar efficacy of the two formulations, and their clinical decisions were influenced primarily by whether HIV pre-exposure prophylaxis users belonged to a population with an indication for each of the two drugs (e.g. gender-related restrictions for tenofovir alafenamide), the medications’ differing side effect profiles, cost and insurance considerations, prior personal and collective experience with each of these medications, and personal preferences. Respondents also noted that both providers and HIV pre-exposure prophylaxis users were influenced by external factors, including institutional prescribing guidance, advertising, and social influences, including from peers and colleagues.

**Conclusions:**

Our findings suggest that unbiased educational campaigns for both prescribers and users of HIV pre-exposure prophylaxis will be important to support evidence-based prescribing practices and cost-effective decisions among oral pre-exposure prophylaxis options.

## Introduction

There are an estimated 30,635 new cases of HIV annually in the United States, highlighting the need to implement effective HIV prevention strategies [1]. Studies have demonstrated the effectiveness of tenofovir disoproxil fumarate with emtricitabine (TDF/FTC) taken daily as HIV preexposure prophylaxis (PrEP), and there have been very few documented HIV infections in people adherent to PrEP at the time of infection [2]. TDF/FTC taken daily for PrEP was approved by the Food and Drug Administration (FDA) in 2012 for adults, with the indication expanded to include adolescents in 2018 [3].TDF/FTC was initially marketed exclusively under the brand name Truvada by Gilead Pharmaceuticals. Starting in October 2020, a generic alternative became available, and in March 2021 multiple generic options became accessible, greatly reducing its price [4].

The first alternative to daily TDF/FTC to be approved by the FDA for PrEP was tenofovir alafenamide with emtricitabine (TAF/FTC, manufactured by Gilead Pharmaceuticals under the brand name Descovy) in October 2019, approved for adults and adolescents at risk for sexually-acquired HIV, excluding those at risk from receptive vaginal sex [3]. TAF/FTC was demonstrated to be non-inferior to TDF/FTC for the prevention of new HIV infections among men who have sex with men and transgender women in the DISCOVER trial [5]. TAF/FTC was formulated to reduce the potential for adverse renal outcomes and decreased bone mineral density (BMD) associated with use of TDF/FTC when used as part of combination treatment for HIV infection. In studies of PrEP, TDF/FTC has been associated with mild decreases in renal function that typically normalize after discontinuing PrEP, and with decreases in bone mineral density (BMD) not associated with increased fracture rates [6, 7]. In the DISCOVER trial, significant differences were noted in bone and renal biomarkers among those using TDF/FTC versus TAF/FTC; however, there were no differences between these drugs in rates of discontinuing PrEP for bone or renal harms. Overall clinical adverse events were similar between the TAF/FTC and TDF/FTC arms, as TAF/FTC was associated with greater weight gain and altered lipid parameters [5].

Soon after the approval of TAF/FTC for HIV treatment, this formulation largely supplanted TDF/FTC in combination regimens because of its lesser impact on renal and bone biomarkers among people living with HIV, a population at increased risk for renal and bone disease. For PrEP, given the similar safety profiles of these formulations and their use in populations without specific risks for renal and bone disease, cost-effectiveness studies have found that using generic TDF/FTC instead of branded TAF/FTC would offer substantial cost savings [8]. The objective of our study was to examine health care providers’ perceptions, attitudes and prescribing behaviors for TDF/FTC versus TAF/FTC during the first two years following FDA approval of this second PrEP option, which could inform strategies to optimize use and cost-effectiveness of oral PrEP in an era of limited resources for HIV prevention in the US.

## Methods

### Study design and setting

We explored providers’ approaches to prescribing TAF/FTC or TDF/FTC for PrEP using inductive qualitative methods with semi-structured in-depth individual interviews [9]. This method was chosen to provide insight into individual providers’ understanding, attitudes and practices regarding their choice in PrEP regimens. This study was conducted in Boston at Beth Israel Deaconess Medical Center (BIDMC), an academic medical center, and Fenway Health, a community health center specializing in healthcare for LGBTQIA+ populations. The study was deemed exempt by the Institutional Review Boards of Fenway Health (3/2020) and BIDMC (4/2020).

### Researcher characteristics

The research team was composed of an infectious diseases attending physician (D.K), and Infectious Diseases fellow (E.M), an internal medicine resident who subsequently became an infectious diseases fellow (K.G.) and a research assistant (P.S., research assistant to D.K). One of the authors (DK) has published other work on PrEP decision-making, potentially leading to social desirability bias in participants’ responses. To mitigate this potential bias, interviews were not conducted by this investigator, transcripts were anonymized prior to his review, and our team members engaged in reflexivity (i.e. self-conscious appraisal and critique to minimize subjectivity in data analysis and interpretation) to reduce bias from preconceived notions about PrEP options [10]. Moreover, interview questions were asked in a neutral manner, with the same questions asked about both TAF/FTC and TDF/FTC, and participants were asked to provide candid answers in an effort to minimize bias. To further reduce bias the authors engaged in deliberate and frequent reflexivity throughout the study, from the development of the interview guide through the drafting of this manuscript. This included revising documents with an explicit eye towards unbiased and neutral collection, analysis, interpretation, and dissemination of data.

### Participant recruitment and interviews

Between May 2020 and March 2021, we extended email invitations to prescribing clinicians at BIDMC and Fenway Health using purposeful sampling to enroll 1) providers from both health care organizations, 2) faculty and trainees, and 3) primary care providers (PCPs) including internal medicine (IM) residents, and infectious diseases (ID) specialists, to capture multiple perspectives [11]. Most providers sampled had professional relationships with at least one member of the interview team, working at the same medical facility. There were no incentives provided for participation. Eighteen of the 39 providers emailed either did not respond to the email or stated they did not have time to participate. 21 providers responded to the email and agree to participate. No participants dropped out of the study. Participants were informed that the research was investigating decision-making around PrEP prescribing. Interview domains included awareness, perceptions and influences on prescribing TAF/FTC versus TDF/FTC, experiences using TAF/FTC PrEP, current prescribing practices for PrEP, and user experiences with oral PrEP formulations.

Interviews were conducted one-on-one by K.G., (male IM resident) or E.M. (female ID fellow) between May 2020 and April 2021 using secure video chat or in person at the hospital, after obtaining informed consent. Interviews were semi-structure based on a pre-formulated interview guide with each interviewee asked the same set of pre-specified questions, with ad-lib follow-up questions as determined by the interviewer. All interviewees answered all questions posed. Each participant was interviewed only once, with interviews lasting approximately 30 minutes. Interviews were audio-recorded and transcribed verbatim, with hand-written notes taken during the interviews. A brief quantitative survey was used to collect data on participants’ demographics and experiences with PrEP prescribing prior to the interviews. Interviews were conducted until saturation was reached, with no new codes or themes from the last 4 interviews conducted, confirmed with a saturation grid and review by two team members (E.M., D.K.) [12].

This study was deemed exempt by both the Institutional Review Boards of Fenway Health and BIDMC. The study was performed in accordance with the ethical standards as laid down in the 1964 Declaration of Helsinki. Verbal informed consent, including consent for publication, was obtained prior to the interview from all individual participants included in this study.

### Data analysis

Interview transcripts were analyzed using DeDoose software. Interview transcripts are available in S1 File. The researchers first assembled a preliminary list of codes based on extraction of concepts relating to our research questions during a detailed review of the raw data (i.e., interview transcripts). Next, the researchers together engaged in data reduction by simplifying, combining or renaming codes from the full list to be as parsimonious as possible without compromising comprehensiveness. Codes were categorized according to their relationship to the domains of our semi-structured interview guide as well as additional, unanticipated categories pertinent to our research aims. Finally, emergent themes relating to our research questions were described by the researchers based on these categories, and these themes were revised in an iterative manner through ongoing discussions amongst team members, including with application of reflexivity to mitigate bias. All transcripts were then coded by E.M., with a subset of 20% separately coded by P.S. to assess inter-rater reliability. E.M., P.S. and D.K. reviewed each incidence of discrepant coding and came to an agreement on the appropriate code. An inductive thematic analysis was utilized to identify a thematic framework from the codes [9].

## Results

### Survey of provider characteristics

39 participants (7 ID attendings, 17 attending PCPs,10 ID fellows, and 7 IM residents) were invited via email and 21 enrolled (2 ID attendings, 5 attending PCPs, 8 ID fellows, 6 IM residents). A majority of participants were under 32 years-old (13, 61.9%), cis-gender men (12, 57.1%), and white (11, 52.4%). (Table 1) Most participants had been practicing for 0–5 years (16, 76.2%), and nearly provided care for people on PrEP (17, 81%) or living with HIV and on antiretroviral treatment (19, 90.4%).

**Table 1:** Participant characteristics and characteristics of those who prefer TDF/FTC and TAF/FTC.

	All participants N (%)	Providers who prefer TDF/FTC (n = 18)	Providers who Prefer TAF/FTC (n = 2)
**Age (years)**			
28–32	13 (61.9%)	10 (55.6%)	2 (100%)
33–37	4 (19%)	4 (22.2%)	0
43–47	2 (9.5%)	2 (11.1%)	0
≥48	2 (9.5%)	2 (11.1%)	0
**Gender**			
Cis-gender male	12 (57.1%)	10 (55.6%)	1 (50%)
Cis-gender female	9 (42.9%)	8 (44.4%)	1 (50%)
**Race/Ethnicity**			
White	11 (52.4%)	8 (44.4%)	2 (100%)
Asian	7 (33.3%)	7 (38.9%)	0
Hispanic or Latino	1 (4.8%)	1 (5.6%)	0
Something else	1 (4.8%)	1 (5.6%)	0
Decline to answer	1 (4.8%)	1 (5.6%)	0
**Training Level**			
Attending	7 (33.3%)	7 (38.9%)	0
Fellow	8 (38.4%)	6 (33.3%)	2 (100%)
Resident	6 (28.6%)	5 (27.8%)	0
Practice Setting*			
Academic medical center	19 (90.5%)	16 (88.9%)	2 (100%)
Community health center	7 (33.3%)	7 (38.9%)	0
**Specialty**	2 (9.5%)		
Internal medicine	11 (52.4%)	10 (55.6%)	0
Infectious diseases	10 (47.6%)	8 (44.4%)	2 (66.7%)
**Years in practice (since completion of training)**			
0–5	16 (76.2%)	13 (72.2%)	2 (100%)
6–10	2 (9.5%)	2 (11.1%)	0
11–15	0	0	0
15+	3 (14.3%)	3 (16.7%)	0
**Estimated number of people to whom they have prescribed PrEP (past 12 months)**			
0	4	2 (11.1%)	1 (50%)
1–10	8	7 (38.9%)	1 (50%)
11–19	5	5 (27.8%)	0
20–49	3	3 (16.7%)	0
50+	1	1 (5.6%)	0
**Estimated number of people with HIV to whom they have prescribed antiretroviral treatment (past 12 months)**			
0	2	2 (11.1%)	0
1–10	4	3 (16.7%)	0
11–19	5	4 (22.2%)	1 (50%)
20–49	7	6 (33.3%)	1 (50%)
50+	3	3 (16.7%)	0

*Some participants practiced at both the academic medical center as well as the community medical center. TDF/FTC, tenofovir disoproxil fumarate with emtricitabine; TAF/FTC, tenofovir alafenamide with emtricitabine; PrEP, pre-exposure prophylaxis.

Two providers indicated that they preferentially used TAF/FTC (ID fellows at the academic medical center), 18 providers preferred TDF/FTC and 1 provider did not express a preference (IM resident at the academic medical center) (Table 1).

### Thematic analyses

We identified 7 major themes relating to providers’ selection of TAF/FTC versus TDF/FTC; we describe these and provide illustrative quotes in Table 2. Providers perceived similar efficacies between the two drugs. Many providers noted that they preferred TDF/FTC due to FDA approval for a broader range of populations. Discussion of side effect profiles was a common theme, with providers noting concerns over potential renal and bone harms with TDF/FTC and concerns about metabolic toxicities with TAF/FTC. Providers cited greater experience with the safety and efficacy of TDF/FTC as a factor favoring this formulation. On the other hand, providers incorporated PrEP user knowledge and preferences and noted greater PrEP user awareness of the side effects associated with TDF/FTC versus TAF/FTC. Financial and insurance issues were felt to favor TDF/FTC, largely related to the recent release of its generic formulation. Providers identified several external factors that influenced the decision between TDF/FTC and TAF/FTC including institutional prescribing guidance, advertising, and social influences, including from peers.

**Table 2:** Major emergent themes and illustrative quotations.

**Theme 1: Providers believed that TDF/FTC and TAF/FTC have similar efficacy.**	
“If you have a medication which is safe and well-tolerated, and equivalent, say to an alternative that’s much more expensive, I don’t see any great need to switch.” *(Infectious diseases fellow)*	
**Theme 2: Providers favored TDF/FTC for PrEP because it is FDA-approved for all populations**	
“I probably would be a little bit less likely to prescribe in cis-female, though, again, I think if there were comorbidities there that might increase their risks of renal side effects with TDF, you know there would be a consideration, but it’s kind of weighing the risk/benefit in patient preference in that situation.” *(Infectious diseases attending)*	
**Theme 3: Differing safety profiles was the primary consideration when choosing between TDF/FTC and TAF/FTC.**	
*Nephrotoxicity with TDF/FTC*	*“I might think about*, *for clinical reasons*, *like renal function*. *I had one patient who we didn’t do it (prescribe TDF) because they only had one kidney’ (Infectious diseases fellow)*
	“Maybe if they had uncontrolled diabetes, hypertension with a significant proteinuria at baseline, I might be concerned about their risk factors for a second hit to the kidneys with TDF.” *(Attending primary care provider)*
*Concerns about bone mineral density with TDF/FTC*	“Do they also have risk factors for osteoporosis? Have they been on chronic steroids in the past? Things like that. Are they a smoker? Are they frail? You know, then I might be more willing to switch them over to TAF/FTC.” *(Attending primary care provider)*
*Reversibility of TDF/FTC toxicities*	“I believe that the alafenamide might… have a little bit less renal toxicity compared to the disoproxil, because I know that that was like a big concern even though it’s like largely thought to be reversible and then I know the other side effect, at least with the disoproxil is bone health… like bone resorption and osteoporosis and all that stuff. And again, that’s something that I believe is not an irreversible thing. When you stop it gets better.” *(Internal medicine resident)*
	“I present to them some of the data from the iPrex study, you know I let them know that a very small minority developed renal dysfunction, and that after stopping Truvada, almost a large majority of them completely had recovery of their renal function, and then also with the loss of bone mineral density I just let them know that there was, in terms of clinical significance, there were no clinically significant pathologic fractures, things like that..” *(Attending primary care provider)*
*Metabolic concerns with TAF/FTC*	“However, what we’ve seen a lot of is people with unwanted weight gain, not a ton, but you know like, significant to patients, on TAF. Given that it’s a young population for the most part who’re very sensitive to body image, I’ve had very few people who want to go with TAF.” *(Attending primary care provider)*
	“Just considering if this is something that they’re gonna be on long term or that they’re gonna want to take long term, especially considering that the renal impairment and the bone changes with the disoproxil form are reversible. In terms of the lipid stuff, my guess is it would probably be somewhat reversible with stopping. But, I mean weight gain is also weight gain and It’s hard for people to lose weight.” *(Internal medicine resident)*
*Age and anticipated duration of PrEP use*	“The theoretical but very low risk of renal injury on TDF, the bone mineral stuff I have not found to be especially relevant clinically for this young population.” *(Attending primary care provider)*
	“The TAF preparation could have less effect on bone loss and maybe less effect on renal dysfunction… I probably think of [those side effects] a little bit more if the patient is really young. Like I think for adolescents and young twenty-something year olds that may be on it for a while.” *(Infectious diseases fellow)*
	“An older person where, even if they have had reasonable renal function and okay bone mass, you might consider… the TAF regimen, just for concern about long-term side effects if the patient was going to be on PrEP.” *(Infectious diseases fellow)*
	“It (TAF/FTC) causes dyslipidemia, or there’s some concern about it having effects, like cardiovascular effects…Honestly most of my patients are really young and pretty healthy,…, but if I had a patient who was a lot older, had like really uncontrolled hyperlipidemia, or like really strong history of CAD (coronary artery disease) or something like that, I might reconsider, but I have so few patients that fit that bill.” *(Internal medicine resident)*
	“The risks of all of the side effects go up with age. So, I don’t think age plays a great deal in it, you know, like specific comorbidities do, which increase in everyone in age. Like, if you’re at higher risk of adverse coronary events and older, I would lean away from TAF. But these are the same people that have a higher risk for renal dysfunction.” *(Internal medicine resident)*
*Concerns about tolerability*	“I think my experience is that there were maybe fewer GI side effects from the drug (TAF/FTC)… but I have way more people on TDF than TAF, and so it just may not be that I’ve had enough patients on TAF to be able to pick up whether there is some GI intolerability.” *(Attending primary care provider)*
	“TDF is large, some patients won’t take pills because they’re very big, and TAF is smaller. So that can make a very big difference, and of course it’s all about adherence, it doesn’t matter which one I give them if they don’t take it.” *(Attending primary care provider)*
**Theme 4: Providers favored TDF/FTC because of greater experience and familiarity with this medication**	
“I would prescribe TDF/FTC at this point, just because we have a long track record with using TDF/FTC, we have a lot of data to support its efficacy, there doesn’t seem to be, in patients that don’t have underlying renal disease, there doesn’t seem to be a huge propensity for harm with this regimen, and most providers have substantial experience with it.” *(Infectious diseases fellow)*	
“I still feel like I end up prescribing TDF based, but… there’s nothing specific that would keep me from picking a TAF-based regimen. So I think it would, probably just old habits and reflex is the only thing at this point.” *(Infectious diseases fellow)*	
**Theme 5: Providers integrated PrEP users’ preferences into prescribing decisions, in particular ‘ knowledge of potential toxicities with TDF/FTC and interest in using TAF/FTC.**	
*Variability in PrEP user knowledge*	“I honestly don’t know if they are even aware that there are differences. I think most of my patients are just like “Oh there’s PrEP” and they just want to start PrEP, but they don’t even know that there are two different options within that.” *(Internal medicine resident)*
	“Patients usually have like flyers, or they’ve looked it up. I mean, I think it’s because of whatever community that they connect with, but it is very interesting to see how they’ve tried to be really thoughtful and bring that forward, even though I feel like there’s really not a lot of info out there, for like non-medical providers. Like, if, I feel like if you were just a patient it would be really hard to know what’s the difference.” *(Infectious diseases fellow)*
*Prior experiences with TDF/FTC*	“I think some patients have been on PrEP for several years now, which is great. It’s worked for them. They have no side effects. Maybe they get it through a copay assistance program or it’s free. It’s sort of their routine and they don’t see any problems so they want to keep on it and that’s fine.” *(Infectious diseases fellow)*
	“They want to be on the thing that has been on the market longer, and we have more experience prescribing, and we have sort of like more data to see what it does over a longer period of time. So, you know I give the option to my patients but I definitely always say like ‘my first choice is Truvada, it’s what I’ve prescribed most, it’s what I’ve seen most’.” *(Internal medicine resident)*
*PrEP user interest in TAF/FTC*	“There was a lot of hype, and when the Descovy studies got published or announced and there were people who were looking to be on the newest agent. So there was some patient pressure to switch.” *(Attending primary care provider)*
*More awareness of TDF/FTC toxicities*	“I always get the question ‘is this safe for my kidneys. . . . So I think patients have a lot of awareness, they’ve heard a lot through either the media, um you know, certainly with the lawsuits against [the pharmaceutical company] and just information that they’ve read online. I think that’s the most common thing. And I do find that patients are also aware about the potential side effect with bone mineral density too. Patients are pretty savvy about that.” (Attending primary care provider)
	“I did have one patient just ask me point blank, like ‘isn’t this (TDF/FTC) the ‘Old Stuff’, like aren’t there other, newer, things we could try?’. . . I told that patient there’s really nothing keeping them, if they wanted to change it was perfectly fine, but there was nothing to suggest that it was, just because it was older that it would be less effective. And so ultimately, the conclusion was that he was kept on TDF.” (Infectious diseases fellow)
	“Word is out on TDF for sure. TAF, I don’t feel like word is out as much. I don’t feel like my patients know that there are downsides to TAF.” (Infectious diseases attending)
	“I have not had any patient know of any of those adverse effects (associated with TAF/FTC). And actually I didn’t know about those until I started talking more to our preceptors about it when that initial study came out about non-inferiority. . . So, I don’t think it’s widely known in the general community of people who might benefit from PrEP.” (Internal medicine resident)
*Shared decision-making*	“I have not yet necessarily sat with a patient who wanted to be on PrEP and said, ‘We have this formulation of tenofovir and we have this formulation of tenofovir…. We use them in both the same way, do you have a preference based on the potential side effects’. Mostly I have said ‘I would recommend this drug, for this reason.’” (Infectious diseases attending)
	“There have been a few patients who have just heard that it’s (TAF/FTC) is safer and want to switch, and I was unable to convince to stay on Truvada.” (Attending primary care provider)
**Theme 6: Financial and insurance considerations favored TDF, especially with the approval of generic TDF.**	
“I would definitely explore that option (generic TDF/FTC), because of a better chance of availability for affordability and access, easy access for a generic versus more brand name form. Just a general experience, medications with generic tend to be more available, easier insurance approvals.” (Infectious diseases fellow)	
“The bigger experience (switching to TAF) has been that they have to jump through hoops in terms of getting it, continuity can sometimes be an issue because of the prior authorization. They ask me for it when they have 4 pills left of their TDF, and I can’t get them the TAF in 3 days.” (Attending primary care provider)	
“I have not personally run into situations where cost was prohibitive for a patient. Either patients were able to afford the medications’ co-pay, or we have gotten them PrEP-DAP. . . . Or used, previously before TDF was generic, just used the… coupon.” (Attending primary care provider)	
**Theme 7: Providers believed that PrEP recommendations were influenced by professional norms, institutional guidance, educational resources, pharmaceutical advertising to PrEP uesrs and social influences.**	
“In general, my first choice is Truvada, and I think that’s basically based on just the fact that that’s like what’s used most at [my institution].” (Internal medicine resident)	
“Most of the patients have seen commercials and seen advertisements about [TAF/FTC] for PrEP. So sometimes they often ask me about it.” (Infectious diseases fellow)	
“The patient came to clinic and wants to start PrEP and had talked to their friends and their friends said ‘Have you heard of the TAF-containing regimens?’” *(Infectious diseases fellow)*	

TDF/FTC, tenofovir disoproxil fumarate with emtricitabine; TAF/FTC, tenofovir alafenamide with emtricitabine; PrEP, pre-exposure prophylaxis.

#### Theme 1: Providers believed that TDF/FTC and TAF/FTC have similar efficacy

Many providers perceived that the two drugs were of similar efficacy and none expressed concerns that either drug was less efficacious. (Table 2) *“In my mind TAF and TDF are equivalent apart from their side effect profile[s]*.*” (ID fellow)* Given similar efficacy, providers used other factors to drive decisions, such as side effects and cost.

#### Theme 2: Providers favored TDF/FTC for PrEP because it is FDA-approved for all populations

Providers noted that TAF/FTC has currently only been studied in and approved for people who do not engage in receptive vaginal sex (e.g. men who have sex with men [MSM] and transgender women [TGW]), which contributed to their preference for TDF/FTC. *“The DISCOVER trial was limited… only using MSM and trans women*, *so we don’t know essentially yet if it’s generalizable*, *so I wouldn’t use it also for those practicing receptive vaginal sex*.*” (Attending primary care provider)* Some providers felt that based on its more limited evidence-base, they would not use TAF/FTC in people with HIV risk factors outside of its approved indications, such as vaginal sex or injection drug use. However, other providers felt that the limited evidence and lack of FDA approval for use of TAF/FTC for people who engage in vaginal sex or people who inject drugs (PWID) might be outweighed by other considerations, such as renal comorbidities.

#### Theme 3: Differing safety profiles was the primary consideration when choosing between TDF/FTC and TAF/FTC

Every provider mentioned they would consider side effect profiles and user comorbidities when choosing between TDF/FTC and TAF/FTC. A dominant theme was how the potential nephrotoxic effects of TDF/FTC affected decision-making, particularly for people with or at risk for renal dysfunction, however concerns about decreased BMD with TDF/FTC were also commonly noted. *“I think if [a patient] already has a GFR that is impaired*, *if they’re in that 30–60 range for their GFR*,… *I think that TAF is reasonable*.*” (Internal medicine resident)* Providers considered the potential toxicities associated with TDF/FTC to be reversible or clinically insignificant, which mitigated concerns. While nephrotoxicity and bone harms were the primary concerns with TDF/FTC, weight gain and metabolic effects were among the most commonly cited concerns about TAF/FTC. Some expressed beliefs that the weight gain was more clinically significant and less reversible than the side effects associated with TDF/FTC. *“I think if someone was overweight or obese or had family history*,… *it might make me move towards the Disoproxil formulation*… *especially considering that the renal impairment and the bone changes with the disoproxil form are reversible*… *But*, *I mean weight gain is also weight gain and It’s hard for people to lose weight*.*” (Internal Medicine Resident)*

Providers cited age and anticipated duration of PrEP use as factors in drug selection, although there was not a consensus as to the optimal drug for younger or older PrEP users. The perceived younger age and good health of PrEP users assuaged some providers’ concerns about TDF/FTC toxicities. However, other providers felt that younger people might have a longer duration of treatment and therefore favored the diminished renal and bone toxicities of TAF/FTC, analogous to their approach for people living with HIV. Some providers felt that older age raised concerns about the potential renal and bone toxicities of TDF/FTC, while others felt older age was a reason to avoid potential metabolic toxicities with TAF/FTC. Some felt age did not influence their decision between TAF/FTC and TDF/FTC, because all toxicities could increase with advanced age.

A minor consideration was perceived tolerability, either due to gastrointestinal (GI) side effects associated with TDF/FTC or the smaller pill size of TAF/FTC.

#### Theme 4: Providers favored TDF/FTC because of greater experience and familiarity with this medication

A recurrent pattern was that positive first-hand experiences and longer collective duration of prescribing TDF/FTC for PrEP increased comfort with this formulation. Some providers felt that habit contributed to their continued use of TDF/FTC. *“I’m still pretty much prescribing Truvada*,… *because that’s what I’ve always done*, *and it’s what was out there first*. *And I feel like the number of patients that I’ve had on it*, *I think I’ve only had one patient so far that had any um*, *renal impairment*, *that has had any toxicity that I can measure from it*. *Despite having patients that have been on it for*, *you know*, *years*, *for prevention*. *And so I still feel pretty safe with it*.*” (Infectious diseases attending)*

#### Theme 5: Providers integrated PrEP users’ preferences into prescribing decisions, in particular knowledge of potential toxicities with TDF/FTC and interest in using TAF/FTC

Providers described varied levels of PrEP user knowledge about the differences between TDF/FTC and TAF/FTC, from no awareness to being well-informed. This knowledge was perceived as coming from social networks, advertisements, personal research and experience. PrEP user inquiries or requests were more common for TAF/FTC, and people were particularly aware of the potential toxicities of TDF/FTC. This knowledge was perceived to come largely from friends, media, and awareness of lawsuits claiming damages from use of TDF/FTC. Some PrEP users seemed to assume that TAF/FTC was “safer” than TDF/FTC because it is “new”. Providers felt that there was less recognition among PrEP users of metabolic toxicities with TAF/FTC.

Providers varied in their use of provider-dominant, patient-dominant or shared decision-making for PrEP selection. Some providers initiated the discussion of the two options routinely, while others only addressed options when brought up by patients. *“I usually have a conversation with the patient and ask them if they’ve heard of both… Which sometimes they have*, *and then*, *kind of explain the differences*.*” (Infectious diseases fellow)* When providers’ and PrEP users’ preferences differed, the general approach was to defer to PrEP users.

#### Theme 6: Financial and insurance considerations favored TDF/FTC, especially with the approval of generic TDF/FTC

Cost and insurance coverage played a role in providers’ decision-making, favoring TDF/FTC, which was perceived to be more cost-effective, in part related to the availability of generics. *“The big concern would be that we would prescribe it (TAF/FTC) and the patient couldn’t afford it and maybe wouldn’t come back to clinic and kind of lose that ability to capture and establish care with them*.*” (Infectious diseases fellow)* Some providers had PrEP users who had experienced these barriers with TAF/FTC, while others had not, citing financial support systems.

#### Theme 7: Providers believed that their PrEP recommendations were influenced by professional norms, institutional guidance, educational resources, and pharmaceutical advertising to PrEP users

When asked about sources of influence for prescribing decisions, providers cited guidance from within their own institutions, clinical practices of colleagues (or preceptors for trainees), or didactic education. Many used online resources, including professional society guidelines, resources such as UpToDate.com, or literature reviews. Additional influences on providers included social media and pharmaceutical companies. Providers described direct-to-consumer advertising as an influence on PrEP users, acknowledging personal consumption of the same advertising seen by PrEP users; however, they did not identify their own exposure to advertising as a significant influence on their decisions. *“It’s hard to avoid seeing it on commercials*, *but I don’t use commercials as a management tool*.*” (Attending primary care physician)* This is consistent with prior studies showing that physicians are susceptible to influence from pharmaceutical advertising, although they may not recognize this influence [13]. A perception existed that a shift in advertising toward Descovy (TAF/FTC) from its manufacturer may have been spurred by the waning patent protection for TDF/FTC prior to the release of generic formulations.

## Discussion

The availability of new PrEP formulations has the potential to improve its use, safety and impact, if new formulations offer advantages over existing options. However, it can also result in increased use of less cost-effective medications without population benefits. We interviewed primary care and infectious diseases providers about their experiences and approaches to deciding between TDF/FTC or TAF/FTC for PrEP, to learn about whether and how the availability of this new option might impact prescribing behaviors.

Our main themes suggest that providers were confident in the efficacy of either regimen for MSM. For some providers, a lack of proven efficacy for TAF/FTC in other populations, such as people engaging in vaginal sex or PWID, was an absolute contraindication, however others suggested they would consider off-label use of TAF/FTC in the setting of certain risk factors. A universal decision-making approach was to consider PrEP users’ comorbidities and the side effect profiles of PrEP formulations when deciding between TAF/FTC and TDF/FTC, with avoidance of TDF/FTC in people with a diagnosis of or risk factors for renal dysfunction or low bone mineral density. Some providers also avoided TAF/FTC in people with obesity or metabolic syndrome given potential for weight gain and dyslipidemia. Age was a common consideration in PrEP selection, however, given longer expected duration of use among younger people and increased comorbidities among older people there was not consensus about the optimal formulation to use at either end of the age spectrum.

PrEP user interest in TAF/FTC, generated by media, advertisements and social networks, influenced providers towards its use. This influence seemed to be balanced by providers’ concerns that PrEP users would ultimately wish to avoid the metabolic toxicities of TAF/FTC, particularly weight gain, even though people were less familiar with these effects than with potential harms from TDF/FTC. When met with uncertainty about the optimal choice for individual PrEP users, or when there was discordance between provider and PrEP user preferences, some providers asserted their recommendation, while others engaged in shared decision-making and many deferred to user preferences. Our findings suggest that educational messaging directed at PrEP users impacts prescribing decisions. Because direct-to-consumer advertisements by drug manufacturers may be biased towards drugs that increase their revenue, it will be in PrEP users’ best interests to disseminate unbiased information about PrEP options to them, such as through public health campaigns, as well as to providers through clinical training.

In an observational study in which 277 of 1009 people surveyed in 2019–2020 had switched from TDF/FTC to TAF/FTC, 56% indicated that the decision to switch was provider-led (without further details), 32% perceived TAF/FTC to be safer, 8% preferred its smaller pill size, and 6% had side effects or pre-existing medical conditions that influenced the switch. In a study of an online screening questionnaire for a trial of a PrEP adherence intervention in early 2020, most people were prescribed TDF/FTC, and self-reported PrEP adherence was greater with TAF/FTC than TDF/FTC, which was speculated to be related to treatment fatigue with TDF/FTC or beliefs that TAF/FTC had superior efficacy or improved side effect profile. Our study is consistent with these studies in that perceptions of differing safety profiles between TAF/FTC and TDF/FTC, currently seen through the lens of the providers, may promote continued use of TDF/FTC for most PrEP users and switches to TAF/FTC driven by perceptions of improved safety or smaller size.

Our finding for a provider preference for TDF/FTC in 2020 is consistent with studies published in 2021 showing that TDF/FTC still being prescribed as PrEP in a majority of cases [14, 15]. Subsequent data, though, has shown the growth of TAF/FTC prescribing for PrEP, with a retrospective observational study showing that TAF/FTC was prescribed in 86% of new PrEP starts between October 2019 and May 2021 [16]. An abstract published in 2023 analyzing the IQVIA Real-World data—Longitudinal Prescription Database found that there were more prescriptions for TAF/FTC than brand-name TDF/FTC from late 2020 until September of 2021, at which time generic TDF/FTC became the most commonly prescribed drug (currently representing 50.3% of prescriptions for PrEP, with TAF/FTC representing 45% of prescriptions, brand-name TDF/FTC 4.5% and injectable cabotegravir 0.5%) [17]. These findings are in concordance with our identified theme that a generic option for TDF/FTC moves providers towards this choice. However, it is striking that nearly half of all recent PrEP prescriptions were TAF/FTC despite the availability of a generic option of similar safety and efficacy, suggesting a need for further studies to understand how providers and PrEP users approach prescribing decisions for PrEP, to improve its cost-effectiveness.

Our study design has limitations. We enrolled providers from only two affiliated institutions in Boston, so our findings may not be generalizable outside of these two institutions, especially given the contributions of institutional culture on PrEP decision-making described by participants. The two institutions included represent a prototypical academic medical center and a community health center that is the largest PrEP provider in New England.

Additionally, interviews for this study were conducted during the time period in which generic TDF/FTC first became available (with a minority of interviews being conducted prior to availability of the generic), and views surrounding generic options may have changed over time.

Another limitation is that our data did not reveal themes relating to the influence of sociodemographics on PrEP prescribing decisions, which may have been due to a lack of attention to this topic in our interview guide. However, given prior work demonstrating that providers may have implicit biases that influence their prescribing of PrEP, such as being less likely to offer PrEP to Black individuals, future studies focused on how providers select among PrEP options should explore the topic of how sociodemographic factors affect prescribing decisions in more detail [18].

This study was also conducted prior to the 2020 approval of long-acting injectable cabotegravir (CAB-LA) given every 8 weeks for PrEP [19]. We additionally did not query providers on non-FDA-approved PrEP alternatives which have been shown to be effective in certain populations, including on-demand TDF/FTC PrEP (currently recommended by the World Health Organization [WHO]), and the dapivirine vaginal ring (currently recommended by the WHO but not available in the US) [20, 21].

This study highlights the need for further study into the providers’ decision-making processes around PrEP at other institutions and in other regions. Additionally, an important next step will be to further investigate the PrEP user perspective, which is only evaluated indirectly in this study.

In conclusion, we found that in the first two years after the availability of TAF/FTC for PrEP, providers at our institutions still generally preferred to prescribe TDF/FTC despite considering potential renal and bone adverse effects. Preferences for TDF/FTC were driven by perceptions that the potential harms from TDF/FTC were reversible and of minimal clinical significance, as well as familiarity with this formulation, approval for use in all populations, and fewer concerns about cost or insurance coverage. TAF/FTC was preferred primarily in people with risk factors or comorbidities that would put them at higher risk for renal or bone adverse effects, especially because providers also had concerns about metabolic toxicities from its use. However, providers experienced interest in TAF/FTC from PrEP users, which is perceived as being driven by social influences and advertising, which could also drive increased use of this formulation for clinicians who support shared decision-making with PrEP users. Our findings suggest that unbiased educational campaigns for providers and PrEP users will be important to support evidence-based prescribing practices and cost-effective decisions about oral PrEP options given the dramatic cost differences between generic TDF/FTC and branded TAF/FTC.

## Supporting information

S1 File Interview transcripts. (ZIP)

S2 File Human subjects research checklist. (DOCX)
