# Supplementary material for: Health care providers’ decision-making and early adoption of tenofovir alafenamide for HIV preexposure prophylaxis: An inductive qualitative study
Source: PLoS One. 2024 Dec 5;19(12):e0311591. doi: 10.1371/journal.pone.0311591 (PMC11620414; doi:10.1371/journal.pone.0311591)
Supplement: S1 File — (ZIP) [file pone.0311591.s001.zip › Clean transcripts/DedooseDoc_Participant 1 Transcript.docx]

Subject 01 Interview

Interviewer: Okay, thank you for going through the consent with us and participating today. Let's get started. The first question that our research team came up with is regarding the differences between tenofovir disoproxil fumarate and tenofovir alafenamide fumarate. So I am going to ask you a few questions about the use of tenofovir disoproxil fumarate and tenofovir alafenamide fumarate for prep. So first have you heard of using the 2 formulations of tenofovir for prep.

Subject 01: Yes.

Interviewer: Great! What are some of the things that you have heard about for TAF (or tenofovir alafenamide) vs. TDF, the disoproxil form?

Subject 01: I think that it (TAF) was originally formulated because of the renal and bone density concerns with TDF. And we've pretty much switched every single HIV medication over to a TAF containing regimen in the past like 5 years. You know, for those exact risk reasons. And you know all of the data for PrEP was in TDF until very recently... until really the past year, that (TDF) had been the standard of care for PrEP. And then there was a study that came out this year showing non-inferiority with TAF. So now that (TAF) is an option. Gilead is pushing it really hard. Another thing that I have heard, and that I see, is that TAF has some adverse effects on weight and metabolic profiles that could be pretty clinically significant, especially for the PrEP population. So that's sort of the other concern.

Interviewer: Okay so based on what you've heard and those things you just mentioned, what are your thoughts on using one versus the other, either TAF or TDF for PrEP? What are some of the things you consider?

Subject 01: Well I and most of the preceptors prescribing (PrEP) still do a lot of TDF for almost everyone. And it (TDF) has a long record of safety and effectiveness at this point. It has some monitoring parameters that we use, but that's it. You know I haven't seen TAF. I haven't had anyone really request or ask about it, but I think the concern for weight and metabolic problems is large enough that we avoid using it.

Interviewer: Okay, great. Are there any specific situations where you would go to TAF versus TDF. I mean it sounds like most of your patients are on TDF. But, what are some of the things that would prompt you to use TAF?

Subject 01: Yes, I think it's kind of 2 things... I think it they (the patient) already has a GFR that is impaired, you know if they're in that 30-60 range for their GFR, and they would still benefit from TAF, I think that TAF is reasonable. Also, if they have substantial risk for bone loss, like if they're on chronic steroids, or if they have been on steroids for a long period of time, that's another time to consider TAF. Those are kind of the two main considerations. Or you know if they (the patient) have shown some mild, like not super super significant, decrease in renal function on TDF. I actually have not seen that, that would be another time you could consider using TAF.

Interviewer: Okay perfect, so on the flip side what are some of the reasons or patient characteristics that would influence you to avoid TAF? I know you have already mentioned some of the concerns you have, but what are some of the reasons that you would avoid it?

Subject 01: I think that especially for someone on the line of having metabolic syndrome, features like pre-diabetes and dyslipidemia, those are the people that I would want to be avoiding it most. You know anyone who is concerned about unwanted weight gain, which you know is the majority of active MSM. Those are all people I would avoid giving it (TAF) to.

Interviewer: I think you mentioned this, but have you had any patient's ask questions or ask for one versus the other (TAF vs. TDF)?

Subject 01: Yeah, we've sort of gone over it. You know at this point I think it is fair to bring up both options during the discussion. You know I frame as TDF generally being my preferred option for these exact reasons. I think have had 2 conversations… one person had asked about switching I think but ended up not.

Interviewer: Okay, alright. And then just some other considerations. Have patients asked you about cost or insurance coverage?

Subject 01: You know it’s never been an issue getting insurance coverage for them. I think that in general when generic tenofovir-emtricitabine formulations come on the scene it will be a bigger thing.

Interviewer: Okay perfect! This is a question I meant to ask a little earlier, but what are some of the sources that you get your information about different formulations of PrEP?

Subject 01: Well I think, there were two recent studies that I can’t remember the name of that came out. Ugh what is the name of it? There was a recent study that came out showing weight gain. I can’t remember the name of it. There have been a couple little ones (studies) that I have read, as well as some anecdotal evidence by providers. Maybe it was the ADVANCE study that showed that (weight gain). And then you know there’s the study that showed they were equivalent, or non-inferior. And then like for PrEP there are all of the classic ones. Then there’s a lot of anecdotal stuff about weight gain with TAF, with not just PrEP patient’s but with HIV positive patients. I think that’s been a part too, that that’s been very common. So in seeing that, that’s kind of carried over.

Interviewer: Great, great. So in your primary care clinic have you received any guidelines or directions on using one versus the other.

Subject 01: Ummm, nothing specific, but she never prescribes TAF unless a patient specifically asks for it and they go over the risks or they have a couple very clear reasons for the switchover.

Interviewer: Okay, in terms of you personally, and sorry if you already answer that, but do you have any patient’s on TAF in your panel?

Subject 01: No

Interviewer: Ummm great. We already talked about some of your preferences, and kind of what patient’s are asking about. For each of TAF and TDF are there any other benefits you consider? Besides renal and bone health, are there any other benefits you think of with TAF?

Subject: Well I think the pill is a little bit smaller but, that’s about it.

Interviewer: And for the patients that you've that you have on PrEP who have asked about switching or for patients who are newly starting prep, do you find patients are asking about the two different ones (TAF vs. TDF) and do your patients have any specific concerns that they bring up often? Whether it's effectiveness, side effects, insurance coverage, or is it just a general curiosity?

Subject 01: No, I think that people are interested about the side effect profile. I think that there's this idea that there are some serious renal side effects from TDF. So, people ask about that. I think that’s the main thing people talk about, is the renal dysfunction. People ask if there is a difference in the GI stuff. Which I don't think. I don’t think there’s enough evidence to say that there's any difference in GI side effects. So people ask about that. And then with insurance, that comes up with everyone. In Massachusetts. I've never had anyone have a problem getting on either, well on TDF. But I’ve never heard of anyone having a problem getting on Descovy either. Again, once a generic is out I can imagine insurance companies saying we’re going to just give you the generic one, which would be reasonable for most people in my opinion.

Interviewer: In thinking about the availability of generics, would either of these influence how you prescribe or what you prescribe?

Subject 01: Yeah. I mean, it would be different if there was more of a clinical concern away from TDF, like if there was like a stronger sign to you know that TAF was more beneficial I would certainly weigh it more heavily. But, I already think that TDF is effective and safe. For the vast majority of people I think a generic is great. These medications are incredibly expensive and somebody is carrying that cost, you know. And, having a generic on the market would be wonderful.

Interviewer: Yeah absolutely. And have you had any of your colleagues, whether you're preceptors or residents, raise specific questions or concerns about this or, you know, asked about guidelines or things like that?

Subject 01: A couple of other residents asked me about it when they were starting people (on PrEP). Usually they asked me if their preceptor was someone who didn’t do a lot of PrEP prescribing. I think sometimes people would just choose it (TAF) just because this is the new one and they assume since this is the new one, it’s equivalent or safer. So I talked to some other people that don’t have a lot of HIV positive patients or not a lot of patients on PrEP, and for some of them their decision would seem like a no brainer as they assumed this was safer, so they would just start that (TAF). So that’s come up a couple of times.

Interviewer: Right, right. Okay. Among your preceptors and you know, and there may not be enough experiences to comment fully, do any of them have preferences or do any of your co-residents or colleagues have preferences on, you know, if somebody's over a certain age or if somebody, you know, has these characteristics, do they automatically do one versus the other.

Subject 01: No, I think because the risks of all of the side effects go up with age. So, I don’t think age plays a great deal in it, you know, like specific comorbidities do, which increase in everyone in age Like, if you’re at higher risk of adverse coronary events and older, I would lean away from TAF. But these are the same people that have a higher risk for renal dysfunction.

Interviewer: And then you talked about this a little bit… when patient’s do ask you about this, you talk about risks and benefits. Although you’ve not had any patient’s switch over, what are some of the main things you talk about with patient’s who are coming in and inquiring about starting TAF or switching to TAF? What are some of the main things you go over and what role do patient preferences play?

Subject 01: Ummmm so I think I go over that they’re both probably very effective, but we have more history with TDF. I go over the different side effects and risks of each. I go over the potential cost difference, which is probably not real right now, but could very well be soon. And that we can always switch…to you know, say that we're always monitoring for things, but the monitoring might be a little different depending on which one you use. Again, I always tell people we can always switch should we see an adverse signal.

Interviewer: Okay yeah absolutely. Absolutely. I think that's oh what a lot of people are doing. You mentioned that when you're talking to patients you mentioned some have concerns about renal this function with TDF. Have any of your patients seem to heard about any of the possible, you know, adverse effects associated with TAF that you mentioned, like weight gain or things like that.Subject 01: I have not had any patient know of any of those adverse effects. And actually I didn’t know about those until I started talking more to our preceptors about it when that initial study came out about non-inferiority. And I was like oh okay, that’s strange because we’ve done this (switch) for all of the other HIV medication regimens. So, I don’t think it’s widely known at in the general community of people who might benefit from PrEP. Interviewer: Yeah for sure. And it seems like a lot of those high effects like you mentioned, you know, young people would not want to experience. And then in terms of follow-up appointments and things with patients on TDF, we have a set of labs and things that get ordered. If you were to have patient on TAF, are there any different things that you would monitor for and would you do anything differently with your follow up appointments?

Subject 01: We check for weight for everyone at every visit, so you know that’s the same. Checking lipids and A1c would be a little bit more of what I would do (for a patient on TAF). You know I don’t know if you’re at a healthy BMI and then you gave 5 pounds from that, have you like really developed diabetes, probably not. But, I would probably do like a one off… well I guess I really don’t have guidance on it really. I guess I would do a one off, rather I’d probably do it annually for those (lipid and A1c testing) for people on TAF. Whereas I don’t think that’s necessary for TDF.

Interviewer: Okay yeah, great. And then just kind of wrapping things up here, what are other experiences or thoughts that you have about TAF versus TDF or what that what that looks like, you know, in the future?

Subject 01: I think that a lot of this change, and you know this is my personal thought, is Gilead trying to make, you know, the switch over with a generic entering the marker. I think that generic HIV medications in general in the future will change things dramatically. We’ve had essentially none for any front-line agents. You know, so having at least one for PrEP would be a game changer and I think that would change the conversation a lot in the future. This would be the first front line agent really for anything HIV related. So yeah, that's the biggest change in the next couple of years I think. They're not going to reformulate like another version of tenofovir that deals with the metabolic stuff.

Interviewer: Those are most of the questions I had. Do you have any questions for us about this study or anything else?

Subject 01: No
