# Supplementary material for: Health care providers’ decision-making and early adoption of tenofovir alafenamide for HIV preexposure prophylaxis: An inductive qualitative study
Source: PLoS One. 2024 Dec 5;19(12):e0311591. doi: 10.1371/journal.pone.0311591 (PMC11620414; doi:10.1371/journal.pone.0311591)
Supplement: S1 File — (ZIP) [file pone.0311591.s001.zip › Clean transcripts/DedooseDoc_Participant 10 Transcript.docx]

I: I am going to ask you a few questions to learn what you have heard or know about using tenofovir disoproxil fumarate with emtricitabine (TDF/FTC) vs. tenofovir alafenamide fumarate with emtricitabine (TAF/FTC) for PrEP. Have you heard about using TAF/FTC vs. TDF/FTC for PrEP before today?

S: Yes.

I: And what have you heard about TAF/FTC vs TDF/FTC?

S: Um, just you known under brand name, Truvada, that you can prsescribe in a patient population being high risk or also patients who you think may benefit froma PrEP regimen.

I: Okay. What are some so What are some of the sources of your information about using TAF/FTC vs. TDF/FTC for PrEP? Some options would be Colleagues, patients, pharmaceutical reps, advertising, journal articles, continuing medical education, online information or others?

S: Mostly continuing medical education, online articles.

I: What continuing medical education?

S: Just as a part of curriculum, literature search, lectures.

I: Okay. So part of fellowship primarily?

S: Yeah

I: Have you received any guidance or feedback from medical staff at your institution regarding the use of TAF/FTC vs. TDF/FTC for PrEP?

S: Yes

I: And what have you been told by medical staff, or attendings?

S: Just on the same grounds as we discussed HIV treatment specifically, I think more into my fellowship, previously not so much of exposure or specific information on that, just knowing that it exists.

I: Okay. So walk us through your thought process when you make decisions regarding prescribing one or the other of these 2 PrEPs. So like are there some specific factors that might make you recommend TAF/FTC over TDF/FTC or TDF/FTC over TAF/FTC?

S: Um, well I haven’t done any prescription or went through the thought process of prescribing it at this point, t heoretically I think I might go lookoing more into the risk factors, their Hepatitis B status, any previous history, their comorbidities, but that’s just some questions in my mind, I haven’t actually gone through a practical exercise of prescribing

I: Are there any, aside from Hepatitis B status, are there any history or comorbidities that might make you lean one way or the other? TDF/FTC, which is Truvada, or TAF/FTC, which is Descovy?

S: Um, I can’t think of any specifically right now.

I: That’s okay. Any insurance considerations or cost considerations?

S: Again, not a topic that I’ve gone through

I: That’s okay! This question is written to be like geared for people for all different levels, so there’s like if you’ve done this before or not, so yeah. So what are some reasons... Are there any reasons or patient characteristics that would influence you to avoid a TAF-containing regimen?

S: Um, not sure

I: What about TDF?

S: That I’m not sure either.

I: That’s okay! And the next question, I think you’ve already answered, is What experiences have you had using TAF/FTC for PrEP?

S: Uh, well I have not used any

I: Okay, great, so then have you had any patient inquiries or requests for PrEP?

S: Um, no.

I: If you had a patient requesting either TAF/FTC specifically, or TDF/FTC, how would you respond to that inquiry?

S: Um, well I would do a literature review, get some more information before answering that question.

I: Fair enough. Okay, um, and then, if a patient wished to be newly prescribed on PrEP, would you tend to prescribe mostly TAF/FTC, or Descovy, or TDF/FTC, Truvada.

S: Um, I think just from experience and what’s more being in practice, Truvada, but that’s just mainly because I’ve seen that happen and it’s sort of, I think, you may want to put it as like a culture, but I would take into consideration specific risk factors and patient characteristics before reaching that decision.

I: Great. Um, alright, um, are there any questions or concerns that patients have raised about PrEP, either TDF/FTC or TAF/FTC?

S: Not to me.

I: Um, and then, the next question I think is not relevant, it’s for patients who have been switched from TDF/FTC to TAF/FTC, how has their experience been? It sounds like N/A

S: No experience, right.

I: And same for the next one, um so how, if at all, does the availability of generic TDF/FTC but not TAF/FTC influence your prescribing?

S: Um, I think I would definitely explore that option, because of a better chance of availability for affordability and access, easy access for a generic versus more brand name form. Just a general experience, medications with generic tend to be more available, easier insurance approvals.

I: Okay, great. Any other experiences or thoughts about PrEP, specifically TAF/FTC containing regimens, that you would like to discuss?

S: Um, not specifically.

I: Um, have you, just a couple of questions, we started this project before COVID and we have added a couple of questions just to capture it. As a prescriber have you noticed any influence of the pandemic on your prescribing practices for PrEP?

S: No.

I: Okay. Um, and then, from a patient perspective, have you noticed any effects on patients and their PrEP taking from the COVID pandemic?

S: I wouldn’t be able to tell.

I: Fair enough. Alright. That’s it!
