# Supplementary material for: Health care providers’ decision-making and early adoption of tenofovir alafenamide for HIV preexposure prophylaxis: An inductive qualitative study
Source: PLoS One. 2024 Dec 5;19(12):e0311591. doi: 10.1371/journal.pone.0311591 (PMC11620414; doi:10.1371/journal.pone.0311591)
Supplement: S1 File — (ZIP) [file pone.0311591.s001.zip › Clean transcripts/DedooseDoc_Participant 11 Transcript.docx]

I: I am going to start by asking you a few questions to learn what you have heard or know about using tenofovir disoproxil fumarate with emtricitabine (TDF/FTC) vs. tenofovir alafenamide fumarate with emtricitabine (TAF/FTC) for PrEP. So have you heard about using TAF/FTC vs. TDF/FTC for PrEP before today?

S: Yes

I: Okay. And what have you heard about TAF/FTC vs TDF/FTC?

S: So, I mean, so TDF is obviously the longer standing one, TAF was just approved recently this year, I believe, or the end of 2019, I’m not sure exactly, but um essentially the concerns with TDF were already exposing a large population of otherwise healthy people to potentially nephrotoxic agent, and one that could affect bone mineral density as well, as the main side effects, requires regular lab checking or renal function for that, no clear follow-up for the bone part. Um, and then TAF theoretically has lower risk of both that because it’s, you know, pro-drug, they’re both pro-drugs but it’s delivered more effectively into the cell. However, what we’ve seen a lot of is people with unwanted weight gain, not a ton, but you know like, significant to patients, on TAF. Um, given that it’s a young population for the most part whose very sensitive to body image, I’ve had very few people who want to go with TAF. Um and there’s a concern to that we’re just getting a generic TDF and is this just for a evergreening thing that Gilead is doing.

I: Okay. So what are some sources of your information about using TAF/FTC vs TDF/FTC for PrEP? Some options would be colleagues, patients, pharmaceutical reps, advertising, journal articles, continuing medical education, online information or others.

S: Probably mostly colleagues, conferences in terms of CME and journal articles, mostly, although I couldn’t tell you which one’s I’ve read recently.

I: That’s okay. Have you received any guidance or feedback from medical staff at your institution regarding the use of TAF/FTC vs TDF/FTC for PrEP?

S: Yes.

I: Okay. So then walk us through your thought process on how you make decisions regarding prescribing one or the other of these two PrEP options.

S: Um, so I think you know I go over both options with the patient, you know, and the theoretical but very low risk of renal injury on TDF, the bone mineral stuff I have not found to be especially relevant clinically for this young population, and then also mention the weight gain and metabolic abnormalities possible with TAF, so both would require lab monitoring. A nd you know I think the only time if someone has worse, you know borderline renal function, then I would, you know, lean towards TAF if they’re in that CKD II, you know, low 3A region. Otherwise most of the time people end up going with TDF after that conversation. I don’t think I’ve actually had anyone with renal function like that, because most of the 10 to 20 people I have on PrEP are all like healthy and young.

I: Okay. Um, so then you may have already answered some of these questions because they’re a little repetitive, but what are some specific factors that would make you recommend TAF FTC over TDF/FTC?

S: Mainyl just the renal function, if it’s borderline. If somebody, you know, I haven’t had this come up, but if someone you know, already had osteopenia from steroid use or something like that, that would be another consideration, but I haven’t had that come up.

I: Mmkay. And then any specific factors that would maek you recommend TDF/FTC over TAF/FTC?

S: Yeah, I think if somebody you know, is on their own concerned about weight gain or body image, if they’re prediabetic or have other markers of metabolic, you know, disarray, then I would be more likely to go with TDF.

I: Okay. How do patient preferences come into play?

S: Um, I mean, I think you know this is a treatment to prevent HIV, so they have to be fully on board since it’s a daily medication. So it’s really important to have a really upfront conversation about the plusses and minuses of both options I think. So it’s pretty important.

I: Okay. Um, any patient characteristics, like gender or medical concerns?

S: I don’t have anyone who is not male and MSM, so, and healthy, so it hasn’t come up.

I: Okay. What about any insurance or cost considerations?

S: So, that I’ve not had that come up yet. I have a pharmacist that I work with, who specifically works on PrEP and HIV who handles that. Um, but she has mentioned to me that there will be a changeover that is happening right now to the patients on TDF, um, so far it has not come to my level, but there is someone I work with who does that.

I: Okay. Um, so then what are some reasons or patient characteristics that would influence you to avoid a TAF containing regimen?

S: Um, I mean if somebody is already, you know, overweight or they already have pre-diabetes, diabetes, I would be less likely to use TAF.

I: And then any reasons or patient characteristics that would influence you to avoid a TDF containing regimen?

S: Renal disarray, bone mineral density issues.

I: Okay. Um, have you had any experiences actually using TAF/FTC for PrEP?

S: I’ve not had a single person use that, yeah.

I: Okay. Um, have you had any patient inquiries or requests for TAF/FTC for PrEP?

S: People have asked about it, um, but not requested.

I: Uh, what have they, kind of, said?

S: Is this new? Is this better? Should I be on this instead of TDF?

I: And what did you tell them?

S: They’re both effective, and then we have a talk about the plusses and minuses about both.

I: And then, what has the outcome of that been?

S: No one has switched.

I: Okay. Alright. So for patients who wish to be newly started on PrEP, do you tend to prescribe mostly TAF/FTC or TDF/FTC and why?

S: TDF – longer history, less weight gain, metabolic abnormalities. And really I haven’t seen anyone with renal adverse events. And we know how to catch it.

I: Yeah. And so for patients who are already on PrEP, to what extent, if at all, are you switching to TAF from TDF containing regimens.

S: Not, unless they request. And it hasn’t happened

I: Okay. Um, are there any questions or concerns that your patients have raised regarding TAF/FTC? Questions about effectiveness, side effects, insurance coverage, cost, pill size?

S: None of them have brought it up but so --(somewhat unintelligible)--

I: Any questions or concerns your patients have raised regarding TDF/FTC?

S: Not, I mean, like I bring up the monitoring and the monitoring more than concerns from them.

I: And then um, you said you have not had any patients who switched from TDF to TAF, right?

S: Yeah.

I: Okay, um, have you had any patients who were newly started on TAF/FTC?

S: No

I: Um, and then next one probably doesn’t apply, which is tell us about any patients who have switched from TDF/FTC to TAF then switched back… So skip that one… And then, how, if at all, does the availability of generic TDF/FTC but not TAF/FTC influence your prescribing?

S: Um, I mean, I think in general like when they’re more affordability of a safe agent I would go with the safe and affordable agent, but I know there’s complexities in coverage right now, so there are some people who might end up with their plans covering you know TAF rather than you know, a generic, but I don’t, it hasn’t come up yet, and honestly I am very blessed to have someone I work with who does deal with most of that.

I: Yes. Um, any other experiences or thoughts that you have about TAF/FTC containing regimens that you would like to discuss?

S: I mean, you know it comes up as well in people who are on TAF-containing regimens for HIV, but I’ve found it more tolerated there. I have had people get off of Biktarvy with concerns for this. I’ve had one person who switched back to a non-TAF containing regimen.

I: And that was because of weight gain?

S: Because of weight gain. Unclear if it was related to the switch, but they perceived it as such.

I: Okay. Alright. Um, for patients who are on PrEP, have you had any patients have any adverse events or negative effects from their PrEP?

S: No. I mean, initial GI upset for like a month.

I: But tolerable?

S: Tolerable. I’ve had no one stop PrEP for that. In fact, I’ve had people during COVID say that they just want to stay on it throughout because the first month is bothersome for them and they don’t want to do that again, so they just keep on it. Even though they’re not sexually active.

I: Well, that actually is a perfect segue so that was the end of our initial questions, but we added on a couple of COVID-related questions. So that was actually a perfect segue. The first of the COVID-related questions is, as a prescriber, have you noticed any effect of the COVID pandemic on your prescribing practices?

S: Um, I think our conversations are interesting, because I have noticed a couple of my patients are no longer sexually active in between the COVID pandemic, but they have continued on PrEP regardless. Which I think is interesting and I think is partially the “you never know when things will change”. I do, I’m you know very flexible with doing testing and telemedicine visits, and I don’t necessarily adhere to 3 months if somebody has been doing very well, and I don’t make them come in for screening at the sites if they don’t have any exposure. But we’ll do like a 6 monthly HIV and renal function, which can be done totally remotely.

I: And then, from a patient perspective, have you had any patients tell you about any effects of the COVID pandemic has had on their PrEP usage?

S: Um, yeah I mean I’ve had people talk to me about whether they should be taking it, because they feel honestly like they shouldn’t be sexually active due to COVID, more than anything else. Not specifically about PrEP, but about sexual activity and exposures to other human beings in an intimate manner during COVID

I: And what have you counselled them?

S: I mean, so I think that it’s interesting, because normally I’m a very big safer-sex, pro-sex person, and it’s weird to all of a sudden have to tell people that maybe, that it’s not a safe time to do that, um. So that’s been weird, I will say. And I don’t have the right answer, because I don’t want to tell people not to be sexually active, because I think that’s a part of being a human.

I: Right.

S: But I also recognize we’re in a pandemic, so I sort of tell people, “You know, the standard COVID step, if you feel sick don’t do it. You know, probably safer to have fewer partners. You know, limit to one partner if possible”, and sort of old school safe sex guidance from before the PrEP era, but now for an unrelated reason.

I: That is interesting. I hadn’t thought about it that way.

S: It’s really weird to, uh, for me, because I never would counsel people really to reduce the number of sexual partners they have this day and age. Now it’s like, okay, I mean…

I: Yeah, that makes a lot of sense. Any other thoughts about the COVID pandemic’s effects on PrEP?

S: Um, I’ve had someone ask me if COVID, if PrEP protected them, like, and I was like, I said “probably not, there was some garbage study that said maybe people on PrEP have less COVID, or people on, there was some study that said people who were on a tenofovir regimen with HIV were less likely to get COVID in Spain, but I was like “I would not use that garbage study to justify this as protection in this case”. Is clearly what I told them. Because I think they were like a biochemical researcher who had read it and they were like “Am I good?” And I was like “I don’t think this was great”. I don’t know if you read that, it’s like in Barcelona.

I: Yeah, I saw that one.

S: Not a good study

I: Not great… Alright. Well that’s all the actual questions.
