# Supplementary material for: Health care providers’ decision-making and early adoption of tenofovir alafenamide for HIV preexposure prophylaxis: An inductive qualitative study
Source: PLoS One. 2024 Dec 5;19(12):e0311591. doi: 10.1371/journal.pone.0311591 (PMC11620414; doi:10.1371/journal.pone.0311591)
Supplement: S1 File — (ZIP) [file pone.0311591.s001.zip › Clean transcripts/DedooseDoc_Participant 12 Transcript.docx]

I: I am going to ask you a few questions to learn what you have heard or know about using tenofovir disoproxil fumarate with emtricitabine (TDF/FTC) vs. tenofovir alafenamide fumarate with emtricitabine (TAF/FTC) for PrEP. Have you heard about using TAF/FTC vs. TDF/FTC for PrEP before today?

S: Um, so I think basically what I’ve heard, or what I know I should say, is I’m pretty sure that um, TAF/FTC is not currently approved, FDA approved for use, as PrEP, but obviously TDF/FTC is approved. And so, for that reason, I feel like I’ve never seen TAF and FTC prescribed as a PrEP regimen, in clinical practice. I personally don’t feel that there’s any likely reason why it wouldn’t be approved sometime in the future, but I think based on regulatory approval requirements, people favor TDF/FTC.

I: Alright. And what are some of the sources of your information about using TAF/FTC vs TDF/FTC for PrEP? So some options might be colleagues, patients, pharmaceutical reps, advertising, journal articles, continuing medical education, online information or others.

S: I would say colleagues and journal articles and I guess sort of like CME, but like for fellows it doesn’t, it's not really CME, it’s just required learning.

I: Yeah. And then have you received any formal guidance or feedback from your institution regarding TAF/FTC vs TDF/FTC for PrEP?

S: I can’t say that I’m aware of any formal recommendations or material on that, from BIDMC, yeah.

I: Alright. So then, walk us through your thought process on how you would make decisions regarding prescribing one or the other of these two PrEP regimens.

S: So, I think probably from experiental standpoint and a comfort standpoint, I would feel in general more comfortable prescribing TDF/FTC. Um, and monitoring for potential complications. I think if I were to prescribe TAF/FTC, then the things I would be more worried about would be, or considerations, you know what the patient’s renal function was, and/or whether there was a concern about you know low bone mass, or bone density. I think also emerging is potentially metabolic concerns about weight gain. So those would be kind of the things that I would consider.

I: And which of the specific factors, which if any specific factors would make you recommend TAF/FTC over TDF/FTC?

S: I think the renal and bone, if there was concern about renal and bone toxicity, I think that would probably favor the TAF/FTC regimen.

I: And then any specific factors that would make you favor TDF/FTC over TAF/FTC?

S: Basically I think if there’s no reason why you couldn’t get, I would just look at it sort of like that. If there no specific reasons why the patient couldn’t get TDF/FTC, then I would continue with that. I think the other thing too would be that cost I think would be a concern too because TDF/FTC is generic, so if there’s an issue of cost or coverage, then that might be a preferable option for the patient, if they are paying out of pocket particularly.

I: Great. Are there any reasons or patient characteristics that would influence you to avoid a TAF containing regimen?

S: Um, cost? A nd then I can’t remember what the GFR cut off is for TAF, but that would be something I would check and make sure of, and I don’t think there’s any... There might be recommendations around liver disease as well. I would double check that, I’m not sure. But sort of basically medical comorbidities that would exclude them.

I: Mmhmm, sure. And then, any reasons or patient characteristics that would influence you to avoid a TDF containing regimen?

S: I guess, this is like maybe more meta level, but like I think it’s always worse re-assessing whether the patient is, would benefit from PrEP. I think if they’ve been on PrEP for a long time and perhaps their, you know sexual practices have changed, or you don’t think the risk/benefit is their favor, then I think , you know, the question about whether they need to be on it at all is worth reconsidering. Although the toxicities are few, they’re not zero. And then, I think the same thing. I think if your medical comorbidities might play a factor in you deciding that was an appropriate therapy.

I: Mmmhmm. And for TDF what would those medical comorbidities be for you?

S: So again, I think it would probably center around renal function, bone mass.

I: Yeah.

S: I guess age, to some extent, would be related to those two things. So maybe an older person where, even if they have had reasonable renal function and okay bone mass, you might consider alternate, like the TAF regimen, just for concern about long-term side effects if the patient was going to be on PrEP. Typically, obviously we’re not prescribing it to seventy or eighty year old people, but I mean, it’s certainly possible.

I: Mmhmm. Okay, have you had any experiences using TAF/FTC for PrEP?

S: Uh, no.

I: Um, do you have any patients on your panel who are on TAF/FTC for PrEP?

S: Not for PrEP, no.

I: Okay. Um, have you had any patient inquiries or requests for TAF/FTC PrEP?

S: I don’t think so. I feel like I want to say perhaps somebody’s asked about it, as a question, because they may have seen something about it on television, or maybe in some sort of social media or something, but um, yeah. I don’t know if I’ve even mistaking that with other HIV patients who have asked about different things. But generally I would probably say no.

I: If you did have someone, a patient, who asked you, or requested TAF/FTC for PrEP, how would you respond?

S: I think I’d have to. Up front I’d probably, unless they had some specific indication whereby they couldn’t, or I felt it would be risky to put them on TDF/FTC. I don’t think I would recommend the TAF/FTC necessarily. I have to double check whether again, maybe it is already approved. Probably is based on the questions that you’re asking me, but yeah I don’t know. I must say, I don’t know what the evidence is about benefit over, so I would have, I would probably recommend that the patient be on TDF/FTC and then read more about it and readdress it with them.

I: Makes sense. Okay, so um, are there any reasons that you either have not or would not start a patient on TAF?

S: I think probably familiarity with the updates in the literature around the benefits, would probably be my main reservation. And I think the, like paucity of really down-sides to TDF/FTC in terms of toxicity, tolerability, cost.

I: Okay. And then, what are some potential risks and benefits that you weigh when deciding whether to choose TAF/FTC vs TDF/FTC?

S: I think now probably from the standpoint of Truvada being generic, that cost would be a factor. And then also, you know, and again I’m not up to date on the exact risk/benefit trials, or the... I think there... there’s definitely non-inferiority trials that were positive ,but I don’t know about what the outcomes were in terms of the toxicities. I think in general, from the HIV literature, obviously there are some modest benefits to TAF/FTC, but I perhaps in a PrEP patient wouldn’t necessarily sway me dramatically, especially because of the cost differential, to just giving everybody Descovy.

I: Um, in the HIV literature, what are those modest benefits?

S: So renal and bone toxicity, so reported lower renal toxicity, lower bone toxicity in the TAF/FTC people, or group.

I: Okay. So then, for patients who wish to be newly started on PrEP. Would you tend to prescribe mostly TAF/FTC or TDF/FTC and why?

S: Uh, so yeah, again I would probably at this point still preferentially prescribe TDF/FTC, for sort of the maybe lack of familiarity with the data on TAF/FTC plus or minus the cost issue.

I: Okay. Um, for patients who are already on PrEP, to what extent, if at all, are you switching patients from TAF to TDF. Sorry to TAF from TDF containing regimens.

S: I haven’t, yeah I haven’t seen, I personally haven’t done that. I haven’t seen any of the attendings I work with do that, but I think it would, I think part of that decision would be also similarly why we see people every three months and check their labs. I f there was a dramatic change in their renal function, or you know, you’re meeting with a patient who has been on PrEP for a decade, or long periods of time, then I think it’s worth considering whether they may benefit from a switch, and to what extent they may see any benefit, but yeah generally I haven’t made any of those switches

I: Okay. Have you had any patients raise any questions or concerns regarding TAF/FTC?

S: Um, no.

I: Okay. Have you had any patients raise questions or concerns regarding TDF/FTC?

S: Um yeah, I think generally people are, I don’t know, I mean concerns on a new start, I think with all patients is generally around toxicity and efficacy. But that’s sort of part of the general counselling for someone who’s starting on PrEP regardless?

I: Any particular toxicity concerns that patients have raised, or just kind of general questions?

S: Yeah, I can’t think of any specific patient who would have described a specific toxicity, that was sort of outside of just their concern about the medication impacting their own personal health. I suppose a few people may have asked about medication interactions, but aside from that, not too many. Mind you, I think predominantly all the people that I’ve seen started on PrEP, or I’ve started on PrEP, have been younger, with very few medical problems or no medical problems.

I: Any patient questions or concerns about insurance coverage, out of pocket costs, or pill size?

S: Cost comes up quite often, and insurance coverage comes up quite often.

I: Um, have, for, if applicable, have you had any patients who have switched from TDF/FTC to TAF/FTC?

S: No.

I: Have ypu had any patients who are newly started on TAF/FTC?

S: For PrEP, no.

I: Okay. Um, and then I assume then so that the next question is about patients who have switched from TDF/FTC to TAF then switched back. I’m assuming not applicable.

S: Not applicable.

I: Okay. How, if at all, does the availability of generic TDF/FTC but not TAF/FTC influence your prescribing?

S: I think it influences it a fair amount.

I: In what way?

S: I think um, I don’t think that, I am not a hundred percent up to date, but I think in my mind the reported potential benefits vs the cost differential for the vast majority of benefits would not be an economical, you know, it wouldn’t sort of add up for me.

I: For TAF you mean?

S: For TAF, being so much more expensive. Although again, I don’t know the specific cost, but if you have a medication which is safe and well-tolerated, and equivalent, say to an alternative that’s much more expensive, I don’t see any great need to switch. In fact, I think it probably influences me the other way, to say “I think maybe that’s a huge bonus of TDF/FTC”.

I: Mmhmm. Have you had any patients on any PrEP regimens who have had any significant adverse events or negative effects?

S: No.

I: Alright, any other experiences or thoughts you have about TAF/FTC containing regimens that you would like to discuss?

S: I don’t think so.

I: Alright, that was the end of our, sort of initial questions. We’ve tacked on a couple of COVID questions, because why not. So the first COVID question, is as a prescriber, have you noticed any influence that the COVID pandemic has had on your prescribing practices for PrEP?

S: That’s a good question. I don’t think, I don’t think so. Perhaps early on, there were a few PrEP patients that did not have the labs done on time, or sort of like, their routine safety labs done as normally would do them. And I think that’s still probably okay, because they were done, just not as easily and as timely as they normally would have because patients had their appointments cancelled and I think a lot of the PrEP patients, because they’re well otherwise, were not high on the priority list to be re-booked. So I would say that’s probably the only impact I’ve seen. But other than that, like in terms of starting people on PrEP or things like that, certainly many people have started on PrEP, mostly in the setting of NPEP to PrEP, during the pandemic, but that hasn’t been interfered with, and I don't feel like there’s less people, sort of qualitatively, that we’re starting on PrEP over the last 6 months or so.

I: Alright, and then, from a patient perspective, have you had any patients report any impacts that the COVID pandemic has had on their uptake or usage of PrEP?

S: I certainly have seen some patients stop because, you know their sexual practices have changed during the course of the pandemic, and so some people don’t feel like they need to continue on it. And certainly will, once they become more sexually active, but I think a lot of people have changed their, sort of personal behaviors. They’re not going out as much, they’re not meeting people, things like that.

I: Okay. Any other thoughts about the COVID pandemic and its effects on PrEP?

S: Hmm. Yeah, I guess, I don’t know how things will... I suppose it’s been kind of minimal impact, but it’s hard to know how many people perhaps who could have benefited from PrEP who would have seen you for some other reason in person, where it’s kind of easier to have a more robust conversation, would have been started on PrEP. So I wonder if the overall number, although qualitatively doesn’t feel different, the overall number of new starts on PrEP over the course of the pandemic as compared to prior is lower, I suspect probably is. So, you wonder how many HIV infections could be prevented in that setting.

I: Great. That’s the end of the questions.
