# Supplementary material for: Health care providers’ decision-making and early adoption of tenofovir alafenamide for HIV preexposure prophylaxis: An inductive qualitative study
Source: PLoS One. 2024 Dec 5;19(12):e0311591. doi: 10.1371/journal.pone.0311591 (PMC11620414; doi:10.1371/journal.pone.0311591)
Supplement: S1 File — (ZIP) [file pone.0311591.s001.zip › Clean transcripts/DedooseDoc_Participant 13 Transcript.docx]

I: I am going to ask you a few questions to learn what you have heard or know about using tenofovir disoproxil fumarate with emtricitabine (TDF/FTC) vs. tenofovir alafenamide fumarate with emtricitabine (TAF/FTC) for PrEP. Have you heard about using TAF/FTC vs. TDF/FTC for PrEP before today?

S: I’m sorry, you’re breaking up just a wee bit as you’re speaking. I got most of what you’re saying, but I didn’t actually get the specifics of the question. And if you don’t mind me using the words Descovy and Truvada it’s going to be a lot easier on my tongue than TDF/FTC and TAF,

I: That’s totally fine. I’ll try to fix my microphone to make it sound a little better.

S: It just, what it’s doing is it’s getting a little bit like, a typical sort of microphone using computer, which I may be, I don’t know how my sound is on your end.

I: Actually totally fine.

S: Okay, let’s ask the question again, and see if.

I: Sure, so have you heard about using TAF/FTC vs TDF/FTC for PrEP before today?

S: Yes, of course.

I: And what have you heard about TAF/FTC vs TDF/FTC?

S: That’s a big open-ended question.

I: Yes it is.

S: Well, both have been well studied for both, TDF has been around much longer and we have significant longer period of use in and TAF is relatively new comparatively, for the use of PrEP. And both have in clinical trials have shown to be effective. How much more do you want me to keep talking?

S: I have some more specific, kind of driving down questions, so we can, that’s okay, we can kind of keep going. So what are some sources of your information about using TAF/FTC vs TDF/FTC for PrEP? Some options might be colleagues, patients, pharmaceutical reps, advertising, journal articles, continuing medical education, online information, or anything else?

S: Mostly through the review of the actual studies that have been done. Obviously expert opinion, talking to colleagues, it’s hard to avoid seeing it on commercials, but I don’t use commercials as a management tool, but I do see that, so that’s pretty much the.

I: Great. And have you received any formal guidance or feedback from your institution regarding the use of TAF/FTC vs TDF/FTC for PrEP?

S: Um, indirectly, I think that there now is a general preference for the use of TDF/FTC because it’s available generically, but it is not a formal mandate in that regard.

I: Got it. So then, walk us through your thought process on how you’d make decisions regarding prescribing one or the other of these two PrEP regimens.

S: For me, right now, my general... Did I lose you?

I: I’m still here.

S: Sorry about that, what happened was is that my, I had my medical record opened, and it closed down and then the screen popped up, so... My general thing is that for the most part I’m using TDF/FTC as first line for the vast majority of my patients. And um, the one’s I’m not using it on are more people who have contraindications or are higher risk. Which I can go into if you want? Or you’re going to sort of...?

I: Yeah, I mean, my next question is what specific factors would make you recommend TAF/FTC over TDF/FTC? so there you go.

S: If patients have underlying renal disease, if patients have underlying osteoporosis, if patients have had prior experience with TDF and have had some sort of adverse reaction that they’ve experienced with it, such as GI side effects in some patients. Um, trying to think if I can sort of hone it down a little bit more for you. Um, my general sense is that the longer experience with TDF, the now slightly lower cost of TDF, generally continues to make me want to use that drug still first-line in the vast majority of my patients.

I: Great. And that pretty much answers the second question, the next question, what factors would make you prescribe TDF/FTC over TAF/FTC, I don’t know if you have any other things you would want to add there.

S: I think that the unknown longer term side effects of TAF that we’re still exploring. I think that the weight gain issue has been somewhat interesting, and how much that will play out in the long run. The potentially better lipid lowering effects of TDF make me want to think about using that drug more, and I think that’s pretty much it. And the cost of TAF is higher.

I: Great. Um, do patient preferences or patient characteristics come into play?

S: It’s hard not to. I think we always have to, you know, accept that. Patients are going to have preferences. They’re going to be influenced by what they hear from other friends, what they hear from the media, what they hear from advertising, so yeah. Patient preferences always come into play, but in general, that’s not too difficult to work around for me.

I: Okay. Then, what are some reasons, and you may have already answered some of these questions, they’re a little bit repetitive, so what are some reasons or patient characteristics that would influence you to avoid a TAF containing regimen, if anything additional that you would want to add?

S: Patient characteristics? Beyond what I’ve already said, I mean obviously if they. I didn’t say if they have other multiple risk factors for renal disease I might not use it, I might, and older patient with diabetes, hypertension, a modest reduction of renal function, I might choose to use TAF in that patient. I hate to say this, difficulty swallowing pills. TDF is large, some patients won’t take pills because they’re very big, and TAF is smaller. So that can make a very big difference, and of course it’s all about adherence, it doesn’t matter which one I give them if they don’t take it. That would be another factor that I would consider. Um, and then of course we have the insurance companies who are playing their role, right? So the insurance companies have preferences now. And they didn’t, earlier in the year, but the insurance companies are also now determining whether people get one drug or the other, and now there is this new, for many patients who want to use TAF, they need a prior authorization.

I: Okay.

S: It’s a hoop. It’s a hoop to jump through.

I: Yes, yes it is. Those are painful. So then what experiences have you had using TAF/FTC for PrEP?

S: I’m not sure what you mean by that, I’ve been using it for quite a while, in many many many many patients, and generally my experiences have been good. Most patients have been good, most patients have been taking the drug quite regularly, and uh, I have explored, maybe you’re going to be talking about this later , I’ve explored alternative ways beyond just daily PrEP with patients, so although most of my patients are taking it on a daily basis, some people are using it on an “as needed” basis, vacation PrEP – very few people are using it in the French model of “on demand” in sort of like the IPERGAY study that’s a very rare way in which my patients are using this drug, I’m not necessarily a huge fan of it. Um, I don’t know if that answers your question, where you were directing it.

I: It’s a pretty open-ended question, getting a little bit more closed, do you have any patients currently on your panel who are on TAF/FTC for PrEP?

S: Yes.

I: Um, and what factors influence your decision to prescribe those patients a TAF-containing regimen?

S: Smaller pill size, underlying medical condition, patient desire to be on it, insurance coverage issues. That’s pretty much it.

I: So then, describe your decision-making when you’ve selected TAF/FTC for patients who are newly started on PrEP?

S: My decision-making? Usually it’s because I’ve already seen them as a high risk for complications or a higher risk for complications from TDF. So for me it’s TDF exclusionary exclusion, leading... that’s generally my decision making. You need to be on.. First decide, “Do you want to be and need to be on PrEP? How do you want to use PrEP?” Dealing with the safer sex issues in general, then TDF is my preference, and then “why can’t you be on TDF”, then maybe go on TAF.

I: Okay. And have you had any patients who have swiched from TDF to TAF?

S: Yes

I: And what was the decision making like for those patients?

S: Boy that varies, it depends on what month you were talking about. I think early on there was a lot of hype, and when the TDF studies got published or announced and there were people who were looking to be on the newest agent. So there was some patient pressure to switch. At that point the price was the same, and it wasn’t clear, I think we knew that TDF was going generic at some point, but knowing the FDA and knowing pharmaceutical companies, we were far enough away from that time period that I wouldn’t necessarily have bet ym house on the fact that it actually would have happened this fall. So there was that initial patient pressure, I wasn’t particularly pushed myself to sort of say, I was not a big proponent of switching. The initial switches were all people I thought who had borderline renal function, compromised GFRs, other risk factors, people who maybe I was not all that comfortable where they are, or even again patients with bone loss or high risk for bone loss. And so patients who maybe have had a lot of steroid use, I may have said “Let’s not just give you one more agent that, let’s get you off this agent, maybe we can regain a little of that bone”

I: Makes sense. Um, you’ve already sort of answered this question, but for patients who wish to be newly started on PrEP, do you tend to prescribe mostly TAF or TDF, and why?

S: TDF – cost, experience, well-known side effects, the side effects are minimal. I mean, having talked about renal or bone issues, they’re really not clinically significant in the vast majority of patients. And so that was it. Experience. Yeah, that’s it.

I: Okay. Are there any questions or concerns that your patient has raised regarding TAF/FTC?

S: Patients? Just “will it be covered? Will the insurance pay for it?”

I: Got it. And what about TDF/FTC - any questions or concerns from patients about TDF/FTC?

S: I think that the patients seem to think that the renal... my experience is that a small percentage of people seem to be afraid of the kidney toxicity. Or sometimes they come in and say “oh I hear it messes up my liver”, but you know. So there is this lore out there about what it does or what it doesn’t do. I think patients, there are patients who don’t like to take pills period, so this idea of “is my risk really high enough to warrant the side effects?” I think that there are some people who are worried about the GI side effects that might happen early on with the drug, because they have experience with friends who might have started with the drug who had it. So.

I: Okay. Great. Um, and then for patients who have been switched from TDF/FTC to TAF/FTC, how has their experience been?

S: Um, I’ve not had anybody who’s fed back to me that they’re unhappy with it. I’ve had a few people gain a little bit of weight, and a few people have said “is that due to the drug?” and we’ve switched a couple of people back, but it’s a small, probably not even a handful of people yet, so the bigger experience has been that they have to jump through hoops in terms of getting it, continuity can sometimes be an issue because of the prior authorization. They ask me for it when they have 4 pills left of their TDF, and I can’t get them the TAF in 3 days.

I: Right, makes sense. How has the experience been for patients who are newly started on TAF/FTC?

S: It’s been fine. I’ve had very few.. I’m trying to think if anybody’s even called me back and said or messaged me that they’re having tolerability issues. People seem to like it. I don’t... there were, I think my experience is that there were maybe fewer GI side effects from the drug? In terms of my anecdotal, kind of response, but I have way more people on TDF than TAF, and so it just may not be that I’ve had enough patients on TAF to be able to pick up whether there is some GI intolerability.

I: Any particularly positive experiences on either regimen?

S: I think the vast majority of people are particularly happy to feel protected. And I think that the experiences are very similar for people.

I: Okay, great. Um, so you mentioned that you’ve had some patients who have switched from TDF/FTC to TAF/FTC then switched back. Tell us more about that.

S: Um, well the patients I was referring to earlier were a couple of patients who had gained a significant amount of weight, I’m talking in the 10 lb or more range, and whether that was related to the drug or other lifestyle issues it is hard to know, some of it has to do with the fact that we’ve got a pandemic going on, and people are sitting at home and less active, so if I switched them in February to TAF and they are now talking to me in October, November and they’ve gained weight, I don’t know whether it’s one or the other, but that has been their experience. I don’t have enough in my head experience to say “oh did they lose that weight when they switched back?”

I: Mmmhmm. Have, aside from the weight, any other reasons why people have switched to TAF then switched back to TDF?

S: Insurance coverage.

I: Makes sense. Have you had any patients who have discontinued PrEP altogether?

S: Yes

I: And what were the reasons there?

S: Usually a change in the, usually, the change in the perception of their risk for HIV acquisition.

I: Alright. And then, how if at all, does the availability of generic TDF/FTC but not TAF/FTC influence your prescribing?

S: Well I, you know, it’s less expensive to the healthcare system. Whether, so it has a great influence. It’s also confounded the influence, confounded the issue, because Gilead had been quite good about covering every penny that the insurance company didn’t pick up, including the deductible and the copays. Although Teva has a co-pay assistance program, it’s slightly less robust, and the way that they’re managing it with their $600 a month coverage may not cover all of the deductible issues, and we’re still sort of figuring out how to get those patients. I think this is too new, but we’re sort of figuring “Okay, how are we going to keep those patients on generic TDF”. I think that we will, because there are lots of, there are other programs to keep people on their, on the drug, but that that is a hot issue as we’re sort of dealing with the first couple of weeks of November after the availability of the generic. And of course, it’s an automatic switch for patients. It’s resulted in a lot of phone calls about “what is this? Why am I on this? Is it just as good as truvada?” That kind of stuff. And when you have as many patients on it as I do, it’s not inconsequential.

I: Makes sense. Any other experiences or thoughts you have about TAF/FTC vs TDF/FTC that you would like to discuss?

S: Nope. I think I’ve covered most of what I.

I: Great, so that’s the end of what we had initially, when we first made this study back in February or so. We’ve added a couple of questions about the COVID pandemic, since we’re already interviewing people about PrEP. Um, so the first question is “As a prescriber, have you noticed any impact of the COVID pandemic on your prescribing practices for PrEP?”

S: Well, I think that there are many of my patients who have come off of PrEP. And so many of my patients have basically in quarantining and self-isolation have reduced their risk for HIV acquisition, and have come off of PrEP. But I don’t think it has changed any of my TDF vs TAF kind of issues. It’s really a matter of whether they need to be on PrEP right now or not. I’m always surprised at the number of patients who have stayed on it. I have patients who are nine months on PrEP and have not had one single sexual encounter in that nine month period and still continue to take the drug. So, in fact I’m okay with that. I’m assuming in the back of their mind they’re saying “I just never know whether tomorrow I might decide to have a sexual encounter that might put me at risk” and I'm perfectly fine with that. And others feel a little bit more under control, and so they feel like they could start their drug back up and kind of deal with the need to deal, and ramp it back up before they need to be, before they’re at risk again.

I: So this leads into the second question, which is from a patient perspective, have you noticed any impact of the COVID pandemic on patient acquisition and PrEP practices?

S: Well, I would say that my experience is that early on, people shut down their sexual encounters. I will tell you about mid-summer, as the numbers in Boston dropped quite a bit, we had, we were obviously as we all know, were one of the safer spots for COVID acquisition come July and August, compared to lots of the other country. I saw an increase in the number of people that were becoming sexually active again outside of their primary relationships or their household partners. And we saw an increase in people wanting to go back on PrEP if they had been off of it, an increase in people finally coming back into care, after maybe saying “I haven’t been sexually active so that’s why I wasn’t there for the last, for my 3 month follow-up". And uh, and increase in STDs that we saw, come the end of the summer, it started to kind of go up where I just wasn’t seeing anything in April and May and June.

I: Makes sense. Any other thoughts about the COVID pandemic and it’s effects on PrEP?

S: No, I mean, I do have a little worry that some people might restart on their own, I have this fear that there’s going to be that one or two people somewhere along the line who is going to have sex on Saturday, having been off their PrEP for months and then realize “oops, you know what, I really should go back on my PrEP” you know because they didn’t use a condom, and then that they will have been infected and it’s not something that’s happened to me often, but it has happened. I have had people who have had that sort of “oops” moment and then just restarted on their own without coming back to me, and so I do worry a little bit about that and the development of resistance, but that’s just a worry. And I also worry that people may not perceive that we’re available. That “how do I get PrEP, you’re closed.” You know this idea that we’re closed, or that it’s difficult to come in, or that we’re not doing screening during PrEP, you know, whatever the patient perception, which is not the truth, but I do worry that people may not choose to access PrEP during the pandemic. Or the fear of health centers. The fear of coming into our office even though we’re trying very hard not to do COVID-care within our offices. And patients are doing self-swabs, so I do worry a little bit about that.

I: Great. Alright, so that’s all my questions.
