# Supplementary material for: Health care providers’ decision-making and early adoption of tenofovir alafenamide for HIV preexposure prophylaxis: An inductive qualitative study
Source: PLoS One. 2024 Dec 5;19(12):e0311591. doi: 10.1371/journal.pone.0311591 (PMC11620414; doi:10.1371/journal.pone.0311591)
Supplement: S1 File — (ZIP) [file pone.0311591.s001.zip › Clean transcripts/DedooseDoc_Participant 14 transcript.docx]

I: I am going to ask you a few questions to learn what you have heard or know about using tenofovir disoproxil fumarate with emtricitabine (TDF/FTC) vs. tenofovir alafenamide fumarate with emtricitabine (TAF/FTC). Have you heard about using TAF/FTC vs. TDF/FTC for PrEP before today?

S: I guess I had a little bit. Hopefully this isn’t too embarassing, but I feel like the TAF wasn’t approved for women until very recently. I don’t even know if it has been. I think it was approved for women recently. But I think they should have, in the otherwise they have equal efficacy, as far as I know

I: Okay. Alright and so then what are some sources of your information about using TAF/FTC vs using TDF/FTC for PrEP? Some possible options might be colleagues, patients, pharmaceutical reps, advertising, journal articles, CME, online education or others?

S: Yeah, really colleagues and Twitter.

I: Good

S: Probably.

I: Excellent. And then, have you received any guidance or feedback from medical staff at your institution regarding the use of TAF/FTC vs TDF/FTC for PrEP?

S: I think so.

I: In formal settings or just like informal, like precepting?

S: I don’t think the subject’s come up

I: Okay. Then, walk us through your thought process on how you would make decisions regarding prescribing one or the other of these two PrEP regimens.

S: Um, I guess it depends if. I think the side effect profile with TAF is a little bit better, especially with renal and bone disease, so if someone was younger, I think that, younger or older, I’d probably lean towards doing TAF-based, instead of TDF. I guess the weight gain maybe is, may be a factor, I think less so than the other side effects.

I: Okay so what are some, and you, some of these questions get a little bit repetitive, so if you feel like you’ve already answered it you can just say so, but what are some specific factors that might make you recommend TAF/FTC over TDF/FTC?

S: I think the renal insufficiency is one, and I think someone who may be at higher risk for degenerate bone disease would be another. Um, like I said I think the, those are the main ones. I don’t know if cost is different. Truvada I think is generic now.

I: Mmmhmm

S: So maybe it costs more? I don’t actually know, what’s the real cost to the patient difference.

I: Okay. Yeah, so then would there be any specific factors that would make you recommend TDF over TAF?

S: I guess the cost, the potentially cost. I don’t know if that’s real or not. The weight gain, I don’t know if that’s, how significant that is I guess, I’d have to look. I think it’s moreso if it’s with dolutegravir, I’m not sure if the TAF alone is that much more. I don’t know.

I: Yeah, no, totally fair. You should not feel self-conscious about this at all, I’ve been interviewing everyone from internal medicine residents, all the way up to like attendings. So it’s all... don’t worry. Um, okay, so what are some, have you had any experiences using TAF/FTC for PrEP?

S: No.

I: So then, do you have any patients on your panel who are on TAF/FTC for PrEP?

S: No

I: Um, have you had any patient inquiries or requests for TAF/FTC for PrEP?

S: No

I: Um, if you did have an inquiry, if a patient came to you and asked you about TAF/FTC for PrEP, what would you tell them?

S: Are they already on TDF, or just they’re not on anything?

I: I guess if you had a patient who was on TDF, and they came to you and asked you about TAF for PrEP, what would you tell them?

S: Yeah, I think I would tell them that you know, it probably is equal efficacy, you know you might get a more favorable side effect profile in regards to most things with TAF, so I think it’d be certainly reasonable for them, if they wanted to switch I think it would certainly be reasonable.

I: And If you had a patient who had not ever been on PrEP before, and they were asking you about TAF/FTC, what would you recommend?

S: Yeah, I would recommend it if they weren’t on anything and wanted PrEP, that could be a reasonable choice. I don’t think I’d have them take TDF over TAF.

I: For patients who wish to be newly started on PrEP, would you tend to prescribe one or the other, TAF/FTC or TDF/FTC and why would you lean towards the one you would lean towards.

S: Yeah, I think, I think I’d lean towards TAF given the more favorable side effect profile. I guess the only caveat is I’m not totally sure what the cost factor is, and if that would matter.

I: Okay. I’m guessing that this, that I already have the answer to this one, but for patients on PrEP, to what extent, if at all, are you switching patients from TDF to TAF regimens?

S: I don’t have anybody on either.

I: That’s okay. Have you had any patients raise any questions or concerns regarding TAF/FTC or TDF/FTC?

S: Not in the context of PrEP, no.

I: Okay. And then, I’m guessing I already know the answer to this one as well, but for patients who have been switched from TDF/FTC to TAF/FTC, how has their experience been?

S: Yeah... I don’t know.

I: Um, and then how does the availability of generic TDF/FTC but not TAF/FTC influence your prescribing?

S: Yeah, I mean I guess it goes back to whether cost is a factor for the patient or not.

I: If TAF/FTC did cost more, would you still lean towards recommending TAF/FTC? Or would it really just like an individualized patient...

S: Yeah, I think it would be a little bit of shared decision making and if there’s any like, you know, stronger reason why they couldn’t be on TDF.

I: Right. And those would be...

S: If they had CKD or again had like risk factors for demineralization.

I: Okay. Um, have you had, are there any other thoughts or experiences you have regarding TAF/FTC containing regimens that you would like to discuss?

S: Um, I don’t think so.

I: Okay. So that is the end of the initial part of the interview, we have tacked on a couple of questions about COVID, just because we started this, I don’t know, way back in the Spring, and uh why not. So the first question, is as a prescriber, have you noticed any effects of the COVID pandemic has had on your prescribing practices for PrEP?

S: Yeah, no I think I just didn’t have a practice pattern before, so I guess it’s kinda..

I: That’s fair. Um, and then have you had any patients tell you about any impacts that the COVID pandemic has had on their, either uptake of PrEP or use of PrEP or risk factors, or anything else regarding PrEP?

S: No I think I’ve had patients that have remarked that they’re sort of less sexually active than they would have been due to the pandemic, but not anything specific to PrEP I guess.

I: Alright. Any other thoughts about PrEP and the COVID pandemic?

S: No.
