# Supplementary material for: Health care providers’ decision-making and early adoption of tenofovir alafenamide for HIV preexposure prophylaxis: An inductive qualitative study
Source: PLoS One. 2024 Dec 5;19(12):e0311591. doi: 10.1371/journal.pone.0311591 (PMC11620414; doi:10.1371/journal.pone.0311591)
Supplement: S1 File — (ZIP) [file pone.0311591.s001.zip › Clean transcripts/DedooseDoc_Participant 15 Transcript.docx]

I: I am going to ask you a few questions to learn what you have heard or know about using tenofovir disoproxil fumarate with emtricitabine (hereafter TDF/FTC) vs. tenofovir alafenamide fumarate with emtricitabine (hereafter TAF/FTC) for PrEP. Have you heard about using TAF/FTC vs. TDF/FTC for PrEP before today?

S: Yes

I: And what have you heard about TAF vs TDF?

S: Um, so TAF is obviously newer. It is not indicated for all the populations that might need pre-exposure prophylaxis. And you know, it confers a very modest kind of temporary benefit with respect to kidney function and bone density relative to TDF.

I: Okay. And what are some of the sources of information about TAF vs TDF, so some examples might be colleagues, patients, pharmaceutical reps, advertising, journal articles, continuing medical education, online information or others.

S: Yeah, like all of the above, right? I think because I work at Fenway, we get a lot of inservice. So we have had speakers do that.

I: Alright. Which is pretty much a direct answer to my next question, which is have you received any guidance or feedback from medical staff or your institution regarding the use of TAF/FTC vs TDF/FTC for PrEP?

S: Um, have I gotten what? Say it again?

I: Um, any direct guidance or feedback from medical staff at your institution?

S: Oh you know, a lot of questions. I’m the medical director, so I’m usually the one giving the guidance and the feedback. But we get a lot of questions, especially now that um, you know that there’s an additional drug available.

I: Are there formal training sessions? Or have there been any sort of lectures, or anything else in your institution?

S: Yes, and we have a formal written policy on pre-exposure prophylaxis.

I: Alright. So then walk us through your thought process on how you would make decisions regarding prescribing one or the other of these two PrEP options.

S: I would prescribe Truvada to everyone, other than people with a history of kidney disease, or osteoporosis or osteopenia. And um, an exception would be if a patient specifically requested Descovy, and I was unable to convince them to try Truvada.

I: So then what specific factors would make you recommend TAF over TDF?

S: So, if the patient had kidney disease, if the patient had osteopenia or osteoporosis, if the patient requested it specifically, or if for whatever reason which is unlikely, but if for whatever reason there was a relative, if there were relatively less kind of insurance hassles or coverage issues with Descovy. Those are all factors that would help me choose that over Truvada.

I: Okay then yeah, so the same question, so what would make you choose TDF/FTC over TAF/FTC?

S: Um, obviously the indications, so in some patients clearly TDF is the only option, um, and then you know, you’re, for your prototype patient, who doesn’t have the problems I mentioned already, that would be my go-to drug. And then again like the insurance coverage issue. This is especially important now that there’s a generic for Truvada.

I: Great, um, alright. And then what experiences have you had with using TAF/FTC for PrEP?

S: I have, you know, plenty, and it’s fine.

I: Do you have any patients on your panel who are on TAF/FTC for PrEP?

S: Yeah.

I: Okay. Um, so then what factors influenced your decision to prescribe those patients a TAF-containing regimen for PrEP?

S: Uh, again, patient choice, patient comorbidities, um, I don’t have any who are on it now because of the indication situation, so I don’t have any cisgender women or trans men on PrEP right now. And then ease of insurance coverage.

I: For, have you had patients who are newly started on PrEP who you started on Desco, or on TAF/FTC?

S: Say again?

I: Have you started TAF/FTC in patients as their first PrEP regimen?

S: Um, probably, yeah. Probably on less than a handful of people.

I: Okay, any differences in the decision-making for that vs for people who your switching?

S: No, I mean, you still have to counsel them about side effects, and how to handle them.

I: And then, um for patients who have switched from TAF/FTC to TDF.. Or sorry, from TDF/FTC to TAF/FTC, what was the decision-making there?

S: The only people that I’ve switched to, that I’ve made that switch for, are people whose creatinine has become abnormal, or whose urine protein test has become abnormal. Or there have been a few patients who have just heard that it’s safer and want to switch, and um, you know, I was unable to convince to stay on Truvada.

I: Alright. Um, and then what are some potential risks, and potential benefits that you would way when you’re considering prescribing TAF vs a TDF-containing regimen?

S: Say that one more time?

I: What are some potential benefits and potential risks that you might weigh when deciding to prescribe TAF vs a TDF-containing regimen?

S: Um, I think TAF is associated with weight gain, which a lot of my PrEP patients would like to avoid. Um, and then there could be a financial cost. Not so common in Boston, but oftentimes there, in other places there, and we have patients who travel, there could be pharmacies that don’t stock, they don’t stock TAF. And so, that could be potentially a problem for patients.

I: So for patients who wish to be newly started on PrEP, do you tend to prescribe mostly TAF/FTC or TDF/FTC and why?

S: TDF. I mean, I think it’s clearly the first line drug, and when you take into account its effectiveness, its range of indications, and its cost.

I: Alright, and then, um, what are some questions or concerns that your patients have raised regarding TAF/FTC?

S: I don’t think they’ve raised any other othan the coverage or cost issue.

I: Right. Any questions or concerns that patients have raised regarding TDF/FTC?

S: Yes, they get all those ridiculous Facebook ads telling them to sue Gilead because of TDF toxicities.

I: Has anyone asked any particular questions about the relative effectiveness, or relative side-effects between the two?

S: Yes.

I: More specifically, what questions?

S: People specifically want to know if Truvada is going to hurt their kidneys or their bones.

I: Any patients have any issues with pill size or any characteristics of the drug itself?

S: Not that I’ve heard of.

I: Okay. Um, for patients who have been switched from TDF to TAF/FTC, how has their experience been?

S: It’s been fine.

I: Any particularly positive experiences, or adverse events, or particularly negative experiences?

S: No

I: And then, kind of the same question, but for people who are newly started on TAF/FTC, how has their experience been?

S: It’s been fine. These are relatively very safe medications.

I: Yeah. And then, if applicable, tell us about any patients who have switched from TDF/FTC to TAF/FTC and then switched back?

S: I don’t know of anybody who has done that.

I: Fair enough. Have you had any patients who have discontinued PrEP altogether in the setting of making these switches, like been unable to tolerate either drug or something?

S: No.

I: Alright. And then how, if at all, does the availability of generic TDF/FTC but not TAF/FTC influence your prescribing?

S: Say that one more time?

I: How does the availability of generic TDF/FTC but not TAF/FTC influence your prescribing?

S: Oh, like it doesn’t terribly influence it now, because there’s an exclusivity situation, and so from a cost standpoint, I don’t think it’s really turning heads, it’s not turning payers heads here in Massachusetts. I’m in the process of trying to put together a grid about this, for my staff. And for the most part, payers are kind of sticking with the, prioritizing Truvada, the brand.

I: Prioritizing the brand name over the generic?

S: Yes. And not making people have to go to the generic first.

I: Okay. Alright, any other experiences or thoughts that you have about TAF/FTC vs TDF/FTC that you would like to discuss?

S: No, just that you know, it would be great to get the indication changed so that they can be truly interchangeable.

I: Great. So that was the end of our initial questions, because we started this before COVID, we’ve now tacked on a couple of COVID-related questions. So as a prescriber, have you noticed any effects of the COVID pandemic on your prescribing practices for PrEP?

S: Um, just that you know, we’ve had to change our work-flows. We use telehealth now, and that creates an uncoupling between the visit and the education and the prescribing from the lab testing and the actually picking up of the med. So we have our own pharmacy, so before that would all happen in one event. And now it doesn’t.

I: Um, have you had any patients report any changes or effects that the COVID pandemic has had on their PrEP usage?

S: So I think that uh Doug Krakower put together a study that showed that like um, the percentage of patients, that the number of patients who were filling PrEP prescriptions dropped significantly during the pandemic. This was a study done using Fenway patients. So thank you Doug, for that. I think there’s probably a number of reasons why that happened. The prescription data maybe incomplete, would be one thing to keep in mind, especially since a lot of people went away, they went to other states, they went to live with their parents, they did whatever. And so we may not necessarily be getting prescribing data that is as accurate. The other thing is is that hopefully, and it’s not too hard to believe this for some patients, our patients risk level went down. I mean, people stay away from each other, so. Maybe they engaged in you know, in safer types of sexual behavior, that didn’t warrant having to take this PrEP.

I: Okay, great. Any other thoughts about PrEP and the COVID pandemic?

S: No.

I: Great. And then, that’s basically the end of the interview.
