# Supplementary material for: Health care providers’ decision-making and early adoption of tenofovir alafenamide for HIV preexposure prophylaxis: An inductive qualitative study
Source: PLoS One. 2024 Dec 5;19(12):e0311591. doi: 10.1371/journal.pone.0311591 (PMC11620414; doi:10.1371/journal.pone.0311591)
Supplement: S1 File — (ZIP) [file pone.0311591.s001.zip › Clean transcripts/DedooseDoc_Participant 16 Transcript.docx]

I: I am going to ask you a few questions to learn what you have heard and what you know about using tenofovir disoproxil fumarate with emtricitabine (hereafter TDF/FTC) vs. tenofovir alafenamide fumarate with emtricitabine (hereafter TAF/FTC) for PrEP. Have you heard about using TAF/FTC vs. TDF/FTC before today?

S: Yes I have.

I: And what have you heard about TAF/FTC vs TDF/FTC?

S: TAF/FTC has lower risk of bone and kidney toxicity, but does have a higher likelihood of affecting metabolic syndrome and weight gain. Both approved for use, Descovy not generic, so more expensive.

(unintelligible, both talking)

I: I have some more questions that kind of repeat themselves, we’ll probably cover it. So what are some sources of your information about using TAF/FTC vs TDF/FTC? Some options would be colleagues, patients, pharmaceutical reps, advertising, journal articles, continuing medical education, online information or others?

S: I would say all of the above, but one of my favorites is Julia Marcus’s infographic.

I: Alright. Have you received any guidance or feedback from medical staff at your institution regarding the use of TAF/FTC vs TDF/FTC for PrEP?

S: Um, just in the form of the CME/HIV conference

I: So then walk us through your thought process on how you make decisions regarding prescribing one or the other of these two PrEP options.

S: In general, my practice is to continue to prescribe Truvada to all patients, um, as at sort of like a baseline decision. The exceptions would be patients with underlying renal disease, for which Truvada might be contraindicated, or they might be at higher risk of kidney disease. And then in a few patients they have had decreased tolerability with Truvada for one reason or another. In which case, we have considered a trial of Descovy, and in those cases they have tolerated Descovy better, and I’m not sure why that would happen, because I think the side effect profiles from like a nausea/vomiting standpoint are pretty similar. But I have given them the option in that case. Those are really the only two scenarios in which I would consider Descovy over Truvada. I do have one patient on my panel who had a preference for Descovy over Truvada, because of the fear of kidney and bone toxicity, but did not himself have a risk of kidney or bone toxicity. And that’s the only situation in which I prescribe Descovy without other risk factors.

I: So with that patient who had a patient preference, but not actual clinical indication, did you prescribe him the TAF instead of the TDF?

S: I did.

I: Okay. Are there any insurance considerations, cost considerations?

S: Most definitely. So that’s why I’m always turning to Truvada first, because of the generic cost difference. And I have had issues getting Descovy approved for that one patient, it does require in fact a prior authorization to get him Descovy.

I: Okay. So then, what are some reasons, patient characteristics that would influence you to avoid a TAF-containing regimen?

S: You know, patients who are at baseline overweight or at risk of metabolic syndrome, I would have a discussion with them about my concern for that being more of a risk factor to them then the bone and kidney toxicities associated with TDF, so I think in that case Truvada would be preferable. That would be the big thing.

I: Okay, and then the same question – so what reasons or patient characteristics would influence you to avoid a TDF-containing regimen?

S: Underlying kidney disease. Or risk factors for kidney disease.

I: Any risk factors in particular?

S: Um, maybe if they had uncontrolled diabetes, hypertension with a significant proteinuria at baseline, I might be concerned about their risk factors for a second hit to the kidneys with TDF.

I: Okay. And then, what experiences have you had using TAF/FTC for PrEP?

S: Again, it’s really just that one patient whose preference was for TAF-containing regimen. All of my other patients I have on TDF for pre-exposure prophylaxis.

I: So you have...

S: And that was entirely a patient preference

I: So you haven’t run into the case where based on any patient characteristics, you’ve put them on TAF?

S: No

I: No, okay

S: No other patient has met that criteria of underlying risk factors, luckily. I did have one patient actually who did have a rising creatinine, and this was actually before Descovy was approved, anyway, but he would not have been a patient in whom I would recommend Descovy, because of his, because of a very thorough discussion of his sexual practices and risk factors for potentially acquiring HIV, it was clear that he was having very spare and intermittent intercourse, that was very much pre-meditated, and so we had a long discussion about doing on-demand pre-exposure prophylaxis with him, and he felt very comfortable with that approach. So he uses TDF, but on demand, because he in particular, has very pre-meditated situations in which he knows he is going to potentially be at risk of acquiring HIV.

I: Makes sense. So then do you have any patients currently on your panel on TAF/FTC for PrEP?

S: Just that one patient who’s...

I: Okay, and the follow-up questions we’ve more or less sort of addressed. There’s what factors influence your decision to prescribe a patient a TAF-containing regimen?

S: Entirely preference. And I actually did encourage him to do Truvada. He tried Truvada, and felt like he was having side effects to Truvada, and prefers to be on Descovy. I can't tell you that there’s really any clear side effects.

I: Okay. What side effects was he describing?

S: I think at the time it was maybe fatigue and nausea, so I don’t know that they were related. I don’t feel confident that they were true side effects. I think it was the mental association with bone and liver, and renal toxicity.

I: Yeah. Makes sense. So then, for patients who wish to be newly started on PrEP, do you tend to prescribe TAF/fTC or TDF/FTC and why?

S: TDF/FTC for the cost, safety profile, the generalizability to the population that I can prescribe it to – women, women who are of child-bearing years with the safety.

I: Okay. So then, for patients who are already on PrEP, to what extent, if at all, are you switching patients from TDF to TAF, and if so why?

S: I have not been in the scenario of having to switch. The only situation I would switch would be if the patient was having kidney toxicity, or some personal preference.

I: Um, what are some questions or concerns that your patients have raised regarding TAF/FTC?

S: Some of them have mentioned weight gain, but I don’t know that that is as publicly known, as the kidney and bone toxicity associated with TDF.

I: Okay. And then, um, so the same question but TDF/FTC - what questions or concerns have patients raised about TDF/FTC?

S: Yeah, kidney and bone toxicity. They’re all aware of that. And the monitoring.

I: Has anyone raised any issues, questions about effectiveness, other side effects, costs, coverage, pill size?

S: People haven’t been concerned about pill size. They haven't been concerned about side effects. I think that it’s widely publicized, widely well-tolerated. I think the cost people do bring up, though I have not personally run into situations where a cost was prohibitive for a patient. Either patients were able to afford the medications co-pay, or we have gotten them PrEP-DAP, which has been effective for helping them to afford the medication. Or used, previously before TDF was generic, just used the, is it Gilead, right?

I: Mmmhmm

S: Gilead’s coupon.

I: Okay. Um, if you have

S: Gilead has TDF or Gilead has TAF? I don’t remember?

I: I actually am not sure off the top of my head, don’t they have both?

S: No, cuz that’s why it’s not coformulated.

I: Yeah. Not sure... Um, so if you have any patients who have been switched from TDF?

S: TDF.

I: TDF is Gilead?

S: Mmhmm.

I: Makes sense. So then for patients who’ve been switched from TDF to TAF if any, how has their experience been?

S: For patients that have been switched from TDF to TAF? They’ve one fine on TAF.

I: Okay. And then

S: Um, they definitely have noticed, perhaps a slight weight gain. And in that one particular case, there’s no other reason for weight gain.

I: MMhmm. And then, for any patients who are newly started on PrEP with TAF/FTC, how has their experience been?

S: Um, I haven’t started somebody on TAF-containing regimens without previously being on Truvada.

I: Okay, has anyone reported any adverse events or negative effects with TAF/FTC?

S: No

I: Okay. And then, do you have any patients who have switched from TDF/FTC to TAF/FTC and then switched back to TDF/FTC?

S: Not to date.

I: Okay. Then, how, if at all, does the availability of the generic TDF/FTC but not TAF/FTC influence your prescribing?

S: Um, probably a pretty good amount.

I: Influencing towards TDF?

S: Towards TDF.

I: Yeah. Okay. Um, any other experiences or thoughts you have about TAF/FTC vs TDF/FTC that you would like to discuss?

S: Um, I think I’ve probably said the main thoughts that come to my mind when making most decisions.

I: Great. So that’s kind of the end of the initial portion of the study – we started this before the COVID pandemic but have like tacked on a little addendum, which just two COVID-related questions. As a prescriber, have you noticed any effects of the COVID pandemic on your prescribing of PrEP?

S: I haven’t. I’ve still had plenty of interest in initiating and continuing pre-exposure prophylaxis. A lot of my experience with new patients comes because I precept residents, so the residents are still seeing a very significant number of patients establishing care in our practice, whose chief concern is initiating Pre-exposure prophylaxis. One of my concerns was that we didn't have the same pharmacist accessibility, for managing patients won pre-exposure prophylaxis in HAC as we previously did prior to COVID, now that’s back, up and running. And so I feel a little bit more that we can have patients come in on a regular basis and have a thorough discussion of how they’re doing on the medication and get screening STD tests and labs.

I: Okay. And then, have you had any patients tell you about any effects that the COVID pandemic has had on their PrEP taking or PrEP risk factors?

S: I think people’s risk factors have declined significantly. That people are not as active, as they were prior to the pandemic, so perhaps their risk factors have decreased, though I do find it interesting that probably in the last 6 months there have been 10 new patients in the resident practice who have requested PrEP none-the-less.

I: Okay. Is that more or less than before? Or about the same?

S: About the same, I would say. Probably one a week, one every other week. And that’s just with me as the preceptor, not other preceptors.

I: Makes sense. Any other thoughts about the COVID pandemic and PrEP?

S: No those are the main things. I mean, I think continuing to recognize it as an important option for patients despite the pandemic. I think people think like, the risk doesn’t exist. But I think knowing people are still asking for it is very pertinent, and so still finding ways to make it possible for patients I think now more than ever, we’re in a world of self-swabs, and so you know, if there’s any benefit of a pandemic, it’s understanding that patients should have the flexibility to continue to take these medications, find ways for them to do self-swabbing, so they can continue to receive the medications that they need.

I: Okay. Excellent. That’s basically the end of the question portion.
