# Supplementary material for: Health care providers’ decision-making and early adoption of tenofovir alafenamide for HIV preexposure prophylaxis: An inductive qualitative study
Source: PLoS One. 2024 Dec 5;19(12):e0311591. doi: 10.1371/journal.pone.0311591 (PMC11620414; doi:10.1371/journal.pone.0311591)
Supplement: S1 File — (ZIP) [file pone.0311591.s001.zip › Clean transcripts/DedooseDoc_Participant 17 Transcript.docx]

I: I am going to ask you a few questions first to just learn what you have heard or know about using tenofovir disoproxil fumarate with emtricitabine (hereafter TDF/FTC) vs. tenofovir alafenamide fumarate with emtricitabine (TAF/FTC) for PrEP. Have you heard about using TAF/FTC vs. TDF/FTC for PrEP before today?

S: Yes

I: Okay, and what have you heard about TAF vs TDF?

S: Um, I think we have a little bit of information about using TAF, not quite as much as Truvada, but um, or TDF, sorry. And actually I would say most of the time patients actually bring the question to their appointment. And so I think that’s kind of the setting that it’s come up the most.

I: Okay. And what are some of the sources of information, of your information, for using TAF/FTC vs TDF/FTC for PrEP? So some examples would be colleagues, patients, pharmaceutical reps, advertising, journal articles, CME, online education, or others?

S: Um, patients? And then I’ve definitely looked at some of the, like one of Doug’s paper’s, that small review in the Annals. And then I’m sure I’ve looked it up on like UpToDate slash Google probably. Um, yeah.

I: Great. Have you received any guidance or feedback from medical staff at your institution regarding use of TAF/FTC vs TDF/FTC?

S: Mmm... No? I don’t think so.

I: Okay. So walk us through your thought process on how you make decisions regarding prescribing one or the other of these two PrEP options.

S: Um, I think that my thought is that there is a chance that using the TAF preparation could have less effect on bone loss and maybe less effect on renal dysfunction. Um, even though we have more experience with TDF, and um, I think I probably think of it a little bit more if the patient is really young. Like I think for adolescents and like young twenty-something year olds that may be on it for a while, that’s something to consider.

I: You would consider TAF you mean for people who are young?

S: Considering TAF, yeah. I think the only, I’m not sure... My impression is that it’s been harder to get through insurance, or pricing differences? And so usually when we talk about that, at the appointment with the patient, I’ve only had a couple who have actually pushed for having the TAF formulation. It’s usually been kind of like a back and forth explaining like “this is kind of what we know and what I think is probably the difference, that it probably is just as effective as TDF, but theoretically the studies aren’t quite as, um, robust isn’t quite the right word, but as big”.

I: MMhmmm. And then what specific factors would make you recommend TAF/FTC over TDF/FTC?

S: Um, I think, if they had probably... creatinine clearance... and I guess if they had a known... If I was really worried about bone density, which isn’t many patients. So I haven’t actually used that. But that’s my theoretical framework for when I’d probably use it.

I: That’s fine! Yeah. And then, are there any specific factors that would make you recommend TDF/FTC over TAF/FTC?

S: Uh, no. I mean, I think there’s more experience with it, but after that I don’t know that I would feel strongly saying one or the other.

I: Okay. How do patient preferences come into play?

S: I have a lot of patients who talk to their friends, and say they would like to switch, or they come in for their first appointment, and so generally if that’s the case, sorry, that they would like to start with TAF or change to TAF from TDF, so usually in that case I have tried it – I've had some patients where it costs more money so they ended up not doing it.

I: And then, any, you already sort of answered this, but are there any patient characteristics that would come into play? Like gender, medical conditions...

S: Oh, same stuff I guess. Kidneys and then bones. So we don’t really have many of those. But theoretically.

I: And then any insurance or cost considerations?

S: Um, I don’t know much beyond I have to ask, I ask for help from pharmacy if we think that we’re considering it, just to see if it would... if they could have assistance or how much it costs?

I: Ask help from pharmacy if considering TAF? Or just if considering PrEP in general?

S: Oh, I guess either. But in particular if I really, if the patient felt really strongly and we thought TAF would be a good one it’s like an extra push to make sure we touch base with someone.

I: Okay, and then are there any reasons or patient characteristics that would influence you to avoid a TAF-containing regimen?

S: Mmm. I don’t know. I don’t have any off the top of my head.

I: Okay. And then the same question but for TDF, any reasons or patient characteristics that would influence you to avoid TDF-containing regimen?

S: A really low creatinine clearance. I mean, it’s still, really it’s a problem with both of them

I: Yeah. Fair. And then what experiences have you had using TAF/FTC for PrEP?

S: I have only had a couple patients. Most of them were new starts. The patient came to clinic and wants to start PrEP and had talked to their friends and their friends said “Have you heard of the TAF-containing regimens?” And then I’ve probably had a handful of people that are like my chronic patients that are on TDF for TAF, sorry TDF for PrEP, that asked about TAF, but ended up just deciding not to switch, because they didn’t feel like changing their sort-of status quo. Um, but yeah.

I: Okay. Um, the next question – do you have any patients on your panel on TAF/FTC for PrEP?

S: Probably less than 5. It’s probably like only a handful

I: Okay. And then what factors influenced your decision to prescribe the patients you have on TAF the TAF containing regimen?

S: It, they were actually very patient driven.

I: Okay.

S: Yeah.

I: Great. And then, sort of like, very similar question, just describe your decision-making for selecting TAF-FTC for any new starts of PrEP, um versus for any switches?

S: Yeah, I mean I think it’s kind of the same thing. I usually have a conversation with the patient and ask them if they’ve heard of both

I: Mmmhmm

S: Which sometimes they have, and then, kind of explain the differences, and I think the main catch being it hasn’t been the same, I feel like it hasn’t been quite as easy to get TAF as it is to get TDF. Um. But I mean, I think we tried, I try to at least bring it up when I talk to patients, but again, generally they actually have already looked into it, or they bring like a flyer with them, or something like that.

I: Okay. Um, let’s see. Uh, any potential risks and benefits that you weight when deciding to prescribe a TAF vs TDF-containing regimen?

S: Um, versus each other kind of the same things we’ve said. I think theoretically bone loss. But I mean, outside of that I think of them kind of similarly.

I: Okay. And then, for patients who wish to be newly started on PrEP, would you tend to prescribe mostly TAF/FTC or TDF/FTC and why?

S: I mean, I have generally probably done more TDF. But I haven’t actually had many people recently who’ve asked about TAF. Um, and some of that I think was depending on who I was staffing with, maybe they just felt like it would be better to do TDF. Um, or I think their big concern would be that we would prescribe it and the patient couldn’t afford it and maybe wouldn’t come back to clinic and kind of lose that ability to capture and establish care with them.

I: Mmhmm. And then, for patients on PrEP, to what extent, if at all, are you switching patients to TAF from TDF containing regimens, and why?

S: Pretty rarely. But, mostly driven by patient requesting in the visit.

I: Okay. And then, actually perfect seque. So what are some questions or concerns that your patients have raised regarding TAF/FTC?

S: Um I feel like they usually ask “My friends told me that this would be better for me” They usually say less side effects, but every once in a while, I feel like they’ve specifically said “this might be better for my bone health” and surprisingly not many of them have asked me about if it’s as effective. I mean, I think they mean that when they’re asking about it, but they don’t actually ask that outright the same way they have with side effects.

I: Alright. And then, the same question but for tDF/FTC - any concerns or questions that patients have raised regarding TDF/FTC?

S: Not in particular, just the general “What are the side effects?” And then I do think people ask about some of the options for like on demand PrEP occasionally, if they’re kind of well-versed or have friends who have been in New York or places that have pitched that.

I: Um and then you mentioned that people have asked about side effects – are there any particular side effects that people have asked about, for either of the two?

S: Um, I know I have had people ask me about bone health. I think someone, I’m sure someone has asked me about renal dysfunction. And then I feel like people always ask about liver in particular, I don’t know why... probably because it’s just listed on every insert of every med?

I: Have you told them anything different about liver for one vs the other? Like what do you tell them when they ask about liver?

S: Well, I kind of get back and say “I wonder if what you were looking at is renal dysfunction, because I think that’s probably what’s more important” because I feel like if sometimes people, if they’re mixing it up, I don’t want them to just say “oh I’m not really worried about hepatic dysfunction” and ignore what they probably may have actually read about.

I: Right. And then, any patients bring up any concerns about insurance converage, out of pocket costs, pill size?

S: Um patients usually will ask me kind of the general things like “do you think it would be covered?” That kind of stuff. I’m trying to thing if they’ve ever asked me anything more. Mmm not that I can remember.

I: And like similar rates of asking for both of them I would assume?

S: Yeah. Yeah.

I: And then, for any patients who have been switched from TDF/FTC to TAF/FTC, how has their experience been?

S: I haven’t had any... they’re doing okay I guess. One of them I didn’t see again, so I’m not sure if they started going to their primary care doctor. And I haven’t seen the other patient... Yeah, there’s definitely someone I did TAF and then they didn’t come back for their follow-up. So I don’t know...

I: Was that a switch or a new start?

S: That was a new start. Young person

I: Okay. Have you had anyone that you’ve put on TAF, either switch or new start, that you have had some follow-up with?

S: Probably only one or two

I: And how was their experience?

S: It was fine – they're doing well.

I: Do you remember if those were switches or new starts?

S: One I’m thinking of was definitely, was a switch. And I think the other one was a new start. And then the new start I didn’t see. So I probably only have like 3 or 4.

I: Um, so any particularly positive experiences that patients have reported, or adverse events or negative effects?

S: Um, nothing that stands out. Like not anything negative I can remember. And then I think not having a problem is sort of, is good, so...

I: Okay. And then, have you had any patients who’ve switched from TDF to TAF and then switched back?

S: Uh, I’m pretty sure I’ve had someone who I prescribed it, and then they never started TAF.

I: And why was that?

S: I think because of however much it was going to cost, I guess TDF was cheaper.

I: So you had one attempted switch that didn’t actually make the switch?

S: I couldn’t really call them a switch, but I’ve definitely had that. I guess that was a while ago.

I: And that was failed because of cost?

S: Yeah. I don’t remember the price difference.

I: Yeah. Um, and then how, if at all, does the availability of generic TDF/FTC, but not TAF/FTC, influence your prescribing?

S: Uh, I don’t know. I mean, in my head I think in most cases that TDF is going to end up being cheaper. So that would make me kind of lean towards that, if I recommend it to a patient. Um, but if you took that away, I’m not sure that I would feel strongly.

I: Okay. Any other experiences or thoughts that you have about TAF/FTC containing regimens that you would like to discuss?

S: Mmm... I don’t think so. I think it’s been very interesting to see how patients usually have like flyers, or they’ve looked it up. I mean, I think it’s because of the whatever community that they connect with, but it is very interesting to see how they’ve tried to be really thoughtful and bring that forward, even though I feel like there’s really not a lot of info out there, for like non-medical providers. Like, if, I feel like if you were just a patient it would be really hard to know what’s the difference. And, um, like why should I even ask, if you didn’t have like a friend who had talked to their doctor who had talked to you. And maybe those exist, and if they do it would be awesome because I could send people to them.

I: Yeah that would be great.

S: I’m guessing there’s not a ton of them out there, but, yeah

I: Not that I’m aware of, but yeah that would be awesome. So that’s the end of the like, TAF/TDF ones. Because we started this like, before COVID, and now are like, still doing it, we tacked on a couple of COVID questions, because like, why not. So the first one is “As a prescriber, have you noticed any influence of the COVID pandemic on your PrEP prescribing?”

S: Um, I mean, there definitely was a gap where I feel like I wasn’t seeing those patients. So like, a routine PrEP appointment was more likely to sort of get pushed than some other ID outpatient issues. And I think that’s like also, the other complicating part is like not being able to get gonorrhea/chlamydia testing easily for patients... Like their screening, STI checks, at those appointments also, outside the fact that they weren’t there, just like not actually having the media to do the test. Um, and I haven’t, I don’t think I’ve seen anyone for a new start since COVID.

I: Okay. And then from a patient perspective, um, have you noticed or been told by any patients about any effects of the COVID pandemic on them and their PrEP use?

S: Um, I definitely have had one or two patients who took a break. Um, because they were like “I’m in my house, I’m not going to interact with anyone, I could have lost my routine X, Y, and Z.” Like those types of things, but um generally they resumed, I don’t if that’s also because they had an appointment, and they felt like they should restart before they come in for their appointment. Um, but that’s probably been the most common thing, outside of like the impact on their general lives. As far as PrEP it’s definitely been a couple of people who’ve put a pause on it.

I: Great. Any other thoughts about COVID and PrEP?

S: Mmm... I don’t think so.

I: Awesome. Well that’s the end of the questions.
