# Supplementary material for: Health care providers’ decision-making and early adoption of tenofovir alafenamide for HIV preexposure prophylaxis: An inductive qualitative study
Source: PLoS One. 2024 Dec 5;19(12):e0311591. doi: 10.1371/journal.pone.0311591 (PMC11620414; doi:10.1371/journal.pone.0311591)
Supplement: S1 File — (ZIP) [file pone.0311591.s001.zip › Clean transcripts/DedooseDoc_Participant 18 Transcript.docx]

I: I am going to ask you a few questions to learn what you have heard or know about using tenofovir disoproxil fumarate with emtricitabine (TDF/FTC) vs. tenofovir alafenamide fumarate with emtricitabine (TAF/FTC) for PrEP. Have you heard about TAF/FTC vs. TDF/FTC for PrEP before today?

S: Yes

I: Okay and what have you heard about using TAF/FTC vs TDF/FTC?

S: It is likely comparable, but we just need the information at this point. A nd there are issues of like, cost as well as other sort of non-clinical reasons why we don’t have both those options for patients.

I: Okay. And then what are some of the sources of your information about using TAF/FTC vs TDF/FTC for PrEP? Some options would be colleagues, patients, pharmaceutical reps, advertising, journal articles, continuing medical education, online information or other?

S: I would say colleagues and conferences primarily.

I: And then have you received any guidance or feedback from medical staff at your institution regarding the use of TAF/FTC vs TDF/FTC?

S: No formal guidance

I: You said no?

S: No, no formal.

I: Alright. Any informal guidance?

S: Yes. I would say that there’s definitely been discussion, and I’ve had people in the division answer my questions as to what we should do about it and they have provided their thoughts.

I: Okay. So walk us through your thought process on how you make decisions regarding prescribing one or the other of these two PrEP options.

S: I think for better or for worse, um, a lot of it. I think first and foremost I do think about the patient themselves, just the overhanging view I do think about whether or not the patient is interested, wants to, is engaged in care, and will be compliant with PrEP overall regardless of whether or not it TDF or TAF based. Then I do want to get labs and see if there’s any clinical reason why I should be opting for one or the other, be it more renal function, if they have low creatinine, or underlying renal disorder, other considerations as well. Based on that I might then opt for one or the other. Last but not least, sometimes even if there is one I might prefer, I do also want to consider insurance considerations as well, if it would be costly for them. I have been burned in the past when I have had a couple patients who have wanted one formulation or the other and then we found out, you know, after they’d left clinic, that one was cost prohibitive, and we had to have that conversation on the phone as to why we could not follow through with the plan that we had discussed with them

I: Okay. So what are some reasons or patient characteristics that would influence you to avoid a TAF-containing regimen?

S: Mmm – I guess kind of what I had said before. I might think about, for clinical reasons, like renal function. I had one patient who we didn’t do it because they only had one kidney, so uh

I: Mmmhmm

S: That was, that was like, you know. That was a consideration that we put out, an exception obviously not everyone is walking around with one kidney, but, that would be more I think about and ask.

I: Okay. And then the same question for what reasons or patient characteristics would influence you to avoid a TDF-containing regimen?

S: Not too many I would say, not too many.

I: Okay. And what experiences have you had with using TAF/FTC for PrEP?

S: I haven’t had too many patients, but I’ve had maybe like a small handful, and I’ve had no problems whatsoever. Ultimately, the patients that I prescribed it to were compliant or engaged. They were actually the ones who also we had had long conversations about it, so the compliance, we never had any issues. We checked safety labs as well, so from a medical perspective as well, nothing.

I: Okay. And then, in the past year, have you had any patients on your panel on TAF/FTC for PrEP?

S: I haven’t started anybody new since last year.

I: Have you had anyone who’s on your panel at all? Like both new starts or old starts?

S: I had two.

I: Okay. What factors influenced your decision to prescribe the patient a TAF-containing regimen for PrEP?

S: One patient actually came to me seeking specifically a TAF-based regimen, and we kind of talked about the pros and cons – they were actually referred over, um, specifically to have that conversation with us, depending if they doctor didn’t feel comfortable. So um, it was very straightforward – this was like a young person who really had no medical issues and very well informed, came very prepared. So after doing baseline labs, my preceptor and I felt no reason to hold back, so we started it for that patient. The other one, that was mostly in the context of like I mentioned, has only one kidney, so we opted to pick something we hoped would sort of preserve renal function for a little longer.

I: Alright. Um, can you tell me more about that patient, the patient who came in asking about the TAF containing regimen?

S: Yeah, so this patient was very healthy 20 something year old male, no underlying medical issues, is in health care field, so had done a lot of homework and was very proactive in taking care of themselves, and so actually approached the primary care doctor seeking to start PrEP. Actually, you know like brought in articles and things is what I remember this person saying. But because it was something that was something that was somewhat unfamiliar to the primary care doctor, they weren’t sure whether or not it was the “right thing” or the gold standard at the time. So said, you know, why don’t we have an ID doctor weigh in, just to make sure this seems okay. And it was a brief, I think I saw him once in person and then followed up once on the phone. Felt very comfortable after I’d emailed this question with the primary care doctor that you know, we endorse this, this all made sense and was above board. Went back to the primary care doctor, and as far as I know has been continued on the TAF-based regimen, we haven’t heard anything since.

I: Have you had any patients who have switched from TDF/FTC to TAF/FTC?

S: No actually, now that you mention it, no.

I: Okay. Um, alright. Um, are there any reasons that you have not or wouldn’t start a patient on TAF specifically?

S: There’s... I don’t think so... not really... not anymore

I: And then, what are potential benefits and potential risks that you would weight when deciding to prescribe TAF vs TDF?

S: I think kind of like we had talked about, like if they’re any older person I might think about bone health, renal function, I might talk to them about family history, their own personal history... Those would be the only things.

I: Okay. Alright, and then so for patients who wish to be newly started on PrEP, would you tend to prescribe mostly TAF/FTC or TDF/FTC, and why?

S: I feel like at this point, I think reflexively I still feel like I end up prescribing TDF based, but there’s nothing that’s keeping me... there’s nothing specific... There’s nothing specific that would keep me from picking a TAF based regimen. So I think it would, probably just old habits and reflex is the only thing at this point.

I: I think you already answered this next question, to what extent, if at all, are you switching patients to TAF from TDF containing regimen?

S: I haven’t, I’ll be honest.

I: Yeah, no – that's fine. Um, and then what are some questions or concerns that your patients have raised regarding TAF/FTC?

S: I would say of the one patient, the one that came very prepared, the question was primarily just about efficacy and cost, I would say actually those, those were the two questions. One – was there any data from an ID consultants perspective that would suggest a lack or a diminished efficacy, and whether or not it was significantly more costly. And I think those were really the key things that he had asked me.

I: Great. And then any questions or concerns that your patients have raised regarding TDF/FTC?

S: Actually yes, now that we’re talking about it, I did have one patient just ask me point blank, like “isn’t this the “Old Stuff”, like aren’t there other, newer things, we could try?” That I remember quite clearly.

I: What ended up happening in that case?

S: I told that patient there’s really nothing keeping them if they wanted to change it was perfectly fine, but there was nothing to suggest that it was, just because it was older that it would be less effective. And so ultimately the conclusion that was that kept on TDF based. He was like “well, you know, if for some reason that information changes you would switch me right?” And I was like “Oh yeah... absolutely”

I: Great. Um, and then for patients who, doesn’t really apply, this next question is about patients switched from TDF/FTC to TAF, how has their experience been... So I’m going to skip to the next one, which is how has the experience been for patients who are newly started on TAF/FTC?

S: I’ve had of those two, they went really smooth. Like I mentioned one of them I did the phone update, completely fine. The other one, completely fine. But like I said, young and relatively healthy?

I: Any adverse events or negative effects that you noticed?

S: Nothing that was brought up to us

I: Okay. Um, and then I’m assuming the next question doesn’t apply either – it says tell us about any patients who switched from TDF/FTC to TAF/FTC and then switched back.

S: Not in my case, no.

I: And then, how at all does the availability of generic TDF/FTC but not TAF/FTC influence your prescribing?

S: Oh yeah, it absolutely does. Because it’s one, you know just from a user-face standpoint, I feel like I have more financial ground to stand on when I talk to patients who either they don’t know their insurance status or they’re concerned that they may not be able to pay. It is in a way sort of like safer not to play this game of phone tag and sorting out the insurance company, just knowing that TDF is generic, we can get you started, like we don’t have to wait. Also, like I had mentioned before, I think reflexively, because it’s sort of what’s been taught, it’s easier to reach for that because it feels and intellectually, like well I know it works, that’s what we’ve been doing for a while now. Like you know, I don’t have to think too hard about it. So obviously that could change.

I: Right. And then are there any other experiences or thoughts that you have about TAF/FTC containing regimens that you would like to discuss?

S: I just wish it was more readily available, honestly. And I also think that, you know, I think for a lot of people it’s starting to become like, equal footing, with TDF-based. I j ust wish that some of those obstacles that you kind of brought up in this discussion like cost, or you know, it’s sort of what we know, like, or it’s what we classically start people on. I wish those paradigms would change, just so people understand it’s basically equal footing, there’s really nothing clinically that should keep us from prescribing it.

I: Okay. Great. That concludes the TAF/TDF portion. Just because of the timing of this we also tacked on a couple COVID-related questions.

S: Oh might as well, yeah

I: Okay, so as a prescriber, have you noticed any influence of the COVID pandemic on your prescribing practices for PrEP?

S: Yeah... yes. Because so many of the clinic appointments are now phone calls, I do feel that we try to keep the clinic appointment very tight and very focused, and so I do think that there have been some opportunities that I think in person with the right context and the right conversation we could have initiated a PrEP talk, much more quickly, much more smoothly. But on the phone, that 15-minute interaction makes it a little stilted. You do have to literally switch gears sometimes to have that talk and I think that likely, if I’m going to be totally honest, probably some missed opportunities to start some people on PrEP. U m, I also do think that there is this sort of underlying, BAD assumption that like people aren’t just having sex. Or like that people are going to be at home. But that’s not necessarily true. People still need human interaction. Again, like probably a bad assumption leading to other bad assumptions. Probably could have been prescribing more PrEP, total honest.

I: Great. And then have you noticed any effects of the COVID pandemic on your patients who are taking PrEP, like from a patient perspective, anything they’ve told you?

S: No, nothing... I’ll be honest, nothing in particular.

I: Okay.

S: A lot of them actually ended up having like phone visits cancelled with me, just because their like acuity “was low”. But there are some that just, you know, by the time I had wrapped up clinic, I hadn’t talked to them.

I: Okay. Great. Excellent. Well, that’s... Anything else about COVID and PrEP?

S: I’m just interested in what you guys find. I’m wondering if the telehealth experience has... yeah, I’m wondering what the telehealth experience has done for like the PrEP conversations... I’m curious to know what you guys find out.

I: Yeah. Yeah, it will be interesting. Yeah.
