# Supplementary material for: Health care providers’ decision-making and early adoption of tenofovir alafenamide for HIV preexposure prophylaxis: An inductive qualitative study
Source: PLoS One. 2024 Dec 5;19(12):e0311591. doi: 10.1371/journal.pone.0311591 (PMC11620414; doi:10.1371/journal.pone.0311591)
Supplement: S1 File — (ZIP) [file pone.0311591.s001.zip › Clean transcripts/DedooseDoc_Participant 19 Transcript.docx]

I: I am going to ask you a few questions to learn what you have heard or know about using tenofovir disoproxil fumarate with emtricitabine (TDF/FTC) vs. tenofovir alafenamide fumarate with emtricitabine (TAF/FTC) for PrEP. Have you heard about using TAF/FTC vs. TDF/FTC for PrEP before today?

S: I have yes

I: and what have you heard about TAF/FTC vs TDF/FTC?

S: So um standard of care, my understanding is standard of care is to use TDF/FTC. I think when we talked about this when Doug gave a lecture about this last year, at maybe the Wednesday conference, but the use of TAF/FTC was not, like widely practiced and my understanding from like maybe 2 years ago was that at that point there wasn’t enough data and there was some concern that potentially TAF doesn’t get to good levels in the mucosal surfaces so that potentially it wouldn’t be as effective as TDF. And the data that we have is with TDF/FTC so we use TDF/FTC. I know there’s some newer data that came out that I haven’t kept in touch with, so there might be something else recently that shows that TAF/FTC is just as good. And I would imagine that in patients that are like, I imagine like younger, who are going to be on it for longer, there might be benefit, but I don’t actually know.

I: Okay. Um, and then what are some of the sources of your information about using TAF/FTC vs TDF/FTC? So some possibilities would be colleagues, patients, pharmaceutical reps, advertising, journal articles, online information, others.

S: I would say colleagues and lecture, like attendings during lectures

I: Okay. And then have you received any guidance or feedback from medical staff at your institution regarding the use of TAF/FTC vs TDF/FTC for PrEP?

S: Uh, yes, just uh my information might be a little outdated, but the teaching that we got last year was to use TDF/FTC, which could be updated now.

I: And then walk us through your thought process on how you make decisions regarding prescribing one or the other of these two PrEP options? What are specific factors that would make you choose TAF/FTC over TDF, or TDF over TAF?

S: Yeah, I have only ever, I don’t think I’ve ever actually had a clinic patient that I’ve prescribed PrEP for. I’ve had a bunch of HIV patients, and I know one of my HIV patients had his girlfriend was on PrEP, but that was prescribed by her PCP, so I haven’t personally honestly prescribed it, but I’ve been part of discussions where people were prescribing it, and I, at this point I’m not sure. I know the concern, we had talked about this when I was a first year, was that there wasn’t enough data to prescribe TAF/FTC, so I would at this point without doing more reading I would not prescribe TAF/FTC, but I think that I would consider it in someone that had renal failure o r someone that I would anticipate, someone younger who I would anticipate would be on it for a prolonged period of time. That would be the kind of person, if there’s data to support using TAF/FTC, that’s the patient I would consider.

I: Okay. And then it asks do patient preferences come into play? Or any insurance considerations, cost considerations?

S: Yeah, definitely, I think that’s an unfortunate but valid point, and I would talk to our pharmacy team and see what our patient's insurance would be able to cover. Um, I know that with Gilead’s been pushing the TAF/FTC stuff, and there’s like a lot of... there’s more money to be made... and there’s all this patent stuff... but I don’t... I haven’t kept in touch with all the details.

I: And what are some reasons, patient characteristics that would influence you to avoid a TAF-containing regimen?

S: Patient characteristics that would make me avoid a TAF-containing regimen. So I know there’s some newer data talking about weight gain with TAF, um, and I’m... that might also be, that might be in the context of integrase inhibitors, so I don’t... s o I guess patient considerations that would make me avoid TAF would include patients that are unable to afford a TAF containing regimen, because it’s more expensive than the generic TDF. And then potentially weight gain.

I: And then, the same question, so any reasons or patient characteristics that would influence you to avoid a TDF-containing regimen?

S: Renal failure or I guess renal insufficiency would make me hesitate, and... other things for TDF... I think that’s the biggest thing that would make me reconsider.

I: Alright, so then some of the next questions you’ve sort of already answered but we’ll go through them anyway, but what experience have you had using TAF/FTC for PrEP?

S: I have no experience, sorry.

I: That’s okay. And then, do you have any patients on your panel who are on TAF/FTC for PrEP?

S: No, I don’t have a panel anymore.

I: Fair enough, so tell us about any patient inquiries... or have you had any patient inquiries or requests for TAF/FTC for PrEP? Either now or before?

S: No.

I: And then, how would you respond, if you had a patient inquire, or request TAF/FTC specifically?

S: TAF/FTC for PrEP?

I: Mmmhmm

S: I would, and this just might be a very specific to me, but just because I’m not familiar with data that supports TAF/FTC for PrEP I would first look to see what is known about it, and then uh, assuming that there is good data supporting TAF/FTC for PrEP I would see if cost is a barrier for them, and if so I’m sure TAF/FTC is more expensive than TDF/FTC, s o I would have a discussion with the patient about potential for less renal events with TDF, with TAF compared to TDF, and see if the cost is something that they are willing to I guess, if the balance works out for them.

I: Yup. Okay. Great. Um, and then, for patients who would wish to be newly started on PrEP, would you tend to prescribe TAF/FTC or TDF/FTC and why?

S: Um, I would prescribe TDF/FTC at this point. Um, just because we have a long track record with using TDF/FTC, we have a lot of data to support its efficacy, t here doesn’t seem to be, in patients that don’t have underlying renal disease there doesn’t seem to be a huge propensity for harm with this regimen, and most providers have substantial experience with it, so that’s what I would recommend.

I: And then, the next question I’m guessing probably doesn’t apply, it’s for patients on PrEP, to what extent, if at all, are you switching patients to TAF from TDF containing regimens?

S: No patients on PrEP.

I: Yeah, these questions are, so we’re doing these with IM residents, ID fellows and attendings and like Fenway and HCA attendings, so there definitely some of the aprts are like more targeted to certain audience, or certain parts of the audience.

S: I need to read about TAF/FTC now, I didn’t realize that this was a thing!

I: Honestly, that is the reaction that almost all of the fellows have had. So, you’re not alone. There’s just a couple more questions. So what are some questions or concerns, if any, that your patients have raised regarding TAF/FTC?

S: For PrEP? Uh, I haven’t had any questions about it.

I: And then, how about TDF/FTC?

S: Um, TDF/FTC, so the patient whose girlfriend. My patient who was newly diagnosed with HIV whose girlfriend was starting PrEP, he had no questions about it, but it was also the PCP who was prescribing it for the girlfriend. So I haven’t actually had that experience.

I: Fair enough, and then I’m guessing this, the next couple are probably not going to apply as well, so for patients who have been switched from TDF to TAF, how has the experience been? And how about those who are newly starting TAF/FTC?

S: No experience, sorry.

I: No, no – that's like I said, totally fine. This is like certain, I expect certain people to not be answering certain parts of the questions. So then tell us about any patients that have switched from TDF to TAF and then switched back... I’m assuming that doesn’t apply. And then, last question, or second to last, getting to the end. How, if at all, does the availability of generic TDF/FTC but not TAF/FTC influence your prescribing?

S: It would make me, I think especially because the patient population that often is in need of PrEP, often doesn’t have like, a lot of great insurance options, and sometimes copays are an issue. I’m more inclined to prescribe generics than to prescribe a branded drug like um, TDF/FTC at this point. I know we have a lot of support with our HIV patients in terms of acquiring medications that they need, but I don’t know what resources we have for helping with PrEP copays and stuff. So I would, I would lean towards TDF/FTC, unless there’s a compelling reason for me to use TAF/FTC.

I: Okay. And then, are there any other experiences or thoughts that you have about TAF/FTC containing regimens that you would like to discuss.

S: Um, sorry, experience or thoughts?

I: Yeah, about TAF/FTC containing regimens

S: Yeah, I don’t know.

I: That’s totally fine. The last two, probably not applicable, since you don’t have a clinic this year, but uh is, how if at all has the COVID pandemic affected your prescribing of PrEP?

S: Um, it had no impact on my prescribing.

I: And then, any impact that you’ve noticed from a patient perspective?

S: Um, for PrEP specifically?

I: For PrEP specifically.

S: No.

I: Yeah, okay. Great. That’s all the questions.
