# Supplementary material for: Health care providers’ decision-making and early adoption of tenofovir alafenamide for HIV preexposure prophylaxis: An inductive qualitative study
Source: PLoS One. 2024 Dec 5;19(12):e0311591. doi: 10.1371/journal.pone.0311591 (PMC11620414; doi:10.1371/journal.pone.0311591)
Supplement: S1 File — (ZIP) [file pone.0311591.s001.zip › Clean transcripts/DedooseDoc_Participant 2 Transcript.docx]

Interviewer: And we are set! Okay perfect. I'm just gonna record on here. All right, so let’s start with the first questions here. I'm pulling them up right now. All right. So I'm gonna ask you a few questions to learn what you have heard or know about using Tenofovir Disoproxil Fumarate with Emtricitabine versus Tenofovir Alafenamide Fumarate with Emtricitabine for PrEP. Is it okay if we use TDF for Tenofovir Disoproxil Fumarate and TAF for Tenofovir Alafenamide Fumarate.

Subject: Yes

Interviewer: Have you heard about using TAF versus anything... about using TAF versus TDF for PrEP before today?

Subject: Yes I have.

Interviewer: All right. What are some of the things just in general that you've been told or learned about the differences between the two and when you might use them.

Subject: so I know that. So I know that Truvada for PrEP is TDF and emtricitabine. I know that does Descovy for prep is TAF plus emtricitabine. I know that TAF is a pro drug. I know some benefits of TAF is that I think there's maybe some change in the amount of plasma concentration of drug such that there might be decreased renal tubule kidney exposure and bone exposure to the TAF formulation, so I believe there's less potential for bone and renal toxicity with TAF over TDF in prep formulations. I think that they are equally effective. Or that Descovy is non-inferior to Truvada in prevention of HIV

Interviewer: Okay, excellent! And where do you get your information for TAF and PrEP in general. It can be colleagues, journal articles, really any sources for example.

Subject: I would say colleagues, up to date, and I believe it's lexicomp for drug information. I guess lecture content as part of fellowship. Also speaking to the pharmacists with whom I work. Oh also journal articles.

Interviewer: Okay, all right. Is there any guidance that you've received from medical staff at your institution regarding the use of TAF versus TDF?

Subject: I think we've received… I think was mentioned in one or two lectures from ID faculty about the approval for TAF-based PrEP, but I don't think we've had like formal guidance on which patients might benefit from one formulation or the other.

Interviewer: Okay, and when prescribing prep are there any guidelines of an issued by your department or your institution?

Subject: That's a really great question. I'd have to look in the sort of ID division folder where there's a huge repository of different information about certain ID topics. I don't think there's a formal departmental guideline though.

Interviewer: Okay, all right great. Walk us through your thought process and how you make decisions prescribing one versus the other of these two agents for PrEP.

Subject: For my patients that are already on PrEP in the form of Truvada for HIV prevention I have asked them if they're aware of Descovy if the TAF formulation is an option and what their thoughts are, and we have a discussion about it. Most of the patients have seen commercials and seen advertisements about Descovy for PrEP. So sometimes they often ask me about it, but I typically talk about the benefits and drawbacks of potentially making a switch. I think if I'm worried about kidney function in my patient who's on Truvada or bone toxicity, then I would probably go towards prescribing or changing their PrEP regimen to Descovy that with the TAF formulation. But, I do caution them that TAF has an increased risk of weight gain. So if that's something they're concerned about, you know, we just have a discussion. For my patient panels on Truvada, I do think that I steer them towards Descovy, but it's sort of a shared decision making process.

Interviewer: Okay, and in general for steering patients towards Descovy, what's your reasoning behind Descovy?

Subject: I think reasons to choose Descovy is [that it is] safer for kidneys, safer for the bones, and it's as effective as Truvada.

Interviewer: Okay. And I'm how do patient preferences come into play, and how does that influence you one way or another?

Subject: Yes patient preference would influence me. Definitely. I think some patients have been on PrEP for several years now, which is great. It's worked for them. They have no side effects. Maybe they get it through a copay assistance program or it's free. It's sort of their routine and they don't see any problems so they want to keep on it and that's fine.

Interviewer: Okay great, and you mentioned co-pay assistance programs everything have there been any cost considerations when thinking these two; whether it's insurance or paying out pocket…does that factor in at all?

Subject: Yeah, totally. I am not sure of copay assistants or support programs for Descovy. I have not looked into that. But, I know that with Truvada you can get that covered, based on my experience. I believe there's a copy assistance program with Gilead. And so, I would I would definitely look into that. It would probably come to my attention if I had prescribed as Descovy for a patient and they were unable to pick it up due to cost or insurance issues. Then we'd have to work with them on how to get it.

Interviewer: Okay great. And then you mentioned renal dysfunction, bone health, weight gain things like that. In addition to those, or besides those, are there any specific patients factors such as gender or age or any other factors that would pursue to prescribe one versus the other.

Subject: That's a good good question. That's a great question. I think if a patient had some mild CKD or lesser than well-controlled diabetes with risk of kidney injury, I might push towards a TAF-based approach. I think if patients had like fragility fractures or like a big history of osteoporosis osteopenia I would go to towards TAF. I think their BMI was already high or there was a lot of concern about weight then I would think twice against TAF. I 'm not sure if age would influence me… Or gender. I don't think gender. S o I think that now that I'm remembering a recent Descovy for PrEP commercial, I don't know but I don't think it was studied in in cis females, but I still I still feel in my high risk patient that's cis female, I would consider Descovy, you know in place of Truvada,

Interviewer: Okay great. And then what experiences have you had using TAF… how many patients and what's your experience been like with that?

Subject: I wish I knew my exact [number]… so I would say that my patient panel for patients on TAF or TDF or base regimen, the vast majority have HIV infection. I think the proportion my panel that's on that's on PrEP for HIV prevention is much smaller… just me being an infectious disease [doctor] and seeing a lot of PrEP managed by primary care. I would say that I think it's probably 50/50 percent of folks on PrEP taking TAF versus TDF. My experience with TAF in general, also co-formulated with other ART, is [that] I have seen waking with it. My experience, so I do remember one patient that was on Truvada having some renal dysfunction, some increase in creatinine, and we switched to a TAF based approach and her creatinine improved and her renal function improved. So I have seen that effect. And in terms of numbers, oh gosh, I can't tell you. I honestly don't know how many PrEP patients I have in my panel. I might have like seven.

Interviewer: Okay

Subject: I have a lot more HIV patients.

Interviewer: Okay, yeah. Okay great and have you started anybody newly on TAF?

Subject: Yes, I have

Interviewer: Okay, and what has that experience been like? Have there been any concerns raised by the patient or on lab work monitoring, things like that?

Subject: I think that I am probably the one raising more of the concerns or words of caution. Like when educating them about potential side effects or adverse effects. I think that sometimes patients will ask me: “ hey, I heard this drug can lead to renal injury or I saw some class action lawsuit about TDF and renal failure. You know, is that going to happen to me on Truvada? I don't want to be Truvada anymore. I want to be on Descovy”. So, I think overall, after a good discussion about, you know, PrEP what it's used for and TAF versus TDF. I think the process of putting someone on a task-based regimen goes pretty smoothly.

Interviewer: Okay great and you had mentioned switching one patient with an elevator creatinine to a TAF regiment from Truvada. How many patients would you say you've switched for different reasons or did you find patients or asking to be switched?

Subject: Hmm. No, it's not a lot of patients are asking. Should I can include my experience with people taking TAF for HIV treatment or just for PrEP.

Interviewer: Let's just stick with PrEP for right now.

Subject: Yeah. I distinctly remember one patient asking to be switched to Descovy. Or asking: “ is there a safer one out there? I heard about a new one”. I just remember one doing that… asking me to, but mostly I'm initiating that sort of discussion. But, again my cohort is like seven people, so not big enough

Interviewer: Okay so not a ton. All right and then this may be a little repetitive, but what are some of the reasons that you wouldn't start someone on TAF. You mentioned potentially BMI. Are there any other reasons?

Subject: So I think reasons not to start on TAF…So I guess I know that this person is like, truly HIV negative?

Interviewer: Yes, that's true.

Subject: Okay. Um, so I guess I'd be worried in general if… I guess if they really had poor renal function to begin with like marginal creatinine clearance. I have to look at the exact guidelines. I don't know if it's a creatinine clearance less than 60 or 30, but if they had pretty significant CKD, I would really need to think about risk of harm with and renal dysfunction with TAF because it is possible, it's just decreased risk. So I think CKD comes to mind. Also I think if the patient has risk for poor adherence that would give me caution both TAF and TDF. You wouldn't want them to have to be taking PrEP sporadically as that could increase risk of HIV infections, but also potentially for resistance in HIV. Yeah, so BMI, CKD.

Interviewer: Okay great. And what are some questions and concerns the patients have raised about TAF or TDF? If you've had that experience.

Subject: Um one patient was terrified that TDF was going to lead to renal failure and dialysis and recalled her grandfather passing away from kidney disease unrelated to TAF or HIV or anything and she was absolutely terrified and in the office said: “I'm done with Truvada. I'm done. Give me something else. I'm terrified”. So that that was one patient encounter that I'll always remember.

Interviewer: Okay great um, and then for patients who had been switched or who've you've started on TAF what have their experiences been? Have the patients like did? Have they run into issues with it?

Subject: I think it's been really well tolerated, so no side effects. I think one patient the next time I saw them had gained five pounds, but I also don't know because it was the winter with and probably multifactorial with many reasons to have gained five pounds. [They’re] still taking TAF for PrEP and we're just keeping an eye on it and [there have been] no issues.

Interviewer: Okay great um and you mentioned one patient had been switched, and that was for kidney issues ?

Subject: yes

Interviewer: Okay, all right and how's their experience been since then?

Subject: Um, good I was supposed to have a check in with her last month. Um, but with the COVID pandemic that's sort of being pushed. But she hadn't had any issues when I checked in with her, so I think she's been doing fine.

Interviewer: Okay yeah great. And then just a few more questions here. How would the availability of generic Truvada but not Discovery influence your prescribing whereas Truvada is supposed to come out in a generic form and then relatively near future.

Subject: I think that's a great question. I think cost to patient is really important. I think if cost of brand TAF regimen was so much greater than generic TDF regimen and it was sort of a lower risk patient to have bone kidney or other side effects, potential side effects, from TDF then I’d opt for TDF. I think that based on insurance coverage if the cost was reasonable to the patient then I probably go for Descovy. Yeah.

Interviewer: okay great um and then just I kind of wrapping this up, are there any other experiences or thoughts that you have about TAF versus TDF containing regimens or thoughts about TAF versus TDF that we don't cover.

Subject: No. I think we covered a lot yeah.

Interviewer: Yeah I agree um, so thank you for participating. I appreciate it and this is a lot of really helpful information.

Subject: Great happy to participate.

Interviewer: Great, thank you.
