# Supplementary material for: Health care providers’ decision-making and early adoption of tenofovir alafenamide for HIV preexposure prophylaxis: An inductive qualitative study
Source: PLoS One. 2024 Dec 5;19(12):e0311591. doi: 10.1371/journal.pone.0311591 (PMC11620414; doi:10.1371/journal.pone.0311591)
Supplement: S1 File — (ZIP) [file pone.0311591.s001.zip › Clean transcripts/DedooseDoc_Participant 20 Transcript.docx]

I: I’m going to start by asking a few questions to learn what you have heard or what you know about using tenofovir disoproxil fumarate with emtricitabine (TDF/FTC) vs tenofovir alafenamide fumarate with emtricitabine (TAF/FTC) for PrEP. Have you heard about using TAF/FTC vs using TDF/FTC for PrEP before today?

S: Yes

I: Okay. And what have you heard about TAF/FTC vs TDF/FTC?

S: Um, I have heard that it appears, from some evidence, that they are, sort of equivalent in efficacy, or HIV-prevention, at least in the group of men who have sex with men. And so we should expect them to both work just as well, if people are adherent with them. Um, but they have different side effect profiles. And so with TDF I worry more about bone and kidney dysfunction, or toxicity, and TAF I worry more that they’ll gain a lot of weight.

I: Excellent. And then what are some of the sources of your information about using TAF/FTC vs TDF/FTC for PrEP? So some possibilities would be like colleagues, patients, pharmaceutical reps, advertising, journal articles, continuing medical education, online information or anything else.

S: Yup. I would say journal articles, and CME-type stuff, UpToDate, um, talks, HIV conference, Doug Krakower… came and gave a talk on this.

I: Yup

S: And I would say talking to colleagues, sure.

I: Excellent. And have you received any formal guidance or feedback from medical staff at your institution regarding TAF/FTC vs TDF/FTC for PrEP.

S: Um, I guess by way of conference discussions, if that qualifies, then yes.

I: Okay.

S: I definitely have been to at least one conference that was HIV focused where the discussion was, it was basically an hour on HIV prevention, and it mentioned you know, the different formulations of tenofovir, among other things, and kind of compared and contrasted.

I: Excellent. So then walk us through your thought process on how you’d make decisions regarding prescribing one or the other of these two PrEP options.

S: Yeah I would say that for me, um, I’m still pretty much prescribing Truvada, um, TDF form, because that’s what I’ve always done, and it’s what was out there first. And I feel like the number of patients that I’ve had on it, I think I’ve only had one patient so far that had any um, renal impairment, that has had any toxicity that I can measure from it. Despite having patients that have been on it for, you know, years, for prevention. And so I still feel pretty safe with it. I think that I do have one patient that I’m thinking about switching, and he would be my first one that I’ve put on TAF, um, Descovy, because he’s the one that’s developed some kidney impairment, that I don’t actually think he had before. And we’ve been, he’s had it for probably two years now, and it’s been very stable, um, but there’s really not another reason why he has it and so, I have wanted to talk to him. I ahd been talking to him previously before we realized that TAF was going to be okay, because he is a man who has sex with men. We’d been talking about taking on demand PrEP instead, to try to minimize exposure to his kidneys, but he had not been interested in doing that yet. I think he’s sexually active a little bit more frequently and so taking it sort of on demand didn’t seem like it was necessarily going to minimize his exposure to it so much. So we had decided not to do that.

I: That makes sense.

S: But otherwise, I don’t go right to TAF, because I don’t want them to gain a lot of weight. And I do worry about that a lot, because I’ve seen it a lot in my patients who I’m treating for HIV.

I: For HIV, do you tend to preferentially use TDF or TAF or does it just depend on…

S: For HIV, I would say at this point I generally use TAF-based, but then I know that those are patients for the most part that are going to be on it for the rest of their lives. And I definitely have some that I have switched off of TAF, and back to TDF, because they gained weight. And it didn’t improve when I switched their integrase inhibitor, which is, you know, the other one that I am usually blaming.

I: Sure. That makes sense. Uh, so then what specific factors would make you recommend TAF/FTC over TDF/FTC for PrEP.

S: I think I would recommend it, so if I had a patient who already had some impaired renal function, um, to start. Or if I had a patient that I knew had osteoporosis, or who had a lot of risk factors for osteoporosis, or a lot of risk factors for chronic kidney impairment, I think I would steer that person toward TAF.

I: Okay. And then what factors would make you recommend TDF/FTC over TAF/FTC?

S: Um, pretty much every… about everything else I guess, but I… you know…. Like definitely if I was worried about weight gain. So somebody who was already obese to begin with, or had a lot of metabolic syndrome issues maybe, because sometimes the cholesterol can rise a little bit on TAF, instead of tenofovir (TDF). I forget what activity it is, or if we even know what TDF causes that allows for kind of cholesterol to be a bit lower, versus what TAF, you know… how TAF is related to more elevated cholesterol, I don’t know. But maybe a metabolic syndrome. Um, or yeah, obesity.

I: Okay. And what about age?

S: Um, I don’t think I would use age necessarily as a cut-off one way or the other. I feel like age increases the risk of all of the above, and so, yeah, it’s not one that I usually think of as being a determinant.

I: Alright. What about any gender, other medical conditions, anything else?

S: Oh, well, I would say that I… you know, I don’t think TAF has been as well studied outside of the boundaries of men who have sex with men, and I think because we don’t know from evidence at least, that it prevents HIV as well in women, having vaginal intercourse, as TDF-based therapies. I probably wouldn’t use it for them.

I: That makes sense. And how do patient preferences come into play?

S: Uh, I… you know… unless there was a very clear toxicity concern on my part, I guess if the patient had a strong preference one way or the other, I would probably go with that, because there’s really not, you know.. I guess if they already had bad renal dysfunc…. Well know, because I’d be recommending they take TAF anyway. I guess one thing that’s interesting is that I have not yet necessarily sat with a patient who wanted to be on PrEP and said ‘we have this formulation of tenofovir and we have this formulation of tenofovir…. We use them in both the same way, do you have a preference based on the potential side effects’. Mostly I have said ‘I would recommend this drug, for this reason’, and then, you know, change that recommendation if needed. But I will also admit that I haven’t… you know… since the TAF became sort of better accepted and the evidence base became bigger, I don’t know… I haven’t had the conversation too many times, with patients recently, where we’ve gone back and forth about that.

I: That makes sense. How about any insurance or cost considerations when deciding between the two?

S: So I guess I would worry that TAF formulations might not be as well covered. I’ve never had a problem getting TDF, Truvada, covered for this purpose. I don’t know if the coverage is the same for TAF-based. So I’d worry that it wasn’t, but I don’t know.

I: Okay. And then, you’ve more or less already answered these, but just in case you have any other thoughts, what are some reasons or patient characteristics that would influence you to avoid a TAF-containing regimen?

S: Yup. That would be metabolic toxicity or obesity.

I: Great. And then, the same thing, so what are reasons or patient characteristics that would influence you to avoid a TDF-containing regimen?

S: Yup, and that would be kidney dysfunction or osteopenia or osteoporosis.

I: Okay. And what experiences have you had using TAF/FTC for PrEP?

S: Um, I have had a fair amount of experience using TAF/FTC (meant to be TDF/FTC) for PrEP, and it’s been mostly good experience. I feel like patients generally like it, don’t have much in the way of side effects, come in for their every 3 month kind of labs and HIV screening. And other than the one patient that I was telling you about that has had a little bit of an impairment in his renal function over time, no other problems.

I: Are you talking about TDF/FTC or TAF/FTC right now?

S: Oh sorry, did you say TAF? I was thinking TDF, sorry.

I: That’s okay!

S: So TAF I have not had experience.

I: Okay, have you had any patient inquiries or requests for TAF/FTC?

S: Mmm-mm

I: No. Okay. If you did have a patient come in and request TAF/FTC, how would you respond to that?

S: Uh, I would say… And this is someone who is on TDF right now? Or just someone who comes in never being treated and wants TAF?

I: Either way.

S: Okay. Um, so I guess I would say I would make sure it was the right patient, that it was a male patient, having sex with men. I would tell them that it’s an option, one of a couple of options right now. Um, and I would explain to them how you take it, and that the main side effects that we worry about is that there can be a fair amount of weight gain in some patients who take it… Not extreme amounts of weight gain, but on the order of, I don’t know… I actually don’t know… Probably like 5-10 pounds. And uh, and then I would let them know that there was an alternative, which was TDF-based, and similar to use. And that it did not carry the weight gain risk, but that it did carry some risk of kidney dysfunction, and bone thinning. And ask them, you know, think it through with them.

I: Makes sense. So then, for patients who come in and wish to be newly started on PrEP, do you tend to prescribe mostly TAF/FTC or TDF/FTC and why?

S: I would say still TDF/FTC, and I think because of… because I’ve had a lot of experiences with it that have been great, and low toxicity. Um, I do know that it’s covered by most insurance, and so I wouldn’t worry that they would be in the situation where they would go to try to get it and they couldn’t get it. And then the weight gain issue.

I: Great. And then, for patients who are already on PrEP, to what extent, if at all, are you switching from TDF to TAF?

S: Yeah. So there I’m only doing it if somebody… if it looks like somebody has developed toxicity. Or they come and they’re worried because they’ve heard…. You know there’s a lot of…. I think there are a lot of class action suits out there now against TDF, and so I feel like people are more aware of it than they used to be, that there might be some kidney toxicity. Um, certainly my HIV patients are more aware of it, and so I think if they came and asked about that… yeah. I would definitely consider switching.

I: Makes sense. My next question was what are some questions or concerns that your patients have raised regarding TAF/FTC, and then regarding TDF/FTC?

S: Yeah… Yeah. I feel like the TDF… Word is out on TDF for sure. TAF, I don’t feel like word is out as much. I don’t feel like my patients know that there are downsides to TAF. But again, I don’t have that many that take it for that reason. Most of my patients that take it are taking it for their HIV disease that they already have, and in that setting, word…. I don’t think word is out. At least, among my patients.

I: Makes sense. Have there been any patient concerns about effectiveness for either of them?

S: No

I: Side effects? Any particular side effects?

S: Nope

I: What about patient concerns about insurance coverage, out of pocket costs or pill size?

S: They ask about insurance coverage, but no. I haven’t had a situation yet, at least with the TDF, where they’ve notified me that their insurance didn’t cover it….What was the last question?

I: If anyone has been concerned about pill size?

S: Oh. No. I would say no. I haven’t had anybody complain about the Truvada pill size./

I: Okay. And then, do you have any patients… for patients who have been switched to TDF/FTC, if applicable, how has their experience been?

S: Yeah. I haven’t had anybody switch yet.

I: Okay. Um, and you said you haven’t had any new starts of TAF/FTC either, right?

S: Right.

I: Okay. Um, and that next one also doesn’t apply… So then, how if at all would the availability… er… how if at all does the availability of generic TDF/FTC but not TAF/FTC influence your prescribing.

S: Um. It would influence it, if insurance wasn’t covering either brand name Truvada or Descovy. But other than that I don’t know that it will influence it very much.

I: Okay. Any other experiences or thoughts that you have about TAF/FTC containing regimens that you would like to discuss?

S: I don’t think so. No, I think we’ve covered it.

I: That was the end of our initial questions, although we’ve tacked on a couple of COVID questions, because, you know… yeah. Why not. So our first COVID question is as a prescriber, have you noticed any influences of the COVID pandemic on your prescribing of PrEP?

S: Hmm. Um, no. I don’t think so.

I: What about from a patient perspective? Have you had any patients tell you about any influence that the COVID pandemic has had on their PrEP usage, uptake, etc?

S: Well I definitely have had many patients come in and tell me that they’re not as sexually active as they were before, because of COVID. Um, we haven’t linked that directly to refills on their PrEP prescriptions, but I definitely have heard that story.

I: Has anyone mad any changes, like stopped taking it because they’re less sexually active, or self-discontinued, or anything like that?

S: So, um, I would say not as a direct result of COVID, no. I definitely have patients that have come in to me, you know, pre-COVID and said, ‘I’ve decided to stop it because I’m not as sexually active as I used to be’. But not during COVID, I haven’t had that experience.

I: Okay. Great. Any other thoughts about COVID and PrEP?

S: Mmm-mmmm

I: Excellent. That’s all the questions that I have.

STOPPED AT 11:58
