# Supplementary material for: Health care providers’ decision-making and early adoption of tenofovir alafenamide for HIV preexposure prophylaxis: An inductive qualitative study
Source: PLoS One. 2024 Dec 5;19(12):e0311591. doi: 10.1371/journal.pone.0311591 (PMC11620414; doi:10.1371/journal.pone.0311591)
Supplement: S1 File — (ZIP) [file pone.0311591.s001.zip › Clean transcripts/DedooseDoc_Participant 21 Transcript.docx]

I: I’m going to start by asking a few questions to learn what you have heard or what you know about using tenofovir disoproxil fumarate with emtricitabine (TDF/FTC) vs tenofovir alafenamide emtricitabine (TAF/FTC) for PrEP. So have you heard about using TDF/FTC vs TAF/FTC for PrEP before today?

S: Yes I have.

I: Okay. Uh, and what have you heard about TAF vs TDF?

S: Um, I’d say that they’re fairly equivalent to each other. There’s not necessarily a huge benefit in terms of protecting against HIV between the two. I think the consideration for TAF over TDF is if you think someone may have more high risk features for some of the toxicities associated with TDF, such as renal insufficiency. That’s probably the biggest thing I would be thinking about between the two. I think TAF as well has really only been approved for men who have sex with men. There haven’t necessarily been studies in cis-female patients, at least up until this point.

I: Great. And what are some of your sources of information about using TAF/FTC vs using TDF/FTC?

S: Um... good question. I certainly go to the CDC for some of that guidance. They have PrEP guidelines that are there. Um, you know, through our own conferences, such as HIV conference, we’ve talked about PrEP in that setting. Um, and then I will say I help to manage the HIV update course, so I get to hear Doug Krakower talk about it every year.

I: I’d classify that one as maybe “colleagues”.

S: Right, sorry. Perhaps outing myself on this interview here.

I: I mean, I’m doing these with all people at BI and Fenway, so I think everyone knows Doug, so you’re okay there.

S: There we go.

I: Okay great, and then have you received any guidance or feedback from your institution regarding the use of TAF/FTC vs TDF/FTC?

S: Um...

I: And I know you work both at BI and Fenway so either way.

S: Yeah. I guess, I don’t think I’ve necessarily gotten anything from BI. At Fenway there are some, kind of smaller announcements, again, just kind of thinking about how you might think about using TAF vs TDF, and then I think kind of also wrapped into that is the fact that TDF/FTC is now generic. And maybe more preferred, or prioritized by insurance companies.

I: Okay, great. So then, walk us through your thought process on how you would make decisions deciding whether to prescribe one or the other of these two PrEP regimens.

S: Um, I think that, for the most part, I don’t know that I’ve embraced TAF as much per se, I think I’m still kind of thinking about TDF/FTC as kind of the primary drug of choice. And part of that is probably just having started with that drug, as the more likely agent to grab for PrEP management. Um, I will say that I have had a couple of patients who have had other comorbid illnesses, so things like diabetes, hypertension, coronary artery disease, or are maybe on the older side, so for instance I have a patient who is 76 and on PrEP, and I will think a little bit more about a TAF-based agent in that kind of situation, where I’m a little bit more worried about the renal toxicities, for instance. One patient I did switch to a TAF-based regimen, because they did end up having some increased renal injury as a result of the initial TDF use. Um, so I guess I... when I’m thinking about it, it’s more about, kind of what is it that they are going to be able to tolerate. What is accessible to them, as well, in terms of what the cost will be, or insurance coverage will look like. And then, maybe a little bit less impactful, but something that I will sometimes consider – how I’m prescribing PrEP. So you know, if it’s kind of a short course, kind of involved in, like they’re travelling somewhere else and they think they’re going to be sexually active during that period of time, or thinking about PrEP as a short term way of protecting them against HIV, or on demand PrEP, for instance, then I’ll think about TDF in that situation.

I: Makes sense. You’ve already mostly answered these next questions, but what are specific factors that would make you recommend TAF/FTC over TDF/FTC?

S: Yeah, I think again, knowing that we potentially have a longer track record with that medication ... who is it that I’m prescribing it to as well, so kind of male, or cis-female identified. And then cost, of the drug, may be a factor, and then any sort of comorbidities that may increase their risk of renal toxicities.

I: And that would be for TDF?

S: Yes.

I: And then what factors would make you recommend TAF over TDF?

S: Um...

I: If any

S: Yeah. Uh, I guess the opposite. To some degree, you know I guess again, if they have... if they already have established renal dysfunction, or those comorbidities that would increase the risk. And then I probably would be a little bit less likely to prescribe in cis-female, though, again, I think if there were comorbidities there that might increase their risks of renal side effects with TDF, you know there would be a consideration, but it’s kind of weighing the risk/benefit in patient preference in that situation.

I: And then those risk factors for renal dysfunction that you’re looking for.? I think you mentioned diabetes...

S: Diabetes, hypertension, as well. You know, if they’re on a lot of other medications that may also impact renal function, that may be something else to consider.

I: Sure, that makes sense. Um, okay, and then: insurance and cost considerations. How would those affect your... and again, I think you’ve already talked about this a little bit, but how would those affect your decision making?

S: I think I would probably go with the drug that’s going to be covered, as well. And then you know, if they do run into any sort of complications or challenges, that might be enough of a prompt to be able to make a switch and get some assistance from insurance company.

I: And at this point, which of the two are you finding easier to get covered?

S: The TDF.

I: The TDF, okay.

S: Yeah.

I: Alright. And then, are there any reasons or patient characteristics that would influence you to completely avoid a TAF-containing regimen? Like absolute contraindications?

S: I don’t think so.

I: What about a TDF-containing regimen?

S: Um... I think it really would be kind of the renal insufficiency piece, yeah.

I: Okay. And then, what experiences have you had actually using TAF/FTC for PrEP?

S: Um, I’ve had a little bit of experience with that. Again, I have a couple of patients – one where they did have some renal injury, as a result of TDF, and so we switched to TAF. And then another patient who, because there may be some increased risk, and we were anticipating that they would be using this medication longer term, we decided to use TAF in that situation. I actually... I also... I didn’t think about this before but I also have a small group of patients who have Hepatitis B, and need to prevent HIV infection, and so sometimes that is a consideration as well. Because these are patients where I would think about a tenofovir based regimen, more long term, to also kind of manage their Hepatitis B in addition to PrEP. There, though, I still have been using TDF, I’d say more frequently again, and that is probably more based on the fact that the insurance coverage is a little bit more complete in that situation.

I: That makes sense. And then, approximately how many patients do you have on your panel who are on TAF/FTC for PrEP?

S: Um, I would say probably 6 to 8, or so at least at this time.

I: Okay. And then, what, for those patients, what were the factors that influenced your decision to prescribe a TAF containing regimen?

S: Um, again I think it was because of kind of renal insufficiency, or the risk of.

I: Makes sense. Have there been um... have you had patients who were both newly started on PrEP and were started on TAF/FTC as well as patients who were switched from TDF/FTC to TAF/FTC?

S: So one patient who I switched from TDF to TAF, and then... I’m trying to think. I think I only have one other patient on TAF, that we started right away, as a new start to PrEP.

I: And what were the factors for the new start?

S: Um, there it was due to comorbidities – diabetes, and already had some proteinuria.

I: Sure. Makes sense. Um, so then for patients who wish to be newly started on PrEP, would you tend to prescribe mostly TAF/FTC or TDF/FTC and why?

S: I think I’m probably more likely to prescribe TDF, and I think there just given a longer experience with that particular medicine, the knowledge that it’s generic now, and kind of less costly, compared to brand name TAF, would be the bigger pieces.

I: Okay. Um, and then the next question I think you’ve pretty much already answered, which is, for patients on PrEP, to what extent, if at all, are you switching from TAF to TDF... Sorry, sorry. From TDF to TAF? And you had talked about that one patient...

S: Yeah, it’s pretty infrequent, I’d say, but um, yes. For the reasons that I had kind of listed before in terms of their worsened potential side effects while on TDF.

I: Makes sense. And then any questions or concerns that your patients have raised regarding TAF/FTC?

S: Um... No actually. I don’t think I’ve had any patients concerned. It’s often times the other way around, where they do worry about the side effects of TDF, and just kind of having to discuss a little bit more in terms of what their preference is, or you know, what might be limitations to accessing TAF if there is a strong desire for that.

I: Tell me more about the concerns that patients have had about TDF?

S: Um, again I think it’s kind of long term side effects of the medication, knowing about things like bone thinning, for instance, or the kidney changes.

I: Okay. Then how does that patient preference/patient request come into play? How does that conversation play out?

S: Um, well I think it comes about certainly in terms of discussing what the options are for PrEP right now. And then what the kind of risk benefit is being on one medicine vs the other. But also kind of with the knowledge that TDF is often times preferred by insurance, again, now that it’s generic. That tends to be kind of the flip in terms of if you’re prescribing brand name Truvada, there’s the option to potentially have the generic there as well.

I: And for those patients that have come in with concerns about side effects from TDF or requests for TAF, what ultimately ended up happening in those situations?

S: Um, so it... again kind of with the one particular patient where we did make a switch, I did have to submit a request to the insurance, to kind of give a reason as to why we were preferring a TAF based regimen over a TDF based regimen. I think with other patients, to be honest I don’t know that... I haven’t necessarily seen a lot of the other kind of side effects be impactful over a very long period of time. Kind of my experience has been for many patients, unless they’re kind of with Hepatitis B, that they kind of intermittently are on PrEP for a period of time, and then maybe go off for a period of time and come back on. So I don’t know that I’ve had the experience of really seeing someone who’s really kind of more longitudinally on the medication.

I: Sure. That makes sense. So then, for the patient... You said you had one patient who was switched from TDF/FTC to TAF/FTC. How was their experience?

S: They were totally fine with it. I think... We actually had stopped TDF... or stopped it, this was kind of prior to TAF being approved for PrEP. So there was a period of time where he was on no PrEP, essentially. We were also kind of monitoring his renal function to see if that would stabilize or improve after stopping the TDF-based regimen. And he did. He regained some of that renal function. He was able to have other kinds of methods of safe sex practices, but was certainly interested once TAF was approved for PrEP, to be able to use that as well. So I think that transition ended up being totally fine, and he was happy on TDF, and he was happy on TAF.

I: Great. And how has the experience been for the patients who were newly started on TAF?

S: I think so far so good. You know, again, we’re... I’d say that there hasn’t necessarily been symptomology that’s been concerning to patients. They’ve been tolerating it well. And then, you know, with kind of routing bloodwork we’re just assessing for any kind of changes that you might pick up on bloodwork, or urinalysis testing, for instance, looking for proteinuria. And I haven’t seen anything there.

I: And, for either PrEP regimen, any particularly positive or negative experiences that patients have had with their PrEP regimens?

S: Not with the regimens themselves. Again, I think sometimes there have been access issues or copay costs that have been challenging. And so that piece of it has been difficult for some patients.

I: Okay. Makes sense. And that seems to be nowadays more of a problem with TAF then TDF?

S: Correct.

I: Makes sense. So when you have a new patient who comes in asking about PrEP, do you tend to present both options and talk them through equally, or do you tend to kind of just recommend TDF/FTC.

S: I do generally talk about both as an option, though I imagine that I tend to stress the TDF more, yeah.

I: Makes sense, makes sense. And then have you had any patients who’ve switched from TDF to TAF and then switched back to TDF?

S: I have not.

I: Okay.... Very specific question.

S: Yeah.

I: And then we’ve talked about this a bit, all the way through, but how does the availability of generic TDF, but not TAF, influence your prescribing?

S: I think it influences my prescribing, yeah. Because I think again, trying to get patients a regimen that’s going to be protective for them is really important. And if that means going with an option that is excellent but, you know, has these potential side effects, but is also more accessible, then I’m certainly going to go for that, yeah.

I: Makes sense. Any thoughts or experiences about TAF/FTC containing regimens you want to discuss?

S: Um, I don’t think so.

I: Great. So that was the end of our TAF/TDF piece. We’ve tacked on a couple of COVID questions, because... we’re here.

S: Yeah.

I: So the first is, from a like prescribing standpoint, have you noticed any effects that the COVID pandemic has had on your prescribing of PrEP?

S: Um, hopefully not, in the sense of I want to make sure patients are still having good access to it. And, you know I think the monitoring that has gone along with PrEP has probably not been as consistent. Because people are remote, or worried about coming in. It’s been a little bit more challenging to have people come in to do STI testing, or bloodwork. But I think ultimately, you know, trying to maintain the medication, if they’re using it, is important. And so at least at Fenway, one of the things that they’ve done is actually kind of automatically refilled those prescriptions when they come up. And so there are pharm techs and nurses who are going ahead and kind of approving the fill even before it goes to the sign... goes to the provider for signature. Just to make sure that there’s continuity and no breaks in receiving the treatment.

I: Makes sense. And then, the other half of the question is from a patient perspective, have you had any patients tell you about any effects that the COVID pandemic has had on their PrEP usage, risk factors, etc?

S: Yeah I’ve had a couple of patients where they’ve stopped PrEP because they’re not necessarily engaging in sex, essentially, during the pandemic. Or they’re, you know, having less outside partners. Um, so I think that there has been an impact in terms of some of that sexual activity, with multiple partners or... yeah.

I: Okay. Great. Any other thoughts about the COVID pandemic and PrEP?

S: Um... No. I mean, I think the pandemic has shown us what we can do wihtout having an actual in person visit.

I: Yeah. That is true.

S: Um, yes. And yeah. I think just the ability to try to continue patient services despite, you know, some of these roadblocks.

I: All right.
