# Supplementary material for: Health care providers’ decision-making and early adoption of tenofovir alafenamide for HIV preexposure prophylaxis: An inductive qualitative study
Source: PLoS One. 2024 Dec 5;19(12):e0311591. doi: 10.1371/journal.pone.0311591 (PMC11620414; doi:10.1371/journal.pone.0311591)
Supplement: S1 File — (ZIP) [file pone.0311591.s001.zip › Clean transcripts/DedooseDoc_Participant 3 Transcript.docx]

Subject 03

Interviewer: Alrighty and where is my record button? Perfect. There we go. All right so we should be recording, which we are. So I just went over the consent with you and we will get started. So I'm gonna ask you a few questions to learn what you have heard or know about using two different formulations of PrEP that's Tenofovir Disoproxil Fumarate with emtricitabine versus Tenofovir Alafenamide Fumarate with emtricitabine for PrEP. So, have you heard about these two different formulations before today?

Subject: Yes.

Interviewer: Okay great. And what have you heard, just in general. Feel free to expand on wherever you want to about TAF, which is Tenofovir Alafenamide versus TDF, the Tenofovir Disoproxil formulations for PrEP. Just in general things that you've heard about both.

Subject: So I know that alafenamide believe was approved many FDA, I believe a few years ago now, and there’s a new one which I think is the Disoproxil. Yeah, I think the Disoproxil is the new; the that was recently prescribed. Or is it the reverse?

Interviewer: It’s the reverse of that.

Subject: Okay, yeah the reverse. So the Disoproxil was a few years and the alafenamide was more recently for Pre-exposure prophylaxis. I think in terms of like the indications I think the like classic indication was and I think what all the like primary literature for Disoproxil was in men who have sex with men. Men who have sex with men who are HIV negative and in sero-discordant relationships or have multiple partners. And that I think, at least for a while, it was my recollection that that was really the only group that kind of had that approval. And then I think for Disoproxil, I think it was expanded to start including women but I know that the alafenamide is not currently approved for individuals who were born genetically female. At least that's as I recall what the like commercials that are all over TV remind. And I believe that the alafenamide might at least as I recall, maybe a little bit may have a little bit less renal toxicity compared to the Disoproxil, because I know that that was like a big concern even though it's like largely thought to be reversible and then I know the other side effect at least with the Disoproxil is like bone health… like bone resorption and osteoporosis and all that stuff. And again, that's something that I believe is not an irreversible thing. When you stop it gets better. Okay. I think that's like pretty decent summary. Yeah.

Interviewer: Yeah! Now that was great. That was great. Just so we get a sense of where people are getting information from, where would you say you've learned the most about the new formulation of PrEP as well as PrEP in general? And it can be colleagues, patients, pharm reps, advertising, and journals, just as some examples.

Subject: I would say that under a general circumstance, I would say colleagues, lectures during residency. How are these lectures during residency? I actually participated. I like helped out, as I believe that Fenway Institute was doing a study looking at, kind of similarly, at like providers and like how they would do that. And they actually like did a study looking at like patient like preferences or patient understanding of what PrEP is, and I was one of the providers that helped out with that.

Interviewer: Oh awesome.

Subject: So, I got some knowledge information from that. In terms of the Alafenamide specifically, since that's a newer I don't know that I like ever. I think it was probably like a couple colleagues and then now they're like commercials all over television that mention Alafenamide or like Descovy for PrEP. So there are commercials on TV now that are quite ubiquitous… like I would say I've seen like them multiple times.

Interviewer: Okay, yeah, they are all over the place. Which I've learned since the quarantine. And then, have you ever gotten any guidance or feedback from medical staff at BI or anywhere else who practiced about the differences between the two and when you would use one versus the other. So I guess to rephrase what I’m asking, have you gotten any guidelines or guidance from your clinic about the use of these?

Subject: About choosing one versus the other note nothing specific

Interviewer: okay, all right and then about in in general about PrEP, does your clinic or residency program have any guidelines or informational sessions that help guide you in starting somebody on PrEP or what to do when you inherit a patient who and your panel who is on PrEP?

Subject: I would say, no. I would say that there are certain providers that HCA that prescribe it more frequently than others. I think that I'm probably a little more unique in that since I had that experience with that other study, I would say I probably feel at least maybe a little more comfortable just with like the monitoring that's required, but I haven't had any specific like information sessions about it or like handouts or like processes to go through or anything like that. It's kind of just like what I what information I know.

Interviewer: Okay. All right. And then just in terms of when you're in clinic and say you had a patient come to you asking to start on PrEP, are there any factors that you would take in starting one over the other in terms of the Alafenamide over the Disoproxil, or is that not something that's generally addressed in your clinic?

Subject: That's not something that I have had to address. It's not something that I know I would that I have enough knowledge about, exactly other than I think like the renal thing. Like if someone maybe had a family history of like CKD or like some kind of predisposition, I might say ‘I guess maybe the Alafenamide’, but like other than that, I have really no okay conception of when I could choose one or the other.

Interviewer: okay and based on any of the resources you've heard of or read, have you heard of any of adverse effects of the Alafenamide?

Subject: I mean, I think there is still the like kind of renal… like there can be changes in creatinine. I don't know, but I don't think that there's as much about bone. But like other than that there's nothing that I can recall specifically. No nothing that I can remember.

Interviewer: Okay yeah that's perfect. And then um, in terms of patients that have come in, have you had any patients ask about the differences between the two or? Or ask to be started on one versus the other or ask about changing.

Subject: I don't know.

Interviewer: Okay, all right. And then… sorry give me one second here. So in general if a patient were to ask you about the differences between the two or you're preferences on them, where would you go to get information for patients?

Subject: I can’t say for sure that there is one place, but I am sure that like the or the like just like general national by CDC and those organizations. NIH probably have like guides for providers and decision tools out there. I can't say for sure because I haven't used any of them, but if I have like a question or something like that, that's probably where I would go to…is like society society guidelines and things like that.

Interviewer: Okay, all right, great. There’s some more emerging data that shows Tenofovir Alafenamide, the newer component, could be associated with some weight gain and lipid abnormalities. That, taken in conjunction with what we already talked about with the Disoproxil form with the renal and bone issues; knowing that would that change your prescribing practices if you were to have a patient come in asking to start on PrEP?

Subject: I think it would I think it would depend on individual factors. I think if someone was overweight or obese or had family history, if they were older and had lipids checked and they were already borderline or mildly elevated, it might make me move towards the Disoproxil formulation. Just considering if this is something that they're gonna be on long term or that they're gonna want to take long term, especially considering that the renal impairment and the bone changes with the disoproxil form are reversible. In terms of the lipid stuff, my guess is it would probably be somewhat reversible with stopping. But, I mean weight gain is also weight gain and It's hard for people to lose weight, so I would probably like obviously use it in my like decision making in my head of like this one might be a better option versus the other.

Interviewer: okay great yeah. And like I said, those are some emerging studies and everything on came out. But, that's just something else that people have to bring up with the use of PrEP and the Tenofovir Alafenamide version. In terms of… and you said you haven't had any patients on PrEP correct? Okay, and this can be more general too, but if a patient to asking be switched from one to the other what role would patient preferences play into your decision making if you had a new patient who is already on the Disoproxil form?

Subject: I am not sure. I mean I don’t think I would necessarily have a problem with that unless there was like a good reason for me to like think that there might be an issue. But I mean, they're basically like slightly different formulations of the same two drugs. I think that the Tenofovir is basically the same and the emtricitabine is still the same. I guess it’s just like side chains or whatever. It’s just the formulation of the Tenofovir that is slightly different, so I don't think I would have much of an issue with switching. I think that insurance sometimes is kind of something to keep in mind and with all of them. I think with Alafenamide being newer, I think insurance companies… there's probably been a bit of a lag. I think most are starting to cover Disoproxil by now since it's been a few years since the Alabama's newer. I think that's something that I believe I recall hearing like, “oh like getting an insurance approval for that is much tougher”.

Interviewer: Yeah and so right now with the insurance costs and everything, both drugs are priced pretty similarly, um, you know pennies difference. However, starting next year the Disoproxil form will become generic. Will that influence how you would think about counseling patients and prescribing?

Subject: I mean, I think it's definitely gonna be important. I mean, I'm gonna be going to an institution that cares for prey very predominantly underserved population where cost of medication and is a bigger deal and I think that sometimes if you're like talking about like moving to a generic that's gonna be equally as effective and is gonna be significantly cheaper, I think it's a great thing for patients, especially those in an underserved population. And, I know that a lot of the… I mean I think that with HIV numbers are moving in the right direction for a lot of demographics, with the exception of I believe it's African-American and Latino men who have sex with men, which I like obviously don't want to like stereotype or anything, but I think there's a large portion in that group that probably has some degree of socioeconomic and economic instability, so I think that having an option that is generic and is gonna be significantly cheaper is a good thing. It would be something I would consider and counseling someone.

Interviewer: Okay, great. Great. That's a helpful framer reference to have. So those are a lot of the big questions I wanted to ask. In terms of in general in your clinic, is there somebody that, in terms of a preceptor, that tends to prescribe more PrEP or HIV medications that you would have access to if you had questions about a patient.

Subject: Um, yeah, they're definitely providers at HCA , healthcare associates where I am, that like I know like prescribe significantly more frequently. I can think of like at least two off the top of my head and I think my direct preceptor, while she I don't think she necessarily prescribes PrEP all that frequently, I think she is pretty well versed in it, so she could help me with like co-managing and labs and things like that. But yeah, they're definitely people I would feel comfortable talking to about it.

Interviewer: So, are there any other experiences or thoughts that you've had about patients who are on PrEP or about these new regimens like the Alafenamide version of Tenofovir on that you want to bring up or discuss?

Subject: I mean, I don't think it's anything specific. Just generally I still think that there's not a ton of education being given on like how to prescribe it and what you need to know about each of the regimes. I don't think there's a ton that's being done in terms of educating house staff or trainees about it. I also think that there's a pretty big gap even in the primary care world. I can think of one situation where there was an attending where I was with who was significantly, obviously, more senior than I was who had never even prescribe PrEP or even thought about prescribing it and there was a patient that was asking about it and he [the provider] was asking trainees about their experiences with it. So I think there's still a pretty big gap, and I think it might be a generational gap between the current generation of trainees and like newer providers who are used to this versus providers who are not infectious disease trained who may have trained some years ago. So this is all new, so I think there's still a ways to go. And also stigma is obviously something to continue to address because I think a lot of patients probably feel somewhat stigmatized when discussing something like this.

Interviewer: Yeah, yeah absolutely. And just one more question really. What are some resources that you think would be helpful for trainees…everyone from med students through fellows and beyond? What are some resources you think might be helpful?

Subject: I think that it really kind of matters and I really try to think about like how individuals like in trainees learn and I think it's different for everyone. I'm someone who finds handouts to be things that I'll probably just throw away because it's a piece of paper and kind of a waste of space. I think things like badge buddies I think can sometimes be helpful if it's something that you think you're gonna be doing more frequently. I do think while lectures are not the most effective way to transit information, but I do think that it can atleast can start the conversation. And, I think small group activities small group work and I think really just trying at least from a trainees perspective. I think just like doing our best. I think continuity is important but I think also diversifying patients or providers kind of panel experiences will lead to more comfort such that we don't run into this situation where there's only a small handful of primary care providers that feel comfortable prescribing or that have experience prescribing these medications. I think it's about getting as many people comfortable with this as possible.

Interviewer: Okay great, um, so those are all the questions I had for you. Do you have any questions for me?

Subject: I don't think so.
