# Supplementary material for: Health care providers’ decision-making and early adoption of tenofovir alafenamide for HIV preexposure prophylaxis: An inductive qualitative study
Source: PLoS One. 2024 Dec 5;19(12):e0311591. doi: 10.1371/journal.pone.0311591 (PMC11620414; doi:10.1371/journal.pone.0311591)
Supplement: S1 File — (ZIP) [file pone.0311591.s001.zip › Clean transcripts/DedooseDoc_Participant 4 Transcript.docx]

[INTERVIEWER]: Do I have your permission to record this interview?

[PARTICIPANT]: Yes.

[I]: Once again, I’m encouraging you to ask for clarification, if there are any questions that aren’t clear or express any concerns you might have during the course of the interview. I also want to remind you that you can refrain from answering any questions if you are uncomfortable and I will just proceed to the next question and you can also terminate participation in this study at any time.

[I]: Thank you for agreeing to participate in this interview. Our research team needs your help to learn more about how healthcare providers make decisions about available PrEP options and early patterns of prescribing behaviors by providers.

PrEP stands for “Pre-Exposure Prophylaxis”, the use of any medicine to prevent a disease before exposure to that disease. This most commonly refers to the use of antiretroviral treatments every day by people at high risk of exposure to HIV, in an attempt to prevent HIV infection. Consequently, for this interview, we define PrEP as the use of any medication taken before having sex with the expectation of getting protection against HIV infection. Today, we’d like to discuss two antiretroviral PrEP regimens.

It is important to remember that the questions I will ask do not have a right or wrong answer and therefore your answers are not going to be evaluated as right or wrong. What is most important is to tell me what you think, and not what you think I may want to hear. I am here to listen and learn from you. Please try to be as open and honest as possible so we can learn from your thoughts and opinions.

Do you have any questions or concerns before we get started with the interview?

[P]: No.

[I]: I am going to ask you a few questions to learn what you have heard or know about using tenofovir disoproxil fumarate with emtricitabine (TDF/FTC) vs. tenofovir alafenamide fumarate with emtricitabine (TAF/FTC) for PrEP. Have you heard about using TAF/FTC vs. TDF/FTC for PrEP before today?

[P]: So, I’ve thought about it before and I’ve explicitly kind of raised this question to my faculty preceptor previously. My understanding, at least the last time we had this conversation is that TDF/FTC is technically the one that is studied for this specific indication of PrEP, and that TAF/FTC while having better [inaudible] side effects, like renal protection, bone protection is not technically studied for PrEP and therefore I was encouraged to continue prescribing TDF/FTC if I was considering it.

[I]: What are some of the sources of information about using TAF/FTC vs. TDF/FTC for PrEP?

[P]: I guess my faculty preceptor. I think the context in which this has arisen previously has been looking at the US PTF EPSS, like the preventive task force suggestions. I don’t think I looked very further much into if after that. I think I looked at “Up to Date” very briefly, but I don’t think I looked at IDSA guidelines.

[I]: Have you gotten any of your information from colleagues, patients, pharmaceutical reps or advertising?

[P]: I’m a lowly resident, I don’t think I have been advertised to.

[I]: How about journal articles, CME, online information, or anything beyond what you’ve already said?

[P]: Not really.

[I]: Have you received any guidance or feedback from medical staff at your institution regarding the use of TAF/FTC vs. TDF/FTC for PrEP?

[P]: Only the aforementioned that it was not technically [inaudible].

[I]: Walk us through your thought process on how you make decisions regarding prescribing one or the other of these 2 PrEP options.

[P]: So I actually have not prescribed very much PrEP, I think maybe only to one or two patients and typically this comes up for me, like you might have a patient at a primary care clinic for their annual physical type visit where I’m asking screening questions surrounding someone’s risk factors particularly with sexual transmission as well as if someone is a person who injects drugs – that’s kind of where this question is occurring. I have sometimes raised this when I am establishing a new patient as well who I know is [inaudible], I also see a lot of patients who are in the residential houses for addiction treatment on campus. Sometimes that comes up as well. There have been some barriers surrounding prescribing PrEP to people regardless, with regard to the number of other clinical issues that arise as well in care and what feels more immediate and pressing and what could be tabled to another time perhaps in terms of triage. And then finally, when at that point of deciding between TAF and TDF, I think ideally, I would like to be prescribing TAF because it seems like the side effects are much better, and I think we know for other indications that it’s a better drug, essentially or equivalent than TDF. I think ideally, I would be prescribing TAF but have really only prescribed TDF/FTC.

[I]: What specific factors make you recommend TAF/FTC over TDF/FTC?

[P]: I think especially the renal protection and the bone protection piece.

[I]: How about TDF/FTC over TAF/FTC?

[P]: I guess just technically what the FDA approvals are for.

[I]: How do patient preferences come into play?

[P]: I don’t think many of the patients that I’ve talked about this with have had specific preferences. I imagine that, I know back like 5 or 10 years ago there was a lot of stigma surrounding being a “Truvada whore” so I feel like some people might have negative associations with TDF/FTC but that’s not something that I’ve encountered. I think especially because the population that I’m primarily working with is more people who are injecting drugs and not necessarily people who are engaged in those higher risk sexual encounters.

[I]: Are there any patient characteristics like gender, or medical conditions, insurance or cost considerations?

[P]: Not really, just more like I mentioned the sense of triage for people who may be at higher risk in general with a lot of different medical conditions, and just trying to figure out which think I need to work with them on first and which things to be tabled to another appointments. But then again, if someone is able to come to a follow-up appointment or not is obviously a huge barrier too.

[I]: What are some reasons/patient characteristics that would influence you to avoid a TAF-containing regimen?

[P]: You know, I’m not really sure because I haven’t prescribed it very much.

[I]: And reasons/patient characteristics that would influence you to avoid a TDF-containing regimen?

[P]: Only perhaps stigma. And I guess maybe stigma surrounding the original like TAF/FTC. Like if a patient has a very strong preference for TDF/FTC and after some conversation about the risks and benefits of TAF vs TDF and they’re still very much on the TDF train, then, sure [I would prescribe TDF over TAF].

[I]: What experiences have you had with using TAF/FTC for PrEP?

[P]: Not very many, like I mentioned.

[I]: Do you have any patients on your panel on TAF/FTC for PrEP?

[P]: No.

[I]: Tell us about any patient inquiries or requests for TAF/FTC PrEP. (If none, then ask about how they would respond to an inquiry or request for TAF/FTC.)

[P]: I don’t think the patients that I’m speaking with about PrEP have a great sense of the nuance between TDF and TAF, so they haven’t specifically asked.

[I]: If you did have a patient inquiry or request for TAF/FTC how would you respond?

[P]: I think that I would commend them first for saying that they’re interested in PrEP in general and opening that line of communication with me. I would explain that, assuming that FDA approvals haven’t changed in the last few months or so, that technically speaking TAF is not FDA approved for this specific indication but it is the equivalent in other indications especially in the treatment of HIV so that I would recommend given b etter bone and renal protection to try the TAF, but again with the caveat and understanding that this is not technically FDA approved.

[I]: What are reasons you have not or wouldn’t start a patient on TAF?

[P]: I feel like I need to look back also on whether or not anything surrounding resistance, patterns, that sort of stuff, what kinds of encounters they’re engaging in and how frequently, maybe that’s something that would influence my decision but I haven’t run into that frequently enough to feel particularly strongly about it. [Inaudible] better regimens for treatment of HIV anyway so I’m not sure it would actually influence me that much.

[I]: What are potential benefits and potential risks that you weigh when deciding to prescribe TAF vs. TDF containing regimens?

[P]: I think just the bone and kidney stuff.

[I]: For patients who wish to be newly started on PrEP, do you tend to prescribe mostly TAF/FTC or TDF/FTC? Why?

[P]: [Inaudible] receptor preference for prescribing TDF.

[I]: For patients on PrEP, to what extent, if at all, are you switching patients to TAF from TDF containing regimens? Why?

[P]: I have not actually inherited people who were on PrEP as a preexisting part of their medical care, so it’s really just been starting people on TDF.

[I]: What are some questions/concerns that your patients have raised regarding TAF/FTC?

[P]: I think they haven’t really raised a ton, honestly. I think that at the juncture I am meeting a lot of patients at for this who are perhaps candidates for this are like “I need to turn my life around right now and I’m going to do whatever sounds good right now” which obviously has its plusses and minuses as well.

[I]: What are some questions/concerns that your patients have raised regarding TDF/FTC?

[P]: Same thing.

[I]: Has anybody raised any concerns about effectiveness, side effects, insurance coverage, OOP costs or pill size?

[P]: I think people have probably mentioned or asked about effectiveness and price, though I think through Dimmock the price doesn’t wind up being so much of an issue. And then the effectiveness piece I think we’ve kind of talked about how exactly you’re taking it and stuff like that.

[I]: For patients who have been switched from TDF/FTC to TAF/FTC, how has their experience been? But I think you said you haven’t had any of those, right?

[P]: Right.

[I]: How about those who newly started TAF/FTC?

[P]: Have not had any of those.

[I]: Tell us about any patients who have switched from TDF/FTC to TAF/FTC and then switched back.

[P]: Don’t have any.

[I]: How, if at all, would the availability of generic TDF/FTC (but not TAF/FTC) influence your prescribing?

[P]: [Inaudible] is a big thing and I think if I would definitely put someone on TDF on the short term while getting the prior authorization or waiting for TAF to become more available in generic form, I think that would probably change my prescribing a lot if it was not otherwise accessible.

[I]: Are there any other experiences or thoughts that you have about TAF/FTC containing regimens that you would like to discuss?

[P]: Nope.

[I]: Thank you for your time. This concludes our interview.
