# Supplementary material for: Health care providers’ decision-making and early adoption of tenofovir alafenamide for HIV preexposure prophylaxis: An inductive qualitative study
Source: PLoS One. 2024 Dec 5;19(12):e0311591. doi: 10.1371/journal.pone.0311591 (PMC11620414; doi:10.1371/journal.pone.0311591)
Supplement: S1 File — (ZIP) [file pone.0311591.s001.zip › Clean transcripts/DedooseDoc_Participant 5 Transcript.docx]

[INTERVIEWER]: I am going to ask you a few questions to learn what you have heard or know about using tenofovir disoproxil fumarate with emtricitabine (TDF/FTC) vs. tenofovir alafenamide fumarate with emtricitabine (TAF/FTC) for PrEP.

Have you heard about using TAF/FTC vs. TDF/FTC for PrEP before today?

[PARTICIPANT]: I have

[I]: What have you heard about the two different regimens?

[P]: Let’s see. TAF is kind of newer, or I guess is used less frequently than TDF. And I think for the most part, I’ve been using TDF more with my patients.

[I]: What are some of the sources of your information about using TAF/FTC vs. TDF/FTC for PrEP?

[P]: I’d say probably more the practice, like what they’ve been using to begin with, what they’re more familiar with and if the patient has any baseline medical conditions that would lean more one towards the other.

[ I]: Have you heard about these two different regimens from colleagues or from pharmaceutical reps or from advertising?

[P]: From colleagues.

[I]: Would that be from preceptors or other residents?

[P]: Preceptors, yeah.

[I]: What about journal articles or CME or any sort of online education?

[P]: Like “Up to Date”, yeah.

[I]: Have you received any guidance or feedback from medical staff at your institution regarding the use of TAF/FTC vs. TDF/FTC for PrEP?

[P]: Not really.

[I]: Walk us through your thought process on how you make decisions regarding prescribing one or the other of these 2 PrEP options.

[P]: I guess my biggest thing I think since I would be more comfortable I would prescribe TDF probably initially unless the patient had some baseline renal dysfunction or was like an older patient where I was concerned more about osteoporosis or bone loss, reasons why you might not want to give TDF and give TAF. But I think personally most of my patients are in a younger population where that isn’t the case, so I don’t think I’ve personally every prescribed TAF before.

[I]: What specific factors make you recommend TAF/FTC over TDF/FTC? How about TDF/FTC over TAF/FTC?

[P]: I guess just like the familiarity of um being more familiar with TDF so I would feel more comfortable for prescribing it for that case.

[I]: Do patient preferences come into play?

[P]: Um, I think I’ve only discussed it once like a patient once brought it up like the patient wondered if there was an efficacious difference between the two so we kind of just talked about it. But then they kind of deferred to me on what I wanted to continue with.

[I]: And did you end up going with TDF?

[P]: Yeah.

[I]: Have you run into any insurance or cost considerations?

[P]: Um, no. I think um for patients who don’t have insurance I’ve always reached out to our case manager who helps with assistance programs.

[I]: What are some reasons/patient characteristics that would influence you to avoid a TAF-containing regimen?

[P]: Nothing I can think off the top of my head, no.

[I]: And reasons/patient characteristics that would influence you to avoid a TDF-containing regimen?

[P]: My biggest things are if they have any baseline renal dysfunction or any severe osteoporosis.

[I]: What experiences have you had with using TAF/FTC for PrEP?

[P]: I haven’t prescribed it at all.

[I]: Do you have any patients on your panel on TAF/FTC for PrEP? But I’m assuming your answer is no?

[P]: Yeah.

[I]: Tell us about any patient inquiries or requests for TAF/FTC PrEP, if any.

[P]: I’ve just had one patient bring it up, just like they heard there was this new drug on the market and is it more efficacious than the other one and would there be a reason to switch – they were already on TDF at that point. And so we had discussed that they both seemed to have similar efficacy and since he was already doing well on TDF we just continued that.

[I]: What are reasons you have not or wouldn’t start a patient on TAF? What are potential benefits and potential risks that you weigh when deciding to prescribe TAF vs. TDF containing regimens? I’ve found that people generally don’t have additional things to add at this point but if you have any other things to add there…

[P]: I’m sure they have their own medication interactions that I just don’t know off the top of my head that might influence you one way or the other if they’re [the patient] on other meds.

[I]: For patients who wish to be newly started on PrEP, do you tend to prescribe mostly TAF/FTC or TAF/FTC? Why?

[P]: Probably TDF.

[I]: And why?

[P]: I think it would just be a similar thing where I think that has just been used more at our practice so that’s the one that I’m a lot more comfortable with.

[I]: For patients on PrEP, to what extent, if at all, are you switching patients to TAF from TDF containing regimens? Why?

[P]: I’ve never switched anyone before, and I think [the switch] would just be if there was just anything in their monitoring bloodwork that was concerning.

[I]: What are some questions/concerns that your patients have raised regarding TAF/FTC? How about TDF/FTC?

[P]: I honestly don’t know if they are even aware that there are differences. I think most of my patients are just like “Oh there’s PrEP” and they just want to start PrEP, but they don’t even know that there are two different options within that.

[I]: Have there been any questions or concerns about effectiveness or side effects?

[P]: Definitely effectiveness, like if they’re both equally efficacious.

[I]: Okay. And then what about any questions or concerns about insurance coverage or cost?

[P]: It has definitely come up, like whether they would have any problems with paying for it. But I think I’ve only had one patient who doesn’t have insurance and before they even started, we made sure they were plugged in.

[I]: Has anyone had issues with pill size?

[P]: Not that anyone has mentioned to me.

[I]: For patients who have been switched from TDF/FTC to TAF/FTC, how has their experience been? How about those who newly started TAF/FTC? But we’ll just skip that one [since provider doesn’t have any patients currently on TAF].

[I]: Tell us about any patients who have switched from TDF/FTC to TAF/FTC and then switched back – but I’m assuming that doesn’t apply?

[I]: How, if at all, would the availability of generic TDF/FTC (but not TAF/FTC) influence your prescribing?

[P]: I think it might make it more I guess like affordable for patients who did have any financial barrier. I don’t think it would change my practice at all.

[I]: Are there any other experiences or thoughts that you have about TAF/FTC containing regimens that you would like to discuss?

[P]: I think it’s just like an area I personally just haven’t thought that much about, like why not just start someone on TAF…I think I’ve just been going off what my comfort level is.

[I]: I’m going to go a little off-script here, but we also want to know whether COVID-19 has affected your prescribing practices at all. Like have you noticed any differences in your prescribing practices since the pandemic started.

[P]: Um, so I haven’t been asked about like bloodwork every three months. Like right in COVID I understand no one wants to leave their house and go to the lab because that also puts them at a higher risk of getting COVID, so I was prescribing it without doing the 90-day post follow-up.

[I]: What interval were you using if any?

[P]: I make sure at least at 3 months either the nurse checked in or I checked in to make sure they weren’t having any concerning side effects or new concerning issues. And then at six months, around June-ish people started coming in and getting lab work done.

[I]: Have you had any experiences with the COVID pandemic affecting patients? Like patients stop taking their PrEP or…

[P]: I had one patient who had stopped taking it or had lost their job and hadn’t realized that we would be able to help with financial assistance. I think he had gone to the pharmacy and they had told him it was hundreds of dollars and he was like “Oh that’s too much for me to afford” and he didn’t realize that we would have been able to help him so he just stopped taking it until his next follow-up. And so, at that point we made sure we got him financially settled and applied for MassHealth and then restarted him back on.

[I]: Have your patients told you about any differences in their risk factor habits like sexual risk factors or injection drug use?

[P]: definitely decreased, like most of my patients who were previously involved with multiple partners are [now] only involved with one partner or no one, just given current COVID times. And I don’t think any of my patients are injection drug users who are on PrEP.

[I]: Great. Any other thoughts about how the COVID pandemic has affected PrEP?

[P]: Overall, I feel like the risk of getting any STD or having multiple partners has definitely gone down. And I know a lot of patients have kind of expressed some distressed over the fact that they used to be able to meet people in very public settings and now given the restrictions bars aren’t open, night clubs aren’t open and so that’s been challenging for them.

[I]: Have you had any patients stop taking their PrEP or start taking it less frequently because they’re having less sexual encounters?

[P]: No.

[I]: That’s pretty much all the study questions.
