# Supplementary material for: Health care providers’ decision-making and early adoption of tenofovir alafenamide for HIV preexposure prophylaxis: An inductive qualitative study
Source: PLoS One. 2024 Dec 5;19(12):e0311591. doi: 10.1371/journal.pone.0311591 (PMC11620414; doi:10.1371/journal.pone.0311591)
Supplement: S1 File — (ZIP) [file pone.0311591.s001.zip › Clean transcripts/DedooseDoc_Participant 6 Transcript.docx]

I: I am going to ask you a few questions to learn what you have heard or know about using tenofovir disoproxil fumarate with emtricitabine (TDF/FTC) vs. tenofovir alafenamide fumarate with emtricitabine (TAF/FTC) for PrEP. Have you heard about using TAF/FTC vs. TDF/FTC for PrEP before today?

S: Yes, yeah I have, yeah.

I: Okay. What have you heard about TAF vs TDF?

S: Um, I believe TAF is like, I don’t know if it’s the newer formulation, but it’s used in Descovy as opposed to Truvada. Unless I have that backwards. But I’m pretty sure I have that right. So I know tenofovir, or sorry, Truvada is sort of what we, the vast majority of what I prescribe, just because it’s been on the market for a lot longer, and then Descovy is the newer option. It came on the market I think kind of late last year, and part of the appeal was it has, sort of less of the effects on your kidneys, supposedly, than Truvada does.

I: Okay. So what are some of your sources of information about TAF vs TDF for PrEP?

S: Um, a lot of it is from the attendings that I work with. I’m over at, is it okay if I say the site where I work?

I: Yeah, totally.

S: I work, my primary care site is at Fenway, and we have kind of sessions on how to prescribe PrEP and what are the best options for PrEP, and part of that is like looking at some of the studies. I know we spent a lot of time looking at the iPrex study, which was for Truvada. I don’t actually know the study for Descovy, but I know, like that’s sort of where the information comes from.

I: Okay. And then, have you received any guidance or feedback from medical staff at your institution regarding the use of TAF/FTC vs TDF/FTC?

S: Uh, like the residency itself or over at Fenway?

I: Um, it just says medical staff at your institution, I think what they’re getting at is what you had just said already, that you’ve had like teaching sessions on it.

S: Yeah, for sure. At Fenway, we definitely have, although really not much on Descovy. Mostly on Truvada. Um, but yeah. As far as at BI, we haven’t had any like residency sessions on it or anything like that.

I: Okay. So then walk us through your thought process on how you make decisions regarding prescribing one or the other of these two PrEP options.

S: For, in general my first choice is Truvada, and I think that’s basically based on just the fact that that’s like what’s used most at Fenway. L ike, I know we, there are certain, kind of prohibitive things, like if somebody has known osteopenia, or like some of bone disease, I wouldn’t use, I guess I’d have a hard time using either of them really, but like especially if they had kidney disease, like notable kidney disease, in that situation I would consider using Descovy first. But in general, and I think this is true of the patients I talk to as well – they want to be on the thing that has been on the market longer, and we have more experience prescribing, and we have sort of like more data to see what it does over a longer period of time. So, you know I give the option to my patients but I definitely always say like “my first choice is Truvada, it’s what I’ve prescribed most, it’s what I’ve seen most”. And then very rarely do I prescribe Descovy first, and then in a few cases I’ve had patients who got like really nauseous when they were taking Truvada, or like intolerable GI side effects. And in those situations we just switched over to Descovy and luckily that went okay.

I: Okay. Um, are there any insurance considerations or cost considerations that you’ve run into?

S: Um, not really. I think F enway does a really good job, if there are any insurance considerations, they have really good financial support, like REALLY good. And I can just flag in our system - they have like a flag button – and I’ll just say “hey, I’m concerned about this patient having access to their PrEP” and then like, it just goes away. It just gets taken out of my hands. It’s kind of amazing. I've never had a problem.

I: Great. Um, alright. So then uh, you’ve sort of already kind of answered this, but what are some reasons or patient characteristics that would influence you to avoid a TAF containing regimen?

S: Um, so I must have had it backwards up front...

I: Well, there’s also the same question for TDF, so we’re asking about both. What would make you avoid TAF, and then what would make you avoid TDF. If anything.

S: If I’m remembering it right... I might have it backwards in my head. Is TAF the newer one? Am I allowed to ask you that? Yeah? Okay. I know the biggest concerns with TAF. So first of all, it hasn’t been on the market as long, so people kind of are hesitant to see what it does. I think it causes dyslipidemia, or there’s some concern it about it having effects, like cardiovascular effects. So like if I had a patient, honestly most of my patients are really young and pretty healthy, so it doesn’t come up that much, um, but if I had a patient who was a lot older, had like really uncontrolled hyperlipidemia, or like really strong history of CAD or something like that I might reconsider, but I have so few patients that fit that bill, honestly I’m older than most of my patients.

I: Fair. Um, alright and then there’s the same question for TDF. Any reasons or patient characteristics that would cause you to avoid a TDF-containing regimen?

S: Um I mean I guess for both of them I could have said insurance, I don’t know if that was a problem, it hasn’t been a problem, but if that was a problem that would be part of it. Otherwise, like kidney disease would be the big thing. Or like they have tried it in the past and got really sick when they were taking it. Like nauseous, or like GI symptoms.

I: Okay. And so then what experiences have you had using TAF/FTC for PrEP?

S: Um, not much. I think I have 2 patients who are on it. Like, it basically like, again I had one patient who just asked if they could take it, and it makes no difference to me, if that’s what they really want, that’s fine. I think he had maybe seen a commercial or something like that. And another one where a patient just had like intolerable, like they had diarrhea and they were vomiting a bunch when they were on Truvada. So we switched them over to Descovy and he did great. That was it.

I: Great. Um, and then next question that we have is do you have any patients on your panel on TAF/FTC for PrEP, which I sort of assume you just answered.

S: Just two.

I: Yeah. Okay, so then what factors, the next question is what factors influenced your decision to prescribe a TAF-containing regimen, okay, that again you sort of just answered unless you have anything else to add... um and then, have you started any patients, like newly starting PrEP on TAF/FTC?

S: Just that one patient. I think, again I think it was like a commercial they saw, and they just asked me up front about Descovy, and I say “I mean, honestly it’s not what I prescribe first, but like if you prefer to take that that’s, you know, by all means.”

I: Okay. And then, for patients who wish to be newly started on PrEP, do you tend to prescribe mostly TAF/FTC or TDF/FTC and why?

S: Mostly TDF, but just because, again it’s been on the market longer. I do, I think like, even quite recently I changed my practice to state that there are multiple options, and by recently I mean like within the past month or two. Um, but again, as soon as I tell somebody that one has been on the market longer, they, people just immediately want to take that. I would do the same thing if I was taking it.

I: Yeah. And then, for patients on PrEP, to what extent, if at all, are you switching patients to TAF from TDF-containing regimens?

S: Uh, from TAF to TDF or the opposite?

I: To TAF, from TDF.

S: Again, just if they’re not tolerating it. That’s basically it.

I: Okay, then what are some questions or concerns that your patients have raised regarding TAF/FTC?

S: Um, mostly it’s regarding like, “do we know what happens long-term on the drug?” and usually I have to say “No”. I mean, I personally don’t, maybe there is a lot of long-term data, but I haven’t seen many people on it for very long, and a lot of that also comes down to the providers I work with. You know, I see the patient, then I go back and talk to my attending then together we go talk to the patient again. And usually the conversation is like, you know these are people who prescribe Truva... you know I’ve been prescribing it for a year plus now, but that’s not very long. They’ve been prescribing it for like, you know, the better part of a decade, or you know, I don’t know, however long it’s been, and they’ve seen people tolerate Truvada really well, for the most part, and have like a lot of success, and um, so for that purpose, I think the vast majority of my patients end up on Truvada.

I: Okay, then um, and then what are some questions or concerns that your patients have raised regarding TDF/FTC?

S: Ba sically like the, the kidney question, like does it cause kidney injury, um, I’ve had a lot of patients ask if they can get their kidney function checked more frequently. Which is not something you see very often, but like, you know, they don’t want kidney disease, which is quite reasonable.

I: Have you had any patients with any concerns about effectiveness, side effects, costs, insurance coverage or pill size?

S: Um, I haven’t heard about pill size, but yes to everything else. Again, the insurance thing just gets taken care of because they have just amazing, um, financial services kind of system in place at Fenway. B ut absolutely, effectiveness is a big question that I get all the time. And I’m happy to say it’s extremely effective if taken appropriately. But, that’s probably, that and side effects are the most commonly asked questions, yeah.

I: What side effects do people ask about?

S: I mean, usually, we just start up front by I mention all the side effects, but the things people are most concerned about are again like, if it’s TAF, they want to know about like effects on heart health and long term effects of it, and if it’s TDF they’re more concerned about kidney function. And then people are always concerned about GI side effects too. I’ve had a lot of patients who have had those GI side effects and they just sort of like go away, so I usually let them know that as well. But um

I: And that’s on TDF?

S: That must have been TDF, yeah. Maybe not a lot, but I’ve had some.

I: Any GI side effects with TAF in your experience?

S: No, but again it’s like a pretty small sample size.

I: Fair. Um, okay so for patients who have been switched from TDF to TAF, how has their experience been?

S: Um, great. I’ve only had one patient who switched. I have one who was on it first, and one who switched. The guy who switched did great. He like, all of the, he had like really bad GI side effects, and then they like essentially... he like tolerated Descovy really well.

I: And then the same question but for those who were newly started on TAF/FTC - how was their experience?

S: He did fine. He... no... no side effects, yeah.

I: Okay. Has anyone complained of any adverse events or negative effects from TAF/FTC?

S: Um, not that I am aware of, nope.

I: Okay. And then, tell us about any patients who switched from TDF to TAF, and then switched back, if any.

S: I have not seen that, so I don’t know.

I: Okay. Um, how if at all would the availability of generic TDF/FTC but not TAF/FTC influence your prescribing?

S: I think it would, I think it would um, it’s kind of hard to (inaudible). So like, if I didn’t know that we had these amazing financial services, I would, it would influence it a ton. I mean, it would just influence it just in the fact that I would mention that to everybody who walked in the door right, because cost is certainly prohibitive for a lot of people. And even with financial services, I’m sure there are plenty of patients who have trouble getting access to it, I have a lot of patients who have a really hard time kind of, getting in for follow-up, and those are the kinds of patients who financial services are also challenging for, because that requires a fair amount of follow-up, or at least contact. Um, so for a patient like that I would let them know up front, like this is going to be a lot cheaper, right, but um. Yeah, I think it would definitely have an impact.

I: Okay, and then are there any other experiences or thoughts you have about TAF/FTC containing regimens that you would like to discuss?

S: Um, not really, it’s really nice that there’s a second option on the market I would say. Just the fact that I was able to take this patient who couldn’t tolerate one medication and keep him on PrEP was to me very valuable, so, yeah.

I: Great. And then, this is, that’s sort of the end of the official script, but we’re working on adding some additional questions, just if COVID has affected your PrEP prescribing practices. So as a prescriber, have you noticed any effect from the COVID pandemic?

S: Uh, absolutely, yeah a ton. I’ve gotten a lot of patients who were really hesitant to go into lab. They were really worried about getting COVID while they were at the lab. Kind of a surprising amount of patients who just really did not want to have any human contact. Which actually is a good thing, overall, right, it’s like they’re actually quarantining, but at the same time, like, you know, they’re new on PrEP, we need to like have semi-regular, at least every 3 month lab checks up front. Can space that out later, but like, you know, it’s not safe to put somebody on PrEP and then like not ever check their kidney function, right. I’ve definitely had a couple patients who were completely lost to follow-up in the COVID process, which is really frustrating and upsetting. Yeah, I feel bad about it.

I: Yeah. Have you changed your prescribing, like approach at all?

S: I think it’s hard to say, because I’m kind of beholden again to the person I worked with that day, and this is like not, I'm not like saying anything bad about them, but like a lot of the providers at Fenway because they’re so competent, and we just did like, I didn’t even know what PrEP was before, I mean I knew it existed, but I had never prescribed, never even thought about it. It is like legitamately probably a third of what I do at Fenway. So like, these are people who just have so much experience providing PrEP, and like, a lot of them are really strict. They say like, “if you cannot come in for lab draws, like we can’t prescribe you PrEP”. And I understand that right, like there were patients in the iPrex trial who like didn’t get HIV testing, were put on PrEP then developed resistance. And they’re like really concerned about breeding resistance and about causing kidney injury – I'm sure they’ve seen kidney injury in people who have been on PrEP. So for those, reasons, I haven’t changed my practice, you know, sometimes I feel, like you know I do want to be more lenient and say like “I know it’s really hard to get to lab, so could we push it off” but I’m kind of beholden to the people I’m working with.

I: Mmhmm. And then, have you noticed any change in terms of, like, patient uh, I guess like activity or um, kind of like, the patient experience with PrEP during the COVID, um...

S: Yeah, for sure. Are you talking about sexual activity, or just kind of...

I: Well, like both patients, have you had patients who have changed their PrEP practices or their risk factor practices?

S: I think risk factor practices definitely. The patients that I was able to check in with during the COVID thing, which actually is probably a fair amount of them, were just having a lot less sex, right? It’s really hard to have sex if you’re not like, seeing other humans. So, like a lot of them had said “you know, do I need to be on PrEP?” and for the most part I said like, “if you plan on returning to, like, having multiple sexual partners, having unprotected sex, things like that, probably a good idea just to stay on PrEP, because I don’t know when you’re going to start, sort of like, whatever your practice will be for becoming more lenient with quarantining”. Anecdotally, like I probably saw my first few patients a couple weeks ago who have kind of started to ease off the quarantine, even though they probably shouldn’t be, and now are kind of like, risk factors are increasing again, so to that extent I feel good that I kept them on PrEP. But I definitely had patients come in and ask like “Hey, if I’m just like not having sex, should I be on PrEP”, um, and I, you know, if they, I don’t think there’s anybody I took off PrEP, but I said like “if you actually think this is going to be your practice going forwards, of course you shouldn’t be on PrEP” but... but I actually don’t, I don’t think I ended up taking anybody off of PrEP. With regards to their risk factors. As far as like, taking the medicine itself, um, not that I’m aware of, other than the few patients that I’ve lost to follow-up, which was again really frustrating.

I: Okay great. That’s pretty much all I had.
