# Supplementary material for: Health care providers’ decision-making and early adoption of tenofovir alafenamide for HIV preexposure prophylaxis: An inductive qualitative study
Source: PLoS One. 2024 Dec 5;19(12):e0311591. doi: 10.1371/journal.pone.0311591 (PMC11620414; doi:10.1371/journal.pone.0311591)
Supplement: S1 File — (ZIP) [file pone.0311591.s001.zip › Clean transcripts/DedooseDoc_Participant 7 Transcript.docx]

I: I am going to ask you a few questions to learn what you have heard or know about using tenofovir disoproxil fumarate with emtricitabine (TDF/FTC) vs. tenofovir alafenamide fumarate with emtricitabine (TAF/FTC) for PrEP. Have you heard about using TAF/FTC vs. TDF/FTC for PrEP before today?

S: Yes. I have.

I: And what have you heard?

S: So I’ve heard that TAF has less risk of side effects compared to TDF, specifically I believe the risk of kidney dysfunction, like renal tubular acidosis is less, as well as osteoporosis risk. And I think it was just recently approved through the FDA and the other thing I think I know about it is that it’s been approved mostly for men who have sex with men and it has not yet been studied in women.

I: And what are some of the sources of your information about using TAF/FTC vs TDF/FTC for PrEP?

S: You know, honestly, I think it’s the FDA approval and just sort of my knowledge from like, practicing with my attendings who have been advising me as patients are asking questions about this.

I: Um, have you gotten any information from uh, pharmaceutical reps, advertising, journal articles, continuing medical education, or online information?

S: Mmm, I don’t think so, no.

I: Okay. Um, have you received any guidance or feedback from medical staff at your institution regarding the use of TAF/FTC vs TDF/FTC for PrEP?

S: Yes. Yup.

I: Okay, so walk us through your thought process on how you make decisions regarding prescribing one or the other of these two PrEP options.

S: So right now, um if patients are otherwise... so if patients are not on any PrEP and they’re otherwise young and healthy, I generally still lean towards TDF/FTC. Um, just because I know insurance is going to cover it, and it has more data, and I think that the risk of osteoporosis if they’re young and healthy is low, and I think that the, you know the risk of the kidney dysfunction is low because we monitor it very frequently. Um, so in a... yeah. That’s kind of my first thought. And then if patients come to me already on PrEP, and they’re asking to be switched to TAF, um, instead of TDF, um, generally again I use the same thought process where unless they have any extenuating risk factors, like a history of kidney disease, or they’re, you know, older in age, other co-morbidities, I generally advise them to stay with TDF/FTC. Um, however, if they’re really interested in trying it, than I have, I think I’ve tried 2 patients on TAF/FTC. Um. And it also, the other thing I think is insurance coverage – we're not quite sure – at least that’s how it was a few months ago.

I: What specific factors make you recommend TAF/FTC over TDF/FTC?

S: Yeah, so like I mentioned I think, um, kidney dysfunction, um, advancing age, um, you know, things that would predispose you to osteoporosis, like maybe they have, um, some other like, rheumatologic condition that they’re on prednisone for. Or... or they otherwise have like a history of fractures. So it... those two things probably mostly would be what I would, um, lean towards that for.

I: Okay. And what about TDF/FTC over TAF/FTC?

S: Um, you know I think the major thing, like I said, is insurance coverage, because I think they’re both have been proven efficacious, in um, men, and I (inaudible) the other major thing for TDF would be women. If women are started on PrEP then definitely I would lean towards TDF/FTC.

I: Okay. Do patient preferences come into play?

S: Um, sometimes. I mean, generally like I said, I advise most patients to stay on TDF if they’re already on that, um, medication, but if they’re really really interested in trying TAF and they’re really concerned, I think it’s reasonable to try that in men who have sex with men.

I: Um, and then, any patient characteristics like gender, medical conditions... you sort of already answered that, but if you have other things to add.

S: Yeah, I think I, I don’t have anything else to add.

I: Okay great. Um, what about insurance or cost considerations?

S: Yeah definitely. I think my impression was that um, TDF/FTC is you know, universally covered, and the pharmaceutical manufacturer has a, a copay assistance program as well, so if any patients have any concerns about costs I’ll lean towards TDF/FTC. And it also makes you want to keep patients on it who are already on it, and try that one first before TAF Because I think insurance coverage has been variable since it’s so new, newly approved.

I: And what are some reasons or patient characteristics that would influence you to avoid a TAF-containing regimen?

S: Women. Um, if women are interested in PrEP, that would be the main reason I wouldn’t want to do TAF, until we have more data.

I: Yeah. And then any reasons or patient characteristics that would influence you to avoid TDF-containing regimen?

S: I think I already answered that, kidney dysfunction, osteoporosis.

I: Great. Um, and then what experiences have you had using TAF/FTC for PrEP?

S: Um, I have used it only a few times, and you know, it was fairly painless, insurance did actually cover with a little bit of a higher copay, but patients seemed to like it, and I haven’t deviated at all, I’m still checking, you know, renal, you know kidney panels every three to six months, and no real change in my practice, but... but I have used it a few times

I: Okay. Well, the next question is do you have any patients on your panel on TAF/FTC for PrEP?

S: Yeah, I think 2 or 3.

I: Okay.

S: But the majority are TDF still.

I: Okay. And what factors influenced your decision to prescribe your patients a TAF-containing regimen for PrEP?

S: Yeah I think the number one factor is patient specific factors, like patients who are interested in wanting to try that regimen. Or again, like I’d already answered, if they have like, kidney problems, or osteoporosis.

I: Um, and sort of a similar – please describe your decision-making with selecting TAF/FTC for any patients that were newly started on PrEP? Versus those who were switched from TDF/FTC.

S: Yeah I... Have I already answered that? I think basically if they’re interested in TAF, and if they’re like an advanced age, or kidney problems, I would, I would consider startiung them on TAF.

I: Alright, um. So then, for patients who wish to be newly started on PrEP, do you tend to prescribe TAF/FTC or TDF/FTC and why?

S: Um, TDF because of insurance coverage.

I: Okay. For patients on PrEP, to what extent, if at all, are you switching patients to TAF from TDF-containing regimens and why.

S: Not very often. And kind of the reasons I already outlined as to why.

I: Yeah. Okay. And what are some questions or concerns that your patients have raised regarding TAF/FTC?

S: Um, you know they... their major questions are just they’ve heard that it’s, you know, safer. That’s kind of the big advertiser at this point, so they’re always interested if, you know, they always want to be on a safer option, so they ask a lot about that.

I: Um, any questions or concerns that patients have raised regarding TDF/FTC?

S: Um, I suppose in the opposite vein, you know, they’re wondering why TDF isn’t as safe as TAF?

I: Okay, any concerns or questions about effectiveness, side effects, insurance coverage, costs or pill size?

S: I would say yes – side effects and insurance coverage.

I: Okay. Tell me more about the side effect questions that you’ve gotten.

S: I think mostly, um, they don’t ask specifically in detail, most of them are just asking in general “Oh I’ve heard TAF is safer than TDF and can you explain a little bit why?”. And I think a few have asked about kidney function specifically. I haven’t heard any questions of osteoporosis that I remember.

I: Okay. And then for patients who have been switched from TDF/FTC to TAF/FTC, how has their experience been?

S: I think their experience has been good. Um, you know like I said insurance coverage worked, it was a little bit more expensive but they were happy with the switch and they haven’t really noticed any side effects... yet.

I: Okay. And then how about those who were newly started on TAF/FTC?

S: Uh, honestly I don’t think I have any new starts. I think all of mine were switches.

I: Um, any adverse events or negative effects that you’ve noticed?

S: No.

I: Okay. And then tell us about any patients, if applicable, who have switched from TDF/FTC to TAF/FTC and then switched back to TDF/FTC?

S: I haven’t had any of those patients.

I: How, if at all, would the availability of generic TDF/FTC but not TAF/FTC influence your prescribing?

S: Well, I would, as.... it would probably influence my prescribing a lot if it’s significantly cheaper and it causes the insurance coverage concerns with TAF, um. So. I would guess I’d lean more towards TDF.

I: Yeah, okay. And then, are there any other experiences or thoughts that you’ve had about TAF/FTC containing regimens that you would like to discuss?

S: I don’t think so.

I: Okay. Alright. And then, that was kind of the end of like, our TAF/TDF questions, but we also have been asking people just a couple of questions about how the COVID pandemic has affected their PrEP prescribing. Um, so as a prescriber, has the COVID pandemic affected your PrEP prescriptions... or your prescribing practices, at all?

S: Um, I would say it has. Um, made me be more lenient in making patients come in for like routine kidney function checks, because it’s a high burden to come in for labs, and there’s limited... there’s always some risk with coming into a healthcare facility to get labs. So I’ve sort of been generally just refilling these medications as long as their kidney function was stable at their last check, I’ve let them go, you know, six months to a year, as long as they’ve been stable previously.

I: Um, and then have you noticed any effects, um, in terms of your patients and their PrEP, like hab... like taking habits?

S: Yeah, I would say number one I’ve noticed less new patients starting on PrEP. I... I don’t think I’ve actually started a new patient on PrEP since the pandemic has started, so most of them have just been renewals. And I think, I've also had a few patients who have stopped PrEP, because they’re... you know... not as sexually active.

I: Approximately what proportion of your patients, um, have either stopped or had some sort of effect?

S: I would say, like 5-10% maybe, not that many. 5%.

I: Okay. Alright. Any other thoughts on how the COVID pandemic is affecting PrEP?

S: Those are the major ones.

I: Great. Awesome.
