# Supplementary material for: Health care providers’ decision-making and early adoption of tenofovir alafenamide for HIV preexposure prophylaxis: An inductive qualitative study
Source: PLoS One. 2024 Dec 5;19(12):e0311591. doi: 10.1371/journal.pone.0311591 (PMC11620414; doi:10.1371/journal.pone.0311591)
Supplement: S1 File — (ZIP) [file pone.0311591.s001.zip › Clean transcripts/DedooseDoc_Participant 8 Transcript.docx]

I: I am going to ask you a few questions to learn what you have heard or know about using tenofovir disoproxil fumarate with emtricitabine (hereafter referred to as TDF/FTC) vs. tenofovir alafenamide fumarate with emtricitabine (hereafter referred to as TAF/FTC) for PrEP. Have you heard about using TAF/FTC vs. TDF/FTC for PrEP before today?

S: Yes.

I: Okay. And what have you heard about TAF/FTC vs TDF/FTC?

S: Uh, not much, um. Obviously you know, using my knowledge from treatment for HIV, you know the TAF component is more friendly to the kidneys, so, I guess, you know, you probably want to preferentially use that, especially if you have some concerns about kidney function.

I: And what are some sources of your information about TAF/FTC vs TDF/FTC for PrEP? Some possible options: colleagues, patients, pharmaceutical reps, advertising, journal articles, online information, others...

S: Uh, I would say online information and colleagues.

I: What sorts of online information?

S: Sorry, why?

I: What sorts of online information?

S: Uh, I know, you know, I guess the HIV guidelines or, I don’t know if they have that, or the CDC – I know they have something on PrEP.

I: Um, and then have you received any guidance or feedback from medical staff at your institution regarding the use of TAF/FTC vs TDF/FTC for PrEP?

S: No.

I: Then walk us through your thought process on how you would make decision regarding prescribing one or the other of these two PrEP options?

S: Oh, um, hmmm...

I: So sort of like specific prompts would be like what specific factors would make you recommend TAF/FTC over TDF/FTC or TDF/FTC over TAF/FTC?

S: Again, I think the one thing that um, comes to mind is the renal function. I think that would be the only difference. I’m actually not familiar if they have... I would presume they’re same frequency and dosing... I mean, you know, um, so I think the only thing would be maybe the renal function?

I: Great, and then, do patient preferences come into play? Insurance considerations? Cost considerations?

S: Yeah, absolutely, yeah. I think yeah. Patient preference, again it’s important. Insurance is very important, but also of course if it’s safe for the patient.

I: Um, so then what are some reasons... and I will say some of these questions are repetitive, so sometimes they’re kind of asking the same thing... so what are some reasons, patient characteristics, that would influence you to avoid a TAF-containing regimen?

S: TAF-containing? Um. I mean, I think if it... if it’s a matter of cost, and the patient cannot afford it and won’t take it, then yeah.

I: And then what reasons or patient characteristics would influence you to avoid a TDF-containing regimen?

S: Um, I mean, if there is contraindication to the medicine, you know. But similar... I mean, I presume, I heard that Truvada became generic now, so maybe that’s going to be less of a... the cost issue might be less of an issue... but yeah.

I: Cost less of an issue for Truvada now that it’s generic you mean?

S: Truvada... yeah. But obviously, I think as with any medications, especially in our field, you know, cost is important. And insurance.

I: Great. Um, yeah. It’s actually – a later question that I will just skip to now since it’s relevant is “how does the availability of generic TDF/FTC but not TAF/FTC influence your prescribing?”

S: Uh, I think it’s going to be discussion between myself and the patient. But if cost is an issue, and I believe that the cost might stop the patient from getting... you know taking the medication, then I would prefer the TDF, yeah. The generic TDF.

I: Makes sense. So then what experiences have you had using TAF/FTC for PrEP, if any?

S: Um, zero.

I: Okay. Do you have any patients on your panel on TAF/FTC for PrEP? This may be...

S: Uh.. No.

I: Um, so have you had any patient inquiries or requests for TAF/FTC? Um, also known as Descovy, for PrEP?

S: No.

I: If you did have a patient inquiry or request, how would you respond to one? Like specifically for TAF/FTC?

S: Sorry say that again, the question was cut off.

I: Yeah, sorry I also said it in a weird order. If you did have a patient inquire, or request, about TAF/FTC for PrEP, what would your response be?

S: Um, well I guess if... I mean, obviously I may need to inquire more about why they want to take it, etc. You know, what’s their risk factors. You mean specifically for TAF or in general about PrEP?

I: Yeah, like specifically for TAF.

S: Oh. How would I respond? Um.

I: I guess like is there anything different that you would tell someone who asked for TAF/FTC vs who asked for TDF/FTC?

S: I don’t necessarily.... no. I don’t think so. I would approach it the same way. Again, in my mind TAF and TDF are equivalent apart from their side effect profile.

I: Mmmhmm. Then, what are reasons you have not, or would not start a patient on TAF? TAF specifically?

S: Um. Clinical? I mean intolerance...

I: It’s just kind of an open-ended question, so you could answer it however, could be potential benefits, potential risks....

S: ...I wouldn’t prescribe it.... If for some, only if for some reason there’s... I can't think of any reason why I wouldn’t. From a clinical perspective, unless in the rare sort of occasion I can tell that there is an issue with intolerance, but I’m not sure how I would know that.

I: Um, so and then what are potential benefits and potential risks that you weigh when deciding whether to choose TAF or TDF in your regimen?

S: Again, safety... I think is first. And then probably cost is second.

I: So then, for patients who wish to be newly started on PrEP, do you tend to prescribe TAF/FTC or TDF/FTC?

S: (laughs) Well, in my very very limited experience, which is almost zero. I think it was Truvada, I mean TAF... TDF/FTC. Yeah. But that’s just because it was the most... the most recognizable and the one that was most available, at least initially.

I: For patients who are already on PrEP, to what extent, if at all, are you switching patients from TDF to TAF containing regimens.

S: Um, I had one patient – I didn’t, I wouldn’t. I mean, um, if they’re tolerating and they’re not having any side effects or any sort of, you know, or the patient is not requesting it, then I wouldn’t necessarily change it.

I: Okay, so then what are... what are some concerns or questions, if any, that your patients have raised regarding TAF/FTC?

S: Um, I haven’t had that experience, to be honest.

I: Um, how about TDF/FTC?

S: Um

I: Any concerns about effectiveness, side effects, insurance coverage, costs, pill size? Any of those things that patients have brought up?

S: I think it was mostly about side effects

I: Were there any specific side effects the patients were concerned about, or just kind of general question?

S: I think general, just general question, there wasn’t anything specific.

I: Um, for uh, so the next question probably not really applicable, so for patients who have been switched from TDF/FTC to TAF/FTC, how has their experience been? I think you said you haven’t had any of those patients.

S: No.

I: Um, and then, the next one is how about those who were newly started on TAF/FTC, which it sounds like you haven’t done either.

S: Nope.

I: Um, okay, have you had any patients who had any adverse events or negative effects that you’ve noticed, with any of their PrEP regimens?

S: No.

I: Okay. Um, and then, the last, the next question, probably also not applicable. Tell us about any patients who have switched from TDF/FTC to TAF/FTC, and then switched back. I assume because you didn’t have any switched you didn’t have any switched back.

S: No

I: And then the last question.. Err the last of the kind of like standard questions, is just about the generic TDF vs TAF. And then just, are there any other experiences or thoughts that you have about TAF/FTC and TDF/FTC containing regimens that you would like to discuss.

S: Uh, well not really. I mean, I mean, it’s also. A lot of it stems from ignorance of the whole thing. And lack of experience, rather. So I’m not... yeah.. So...

I: With PrEP itself, not with either of the regimens.

S: With PrEP itself, I’m uh, yeah. I mean, to be honest like my patients panel, I mean, I was thinking about this. Half my patients already have HIV already or half of them don’t fit the sort of, like classic PrEP.

I: Yeah that’s fair... we don’t have... I’ve had a few patients on PrEP but yeah we don’t have... a lot of patients on PrEP in our clinic for that sort of reason – they tend to get managed in HCA or in Fenway. I’m interviewing those providers too, but, figured we’d do ID as well... since... some you know, interesting stuff.

S: Yeah.

I: So that’s sort of like, the uh, kind of like the original part. We also had tacked on some questions, um, just about COVID and PrEP. Um, so as a prescriber, have you noticed any difference, or any changes in your prescribing of PrEP since the COVID pandemic started?

S: Uh, I don’t think the COVID pandemic would change my prescribing of PrEP.

I: Have you had um, any observed effects on patients and patient’s PrEP use from the COVID pandemic?

S: No.

I: Okay. Any... any thoughts about COVID and PrEP?

S: It’s a loaded question, but presumably there is something there.... I don’t... I wasn’t even aware of it.

I: Yeah, no. That’s okay. If there’s... if you haven’t had any experiences with it, that’s totally fine.

S: I wasn’t aware of PrEP, with it...

I: We started doing this project before COVID, then we like tacked on, I mean like, we got it IRB approved, of course, but tacked on those questions just because we figured there might be some interesting answers, but it seems like it’s kind of early days, anyway, for that.

S: yeah.

I: So then the last thing I just have is the demographic form.
