# Supplementary material for: Health care providers’ decision-making and early adoption of tenofovir alafenamide for HIV preexposure prophylaxis: An inductive qualitative study
Source: PLoS One. 2024 Dec 5;19(12):e0311591. doi: 10.1371/journal.pone.0311591 (PMC11620414; doi:10.1371/journal.pone.0311591)
Supplement: S1 File — (ZIP) [file pone.0311591.s001.zip › Clean transcripts/DedooseDoc_Participant 9 Transcript.docx]

Interviewer (I): I’m going to just start by asking you a few questions to learn what you’ve heard and what you know about using tenofovir disoproxil fumarate with emtricitabine (hereafter TDF/FTC) vs tenofovir alafenamide fumarate with emtricitabine (hereafter TAF/FTC) for PrEP. So have you heard about TAF/FTC vs TDF/FTC for PrEP before today?

Subject (S): Yes and I’ve prescribed both, um, as PrEP, so I am quite familiar with having done a lot of work with PrEP before. Um so yeah, I’ve prescribed TDF/FTC, or you know, Truvada, you know quite frequently for PrEP. Um, I also did work at Fenway Health over the last 2 years, during my residency, so I very commonly prescribe it there to a populations who I believe could benefit from it the most. And I also have prescribed TAF/FTC too.

I: All right, and what do you know about the difference between TAF/FTC and TDF/FTC?

S: Yeah, so with TDF/FTC, I know that I think it was originally approved in 2012 for it, so it was the first formulation of PrEP as we know it. And so, that’s something that I very commonly prescribe, mostly for I would say, the populations I’ve prescribed it most for are MSM and trans women. And then, just recently the FDA approved, as I know you know, the TAF/FTC formulation. Essentially in the DISCOVER trial I know it was seen as non-inferior, and also improved renal and bone density safety profiles. So yeah. And I’ve prescribed it in a limited form since then.

I: Great, so what are some of the sources of your information about using TAF/FTC vs. TDF/FTC for PrEP? Some options might be colleagues, patients, pharmaceutical reps, advertising, journal articles, continuing medical education, online information, anything else?

S: Um, colleagues definitely, and also the phase 3 trial data from DISCOVER that was presented at CROI last year, uh, that was also a big source of information too.

I: Great, um, have you received any guidance or feedback from medical staff at your institution regarding the use of TAF/FTC vs. TDF/FTC for PrEP?

S: Yeah, and I can give you one example. So the only time I ever prescribed TAF/FTC was in one instance, it was last year at Fenway, and so I did receive some clinical guidance from a colleague regarding this. If it’s helpful I can give you the clinical context.

I: Sure.

S: It was a young person with type I diabetes, no renal dysfunction, but given the fact that, you know, assuming later on in life would develop some type of renal dysfunction started on TAF/FTC as opposed to TDF/FTC.

I: Hm, makes sense. Um, so then walk us through your thought process on how you would make decisions regarding choosing one or the other of these PrEP options.

S: Yeah. I think for most folks who are already on TDF/FTC, you know that is one common thing that I see, “should I be switched over to TAF/FTC?” And so I’m telling a lot of patients, “No, you don’t necessarily need to be switched over, you know, it’s likely going to come off patent, so we may have a cheaper alternative” - I don’t know exactly what the most up-to-date information of that is, in terms of expected timeline for that, but anyways, so that’s what I most commonly will tell folks. For those that have either renal dysfunction, I know with eGFR between 30 and 60 you can use TAF, so if someone has renal dysfunction and would be an appropriate candidate for it, then I would start TAF/FTC, or if they have some sort of bone mineral density loss then yeah, I would probably use that too, but I haven’t run into that clinically yet, so.

I: Okay. You’ve sort of just answered the next question already, but if you have any other thoughts, um, the question was what specific factors make you recommend TAF/FTC over TDF/FTC?

S: Yeah, and so basically, what I just, you know had said previously, and then also, I think also with the DISCOVER trial it was limited with only using MSM and trans women, so um, yeah, we don’t know essentially yet if it’s generalizable so I wouldn’t use it also for those practicing receptive vaginal sex, just because we don’t know yet some of that data, generalizability.

I: Mmhmm. Great, and that’s basically an answer to the next question, which is what specific factors would make you recommend TDF/FTC over TAF/FTC, so that’s perfect.

S: Yeah, so kind of all of the above.

I: Yeah, these questions definitely ask the same things sometimes, so if you feel like you’ve already answered something, feel free to just say that. Um, so then how do patient preferences come into play?

S: Um, you know that’s a good question, um, I think if some patients of mine, who are just curious about learning more information and asking “Do I need to be switched?” Um, you know I’ll certainly let them know that, you know, it’s perfectly fine, and I think if it’s the right clinical context, if they don’t have any underlying renal disease, bone mineral density dysfunction, it’s perfectly fine in my opinion to keep on the TDF/FTC, but you know, if they’re strongly interested in, and have done a lot of research and have a lot of strong preferences, I’m not opposed to it, but I haven’t run into that clinically, but I would definitely be open to switching, while also counselling them that, you know, this is still only preliminary data, etc, so

I: Great, and then, any, how do patient characteristics like gender, medical conditions come into play?

S: Yeah, so I think, the above, you know, if they’re MSM or trans women practicing anal intercourse, certainly receptive anal intercourse, unprotected, then yeah, they would definitely be good candidates for it, but outside of that we don’t technically know the generalizability of that, so

I: Great. Any insurance considerations or cost considerations?

S: Uh, you know, that’s a great question. So, I don’t know, feel free to let me know at the end of this if you know more up to date information than I do, but I don’t think Truvada has come off of patent yet, um, so, I think once that does happen, if it hasn’t already, then it would be a lot cheaper, so I think that would be the main cost consideration. I think I’m very fortunate to be practicing here, where we have so much support from pharmacy staff and our prior auth team, etc. That if patients were to encounter cost issues with TAF/FTC the certainly we could see if there’s, you know, I don’t know exactly what Gilead is offering in terms of additional co-pay coupon cards for TAF/FTC, I’m assuming it covers it, um. As you know, because I would use that commonly for folks on TDF/FTC if they run into cost issues, I would just refer them to that site, Gilead, the co-pay coupon card, and you know our prescription and prior auth teams are also really helpful with that, so that’s kind of some of the insurance issues, you know, addressing that.

I: Makes sense. Um, and then what are some reasons/patient characteristics that would influence you to avoid a TAF-containing regimen?

S: So, that would be renal dysfunction less than 30, because I know you’re not supposed to use it for that. Um, and then also patient characteristics that I mentioned before not included in the DISCOVER trial in terms of sexual behavioral practices. And then, you know, I think also another population that we also take a look at too, that of course generates a lot of interest, is injection drug use and so, um, I pretty much go based off of also what I think UpToDate recommends. That, you know, that population was not included in the DISCOVER trials, it’s a separate population, so I think I would just take a look at what other risk factors do they have, as well, that would help make that decision for me.

I: Makes sense. What reasons or patient characteristics would influence you to avoid a TDF containing regimen?

S: So to avoid TDF-containing regimens, so if they already have renal dysfunction, then yeah, then I would probably, you know, I would offer them TAF/FTC or some sort of bone mineral density dysfunction. Um, but, yeah. But if they were practicing receptive vaginal intercourse, etc, outside of, you know, they did not have renal dysfunction, then I would promoted TDF/FTC.

I: Great. Excellent. So what experiences have you had using TAF/FTC for PrEP?

S: That’s the only one that I mentioned actually, yeah. Um, and so, the patient panels are just so different, as you know here, so I do primary care here full time now, so um, so I know we have the embedded PrEP clinic here. Um, but yeah, I haven’t honestly come across too many patients of mine yet who would be eligible for it.

I: That’s all right. Um, do you have any - you may have just answered this - do you have any patients on your panel on TAF/FTC for PrEP?

S: No

I: Have you had any patient inquiries or requests for TAF/FTC for PrEP?

S: As a resident at Fenway, yes.

I: Okay, and how did you respond to those?

S: Um, as I mentioned before, you know, if they were just curious about it but they were doing well on TDF/FTC and all their safety labs were all otherwise normal and they were stable on it, then I wouldn’t be in a rush to switch them.

I: Great. Um, so for patients who wish to be newly started on PrEP do you tend to prescribe mostly TAF/FTC or TDF/FTC and why?

S: Hmmm. Uh, that’s a great question. Um, so you know I think in my personal practices I would still want to see more data on TAF/FTC to initially prescribe that up front. Um, so you know I would probably prescribe TDF/FTC unless there was some compelling reason to do TAF/FTC.

I: Okay, so then for patients who are already on PrEP, to what extent, if at all, are you switching patients from TDF to TAF containing regimens? And why?

S: Yeah, so I think we already answered that too, but yeah certainly if they had a compelling reason to switch to TAF/FTC based on their eGFR or bone mineral density, do they also have risk factors for osteoporosis? Have they been on chronic steroids in the past? Things like that. Are they a smoker? Are they frail, you know, then I might be more willing to switch them over to TAF/FTC.

I: Great. Um, so then are there any questions or concerns that your patients have raised regarding TAF/FTC?

S: Um, no, nothing that patients have raised in terms of concerns.

I: What about TDF/FTC, patient concerns or questions?

S: I always get the question “is this safe for my kidneys” you know. So I think patients have a lot of awareness, they’ve heard a lot through either the media, um you know, certainly with the lawsuits against Gilead and just information that they’ve read online. I think that’s the most common thing. And I do find that patients are also aware about the potential side effect with bone mineral density too. Patients are pretty savvy about that.

I: And how do you respond to that?

S: Um, you know I present to them some of the data from the iPrex study, you know I let them know that a very small minority developed renal dysfunction, and that after stopping, Truvada, almost a large majority of them completely had recovery of their renal function, and then also with the loss of bone mineral density I just let them know that there was, in terms of clinical significance, there were no clinically significant pathologic fractures, things like that. So, you know, and then I usually try to reassure them of that, so.

I: Um, has anyone brought, any patients brought up, any questions about effectiveness, side effects, insurance coverage, pill size?

S: No, I haven’t encountered that before.

I: Mmkay.

S: A lot of cost issues though sometimes with Truvada, yeah. Lots of cost issues. And then that’s where, you know I’ll try to help them navigate Gilead co-pay assistance, things like that, so.

I: Um, so then for patients who have been switched from TDF/FTC to TAF, how has their experience been? Sounds like N/A there.

S: Yeah, you know I never actually had follow-up with that other person who I’d switched, who I don’t know if they saw another provider after that

I: Okay. Um, and then, how about those who are newly started on TAF/FTC.

B: And so yeah, I haven’t started anybody newly on it yet.

I: Okay. Um, and then, tell us about any patients, if applicable, who have switched from TDF/FTC and then switched back.

S: So, not applicable.

I: Okay. Um, how, if at all, would the availability of generic TDF/FTC but not TAF/FTC influence your prescribing.

S: Um, yeah so definitely would lend me to prescribe that certainly if there are cost issues.

I: [simultaneously] It would point you towards…

S: And there’s an otherwise.. Towards TDF, the generic

I: Any other experiences or thoughts that you have about TAF/FTC vs TDF/FTC containing regimens that you would like to discuss?

S: No, I mean, think it’s really exciting. I think with more data and longer-term data I definitely do think it, there will be a pendulum swing towards that, and more general acceptability of it. So you will see, you know, and then who knows what’s going to happen with you know longer, you know other formulations of PrEP, you know longer acting injectables, you know, it’s changing so fast, so.

I: It is true, it is quite hard to keep up sometimes. So those were all the questions we had about TAF vs TDF, um we tacked on a couple of questions about COVID, just because we’re here and why not.

S: Yeah

I: So my first, kind of like COVID PrEP question is “as a prescriber, have you noticed any influence of the COVID pandemic on your prescribing practices for PrEP?

S: Oh, uh, that’s a really great question. So, this hasn’t come up for me, just because, or you know what? Actually it did. Yeah. Early on during the pandemic - March, April, May - I was deferring labs. So instead of having patients come in for their three month labs, a small subset of mine who were stable, you know, we waited until six months. Certainly if they had any STIs recently, or anything like that, I would say, “please come back in, let’s do your labs”. But if they were otherwise very low risk, they haven’t had STIs recently within the last year, their renal function has always been normal, then I said to them “you know what, let’s defer your labs until six months from now, so”.

I: Now that the numbers are rising again, how do you anticipate your practices, regarding those, that issue, kind of, continuing to change, or…

S: Yeah, I mean, so like right now, I mean and this is also just outside of PrEP, I’ve been bringing a lot of my patients back in for labs now and for follow-up of other medical conditions that unfortunately have been pushed back, so I think probably within the last two to three months I would say for all those patients I would definitely bring them back in to do labs if we had deferred. But yeah, I mean if we’re going to start to see further shutdowns, things like that, then I think it would be totally appropriate again for that select population to defer labs.

I: mmhmm. All right. And then from a patient perspective, have you had any patients tell you about any influence that the COVID pandemic has had on their PrEP uptake and usage?

S: Um, I would say just a lot of people are, you know a lot of people are less sexually active right now, and so the other thing too that you know I would also do too is that if for patients of mine who I know very well, they’ve been seen very routinely in follow-up, their labs have always been stable, otherwise relatively low risk and if they’re wondering “Should I still be on PrEP during this?” I may also say to them “You know you might not need to be on PrEP, you know, during the pandemic”. Or “We could hit the pause button for a little bit” so, further thinking about your last question too, I would also say that, that I would totally be open to presenting patients that as an option. Like “Hey let’s stop your PrEP and you know, if you feel like we can restart it at a more appropriate time again, then we can certainly do your baseline labs again and then restart.”

I: Makes sense. Any other thoughts about the COVID pandemic and PrEP

S: I think those are the two biggest things, and then I also do wonder too you know, how honest sometimes patients are about being sexually active during the pandemic. I mean, you know certainly of course there’s a lot of stigma and also you know, also COVID stigma, you know, like “should I be out there, you know, having sex?” And certainly patients still are, um, but you know are they going to be forthcoming to that to their providers. So ,you know, I think it’s just something to approach in a very patient-centered way, um, you know and just having to take that into account too. Just routinely asking patients about their sexual behavior and practices right now, in just a very open-ended way, and just trying to create a safe space with patients being like “you know you can be honest” and things like that. So I do wonder about that.

I: Great. Well that is actually the end of all the questions.
